# Development of bombesin-tubulysin conjugates using multicomponent chemistry to functionalize both the payload and the homing peptide

**DOI:** 10.3389/fphar.2024.1408091

**Published:** 2024-11-12

**Authors:** Dayma Llanes, Robert Rennert, Paul Jänicke, Ibrahim Morgan, Leslie Reguera, Daniel G. Rivera, Manuel G. Ricardo, Ludger A. Wessjohann

**Affiliations:** ^1^ Department of Bioorganic Chemistry, Leibniz Institute of Plant Biochemistry, Halle (Saale), Germany; ^2^ Laboratory of Synthetic and Biomolecular Chemistry, Faculty of Chemistry, University of Havana, Havana, Cuba

**Keywords:** anticancer agents, peptide-drug conjugates (PDCs), tubulysin therapy, lipopeptides, gastrin-releasing peptide (GRP) receptor, bombesin (BBN), bombesin receptor

## Abstract

Peptide-drug conjugates (PDCs) have recently gained significant attention for the targeted delivery of anticancer therapeutics, mainly due to their cost-effective and chemically defined production and lower antigenicity compared to ADCs, among other benefits. In this study, we designed and synthesized novel PDCs by conjugating new thiol-functionalized tubulysin analogs (tubugis) to bombesin, a peptide ligand with a relevant role in cancer research. Two tubulysin analogs bearing ready-for-conjugation thiol groups were prepared by an on-resin multicomponent peptide synthesis strategy and subsequently tested for their stand-alone *in vitro* anti-proliferative activity against human cancer cells, which resulted in IC_50_ values in the nanomolar range. In addition, various fluorescently labeled [K^5^]-bombesin(6–14) peptides, non-lipidated and lipidated with fatty acid chains of variable length, were also synthesized using the versatile multicomponent chemistry. These bombesin derivatives were tested for their gastrin-related peptide receptor (GRPR)-mediated internalization into cancer cells using flow cytometry, proving that the lipid tail (especially C14) enhances the cell internalization. Using the tubugi toxins and bombesin peptides, three different bombesin-tubugi conjugates were synthesized with different cleavage propensity and lipophilicity. Preliminary *in vitro* experiments revealed that, depending on the linker and the presence of a lipid tail, these novel PDCs possess good to potent anticancer activity and moderate selectivity for GRPR-overexpressing cancer cells.

## Graphical Abstract

**Figure:**
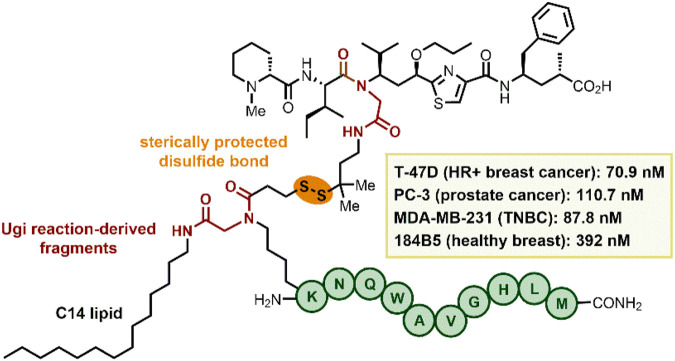
Table of Content Graphic.

## Introduction

Peptide-drug conjugates (PDCs) have emerged in recent years and increasingly become a focus in targeted anticancer drug research (He et al., 2019; Hoppenz et al., 2020). Employing a homing peptide as a cancer cell-targeting entity offers significant benefits over more complex biomolecules such as monoclonal antibodies. These benefits include a well-defined, homogenous and synthetically flexible chemical structure as well as reduced immunogenicity and a more efficient distribution in solid tumors (Hoppenz et al., 2020; Cooper et al., 2021). Additionally, the cost-effective and reproducible methods for synthesizing peptides also make PDCs particularly attractive from both regulatory and manufacturing perspectives. Importantly, many targeting peptides derived from natural peptide ligands exhibit a high affinity and selectivity for cancer-associated cell surface receptors, often G protein-coupled receptors (GPCRs), which makes them promising candidates for targeted tumor therapy approaches (Dorsam and Gutkind, 2007).

**FIGURE 1:**
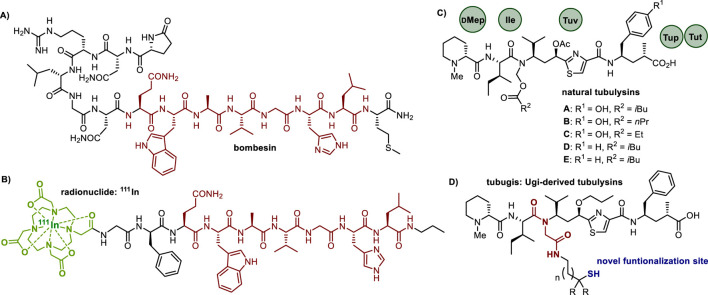
Structures of **(A)** the natural bombesin peptide, showing in red the core sequence Bn (Chen et al., 2014; Worm et al., 2020; Abd-Elgaliel et al., 2008; Ohki-Hamazaki et al., 2005; Begum et al., 2016; Mansi et al., 2021; Steinmetz et al., 2013) compelling for high receptor affinity. **(B)** a synthetic bombesin analog used in radionuclide-peptide conjugates. **(C)** natural tubulysins, highlighting their diverse *N,O*-acetal moieties at the tertiary amide and substitutions of the C-terminal residue. **(D)** synthetic Ugi-reaction derived tubulysin analogs (tubugis) functionalized with a thiol at a new position.

The bombesin peptide (Bn) is a 14-residue neuropeptide originally isolated from the *Bombina bombina* frog (Figure 1A), and its associated receptor family holds significant therapeutic potential in cancer treatment (Anastasi et al., 1972; Faintuch et al., 2008; Chen et al., 2014). The gastrin-releasing peptide receptor (GRPR), in humans the primary and most relevant Bn receptor, is of particular interest due to its overexpression in several prevalent tumors, including those of breast, prostate, lung, and pancreas (Worm et al., 2020). Currently, a series of synthetic Bn analogs comprising simplified sequences are being clinically studied, especially for their application in radionuclide-based conjugates for tumor imaging (Figure 1B). Abd-Elgaliel et al. (2008), Ohki-Hamazaki et al. (2005), Begum et al. (2016), Mansi et al. (2021) While the majority of breakthroughs in this area focuses on diagnostics, Bn analogs also show considerable promise in drug delivery therapies (Anastasi et al., 1972; Faintuch et al., 2008; Chen et al., 2014).

A very crucial aspect in designing an effective PDC is the selection of the payload component, with a prime requirement of having a very potent cytotoxicity. In this context, tubulysins are outstanding due to their cell growth-inhibiting effects in nano to picomolar concentrations (Steinmetz et al., 2013). As depicted in Figure 1C, tubulysins are tetrapeptides composed of unusual residues such as *N*-methyl-D-pipecolic acid (Mep), L-isoleucine (Ile), tubuvaline (Tuv), and either tubuphenylalanine (Tup) or tubutyrosine (Tut).

Despite their potent anti-proliferative activity, tubulysins have been less used in PDC development than other antimitotics, which can be attributed to their complex syntheses and the absence of residues amenable for functionalization with cleavable, ideally traceless linkers (Cooper et al., 2021; Dorsam and Gutkind, 2007; Worm et al., 2020). Early attempts of tubulysin conjugations relied on the linkages of the C-terminal carboxylic acid to entities like vitamins, polymers, antibodies and peptides, mostly via a difficult to cleave amide or a hydrazide moiety (Murray et al., 2015; Kufka et al., 2019; Kaake et al., 2018; Chen et al., 2017; Cohen et al., 2014). Other research groups focused on the insertion of an amino group at Tup’s phenyl moiety or the functionalization of Tuv’s thiazole ring (Parker et al., 2017; Xu et al., 2021). However, the derivatization of the *N*-methyl-D-pipecolic acid into a self-immolating quaternary ammonium linker constitutes the most recurrent alternative, owing to the improved pharmacokinetic and therapeutic efficacy of these conjugates (Rodrigues and Bernardes, 2016; Staben et al., 2016).

Our research is directed at devising suitable directing molecules and linkages for the targeted delivery of potent tubulysin analogs referred to as tubugis (Figure 1D). These analogs were originally obtained by fragment coupling using the Ugi four-component reaction (Ugi-4CR) and they not only exhibit cytotoxic activities in the picomolar concentration range but also provide higher stability under physiological conditions (Pando et al., 2011). While most of the known tubulysin analogs bear a small methyl, ethyl, or propyl *N*-substituent at the tertiary amide (Parker et al., 2017; Leverett et al., 2016), the Ugi-derived *N*-substituent of tubugis replaces the *N*,*O*-acetal with a retro amide residue incorporating alkyl moieties just like the natural products. Since previous studies have shown that the simplification of the *N*-alkyl motif is well accepted to keep a potent cytotoxicity (Sani et al., 2017), we hypothesized that further Ugi-4CR derivatizations could be done, seeking to expand the conjugation possibilities of this relevant payload family. Herein we describe for the first time the functionalization of the Ugi-derived *N*-substituent with a functional group suitable for conjugation to a targeting molecule, specifically a thiol capable of forming a stable conjugate with a homing peptide. Because disulfide bond formation is a successful strategy in the conjugation to targeting biomolecules (Danial and Postma, 2017; Bernardes et al., 2012), we sought to produce PDCs by combining bombesin peptides with tubugis via disulfide linkages, and subsequently evaluate their anticancer potential.

## Results and discussion

### Synthesis of thiol-functionalized tubugis

**Scheme 1:**
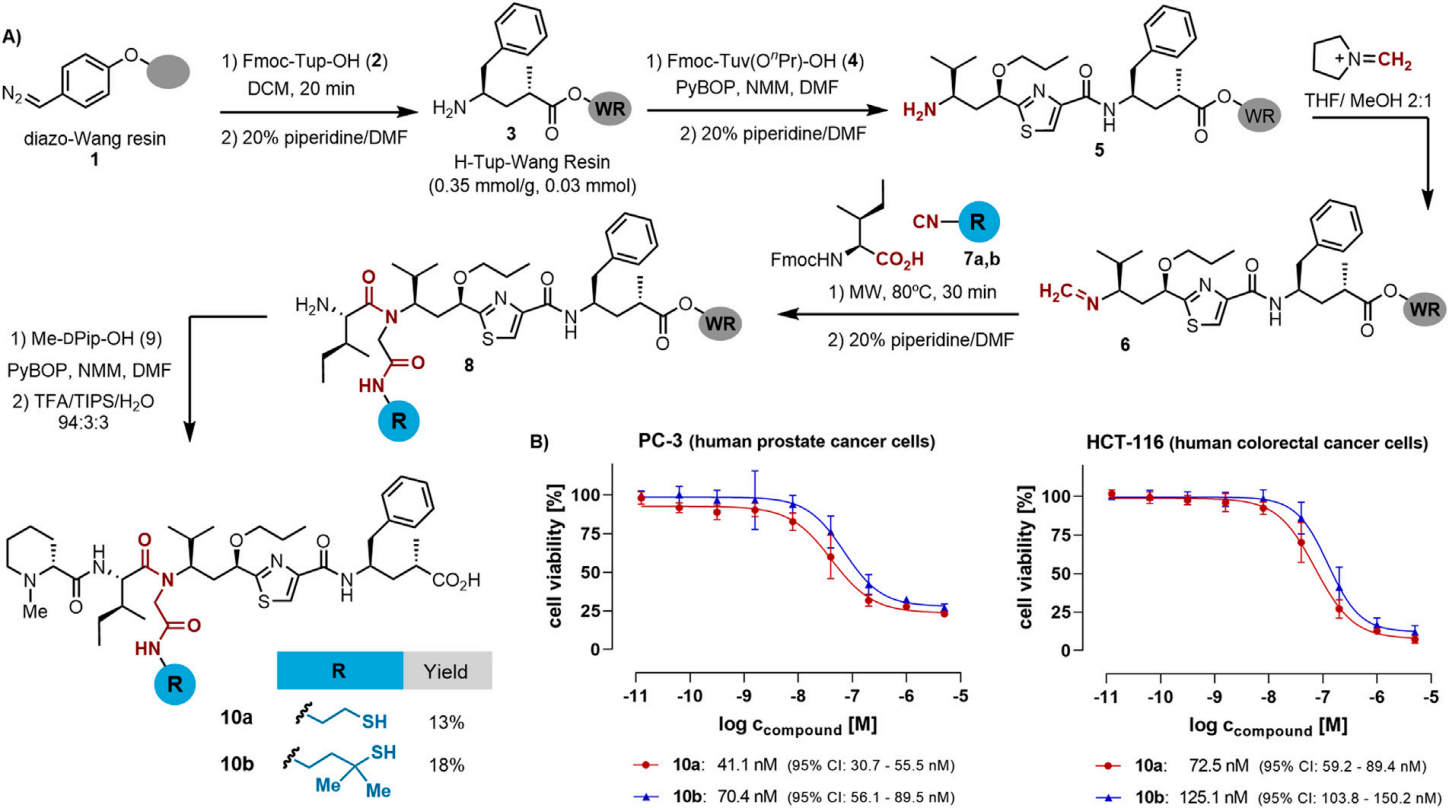
**(A)** Solid-phase synthesis of tubugi analogs functionalized with a thiol group at the internal amide *N*-substituent. WR = Wang resin. **(B)** Evaluation of the anti-proliferative activity: tubugi effects on cell viability, as determined by a resazurin assay for human prostate cancer PC-3 cells and human colon cancer HCT-116 cells treated for 48 h with tubugi **10a** (red dots and line) and tubugi **10b** (blue triangles and line). For control and data normalization, cells were routinely treated in parallel with 0.5% (*v/v*) DMSO (negative control), and as a positive cytotoxicity control with 100 µM digitonin (for data normalization set to 0% cell viability), both in standard growth medium.

The building blocks required for the synthesis of tubulysin derivatives can be purchased commercially or synthesized on gram-scale using standard protocols (Murray et al., 2015; Wang et al., 2019; Shankar et al., 2013; Wipf et al., 2004). However, the assembly of some of these blocks using solution-phase protocols can be challenging due to the steric hindrance of some of them, especially in the coupling step leading to the tertiary amide formation (Peltier et al., 2006; Balasubramanian et al., 2009). We recently developed a solid-phase protocol that enables the construction and multicomponent derivatization of the tubugi skeleton, including the combinatorial diversification of the Ugi-derived *N*-substituent (Ricardo et al., 2024). We applied this protocol to synthesize tubugi derivatives with *N*-substituents bearing a variety of functional groups suitable for conjugation, such as carboxylic acids, amines, and alcohols. However, their introduction led to a significant drop in the anti-proliferative activity, thus limiting their use as PDC payloads. As shown in Scheme 1, here we extend this strategy to generate novel tubugis functionalized with a thiol group at the *N*-substituent, thus paving the way for the subsequent conjugation to a homing peptide without overly reducing the payloads activity.

The synthetic strategy for tubugi analogs begins with the preparation of the diazo resin **1** following a reported protocol (Bhalay and Dunstan, 1998; Lévesque et al., 2017). The resin can be stored at −20°C until further use. A portion of this resin equivalent to a synthesis scale of 0.05 mmol was used to quantitatively incorporate the Tup amino acid **2** without the need to use an excess of this valuable building block. After incorporation of the Tuv residue **4** using standard coupling protocol, the dipeptide was deprotected for the two-step multicomponent protocol. The first step involved an organocatalytic transimination reaction that allowed the quantitative formation of the resin-linked imine **6**, which was subjected to the Ugi-4CR by treatment with thiol-functionalized isocyanides **7** and the Fmoc-protected Ile under microwave irradiation (Rivera et al., 2021; Ricardo et al., 2018). The two isocyanides were synthesized with a thiol moiety protected with an acid-labile group (see the Supplementary Material), bearing geminal methyl groups at the α position or not. Because the final payloads were designed to form a disulfide bond as a linkage to the targeting peptide, the α-substitution was chosen to influence the conjugate stability. The two geminal methyls are known to form rather stable disulfide bonds that only get cleaved in the presence of high concentrations of free thiols like glutathione or similar reductants found under the reducing conditions in cancer cells and solid tumors. After the on-resin Ugi-4CR, *N*-methyl-D-pipecolic acid **9** was coupled and the resulting tubugis **10a** and **10b** were obtained in good crude purity after acidic cleavage from the resin. A final HPLC purification step rendered the pure thiol-functionalized tubugis in good isolated yields considering the number of synthetic steps implemented in the solid-phase protocol.

### Evaluation of anticancer activity

Tubulysin analogs **10a** and **10b** were tested for anti-proliferative activity on the human prostate cancer cell line PC-3 and the colon cancer cell line HCT-116. As illustrated in Scheme 1B, both **10a** and **10b** inhibited the proliferation of the cancer cell lines under investigation with IC_50_ values in the low nanomolar range. Several remarks regarding the activity of the compounds were noted: *i*) the non-methylated analog **10a** showed a 2-fold lower anti-proliferative activity toward the two cell lines than the α-di-methylated analog **10b** (Scheme 1B); *ii*) the prostate cancer PC-3 cells were found to be slightly more sensitive towards both tubugi analogs than the colorectal cancer HCT-116 cells, and *iii*) the compounds’ effect on PC-3 cells seems to be rather cytostatic (lower curve plateau does not reach the 0% level of cell viability), whereas the effect on HCT-116 cells appears to be cytotoxic, reaching nearly 0% cell viability.

### Synthesis and derivatization of bombesin peptides

To design bombesin targeting peptides suitable for PDC applications, structural modifications are required to enable the conjugation without compromising the peptide’s receptor binding and activation. In this context, it has been described that a *N*-terminally truncated bombesin analog [Bn (Faintuch et al., 2008; Chen et al., 2014; Worm et al., 2020; Abd-Elgaliel et al., 2008; Ohki-Hamazaki et al., 2005; Begum et al., 2016; Mansi et al., 2021; Steinmetz et al., 2013; Bouchard et al., 2014): Asn-Gln-Trp-Ala-Val-Gly-His-Leu-Met] mimics the full-length bombesin’s ability to activate bombesin receptors, particularly the human bombesin-binding gastrin-releasing peptide receptor (GRPR) (Begum et al., 2016). This study also proved that adding a Lys residue at the *N*-terminus of this truncated sequence permits the subsequent derivatization at the Lys ɛ-amino group without affecting the internalization capacity. Thus, by tagging this Lys residue with a fluorescein label, significant agonist activity and high levels of internalization were observed, particularly in prostate cancer cells (Begum et al., 2016).

To validate this [K^5^]-Bn(6–14) sequence as a targeting peptide, we synthesized various truncated bombesins and compared their internalization in the prostate cancer PC-3 cells by conducting a flow cytometry analysis. Our approach was influenced by similar studies on other targeting peptides, e.g., the human pancreatic polypeptide (hPP), (Mäde et al., 2014), for which enhanced cell internalization was achieved by increasing the hydrophobicity through attachment of a fatty acid. Given that multicomponent reactions are powerful approaches for the one-pot incorporation of various functionalities, we relied on a multicomponent strategy for the on-resin derivatization of the bombesin analogs with a lipid and a fluorescent label simultaneously. To minimize a potential negative impact of a large hydrophobic label (e.g., fluorescein) on the ligand-receptor binding and cell internalization, we chose to incorporate the considerably smaller 7-nitro-benzofurazan (NBD) fluorophore.

**Scheme 2:**
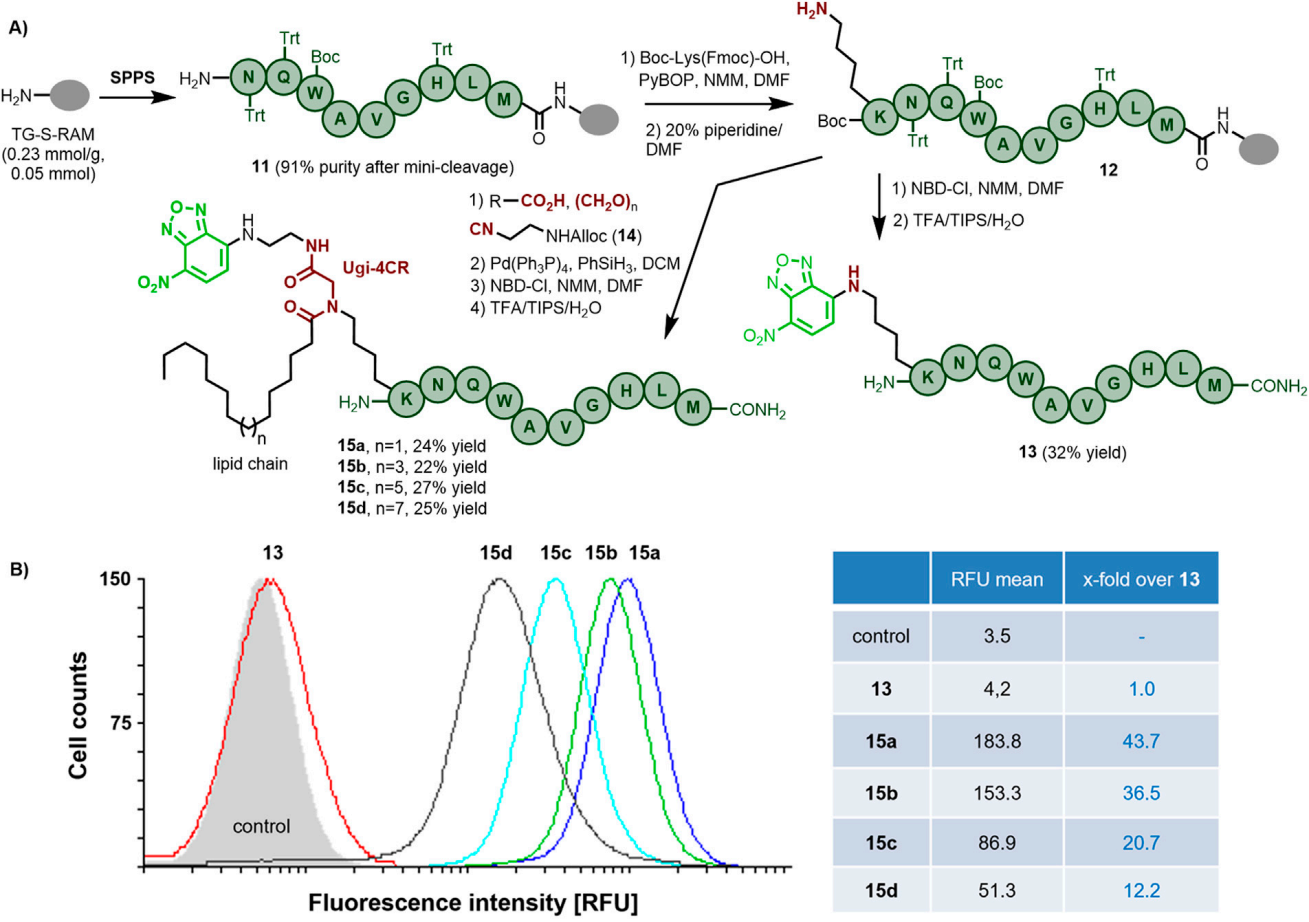
**(A)** Synthesis of fluorescently-labeled bombesin derivatives incorporating lipid chains of different lengths. **(B)** Flow cytometry analysis of the internalization of the bombesin derivatives **13** (reference without lipid tail), **15a**, **15 b**, **15c** and **15d** in human prostate cancer PC-3 cells. The cells were treated for 2 h (for other time points see the SI, Supplementary Figure S46) with 5 µM concentrations of the peptides at 37 °C.

As depicted in Scheme 2A, the truncated sequence Bn(6–14) **11** was assembled on a Tentagel resin (0.05 mmol scale), followed by the incorporation of Boc-Lys(Fmoc)-OH using the standard SPPS methodology to afford the resin-bound [K^5^]-Bn(6–14) **12**. The non-lipidated analog **13** was obtained in 32% overall yield after a final reaction of NBD-Cl with the ɛ-amino group of Lys and subsequent standard cleavage with TFA/TIPS/H_2_O (94:3:3). For the incorporation of both the fatty acid and the fluorescent label, we employed an on-resin Ugi-4CR comprising the aminocatalytic transimination approach followed by reaction of the resulting imine with various fatty acids and isocyanide **14**, bearing an orthogonally (Alloc) protected amino group (Rivera et al., 2021; Ricardo et al., 2018). The parallel incorporation of four fatty acids of different chain lengths (C14, C16, C18, and C20) followed by Alloc deprotection, incorporation of the NBD label and cleavage from the resin led to bombesin analogs **15a**, **b, c**, and **d** in good overall yield after HPLC purification (see the Supplementary Material).

### Internalization study of bombesin peptides

The cellular internalization of the NBD-labeled bombesin derivatives without (**13**) and with lipid tails of varied lengths C14-C20 (**15a**, **15b**, **15c**, and **15d**) into GRPR-expressing PC-3 prostate cancer cells were semi-quantified by flow cytometry. Trypan blue quenching of outer cell surface fluorescence was done immediately before and while measuring, aiming to inspect as much as possible truly intracellular, hence internalized fluorescence of the labeled bombesin analogs (Rennert et al., 2009). The results of the flow cytometry measurements are illustrated in Scheme 2B and Supplementary Figure S46. As shown, the non-lipidated bombesin analog **13** showed only a poor cell internalization within the 2 h of treatment, as indicated by the just slightly elevated fluorescence count (RFU 4.2 compared to 3.5 of untreated control cells, this base value is due to their autofluorescence) caused by the intracellularly located peptide. On the other hand, all lipidated analogs were internalized better than the non-lipidated one, even within a short treatment time of only 2 h. Interestingly, a reverse correlation between the alkyl chain length and the internalization potency was observed, making **15a** (C14 chain) the most efficiently internalized bombesin analog with a 43.7-fold higher intracellular fluorescence compared to **13**, followed by **15b** (C16), **15c** (C18), and **15d** (C20) with decreasing 36.5-fold, 20.7-fold, and 12.2-fold higher intracellular fluorescence as compared to **13**. Previously, other groups have reported a similar trend for membrane-active lipopeptides, where C6–C12 acyl chains improved the lipopeptide interaction with the cell membranes better than longer and shorter lipid tails (Toniolo et al., 1996). As summarized in Supplementary Figure S46, the time dependency of the bombesin analogs’ cellular internalization was investigated as well, indicating a fast cell internalization process for all lipidated bombesins, with substantial internalization already within the first minute of treatment, reaching the majority of internalized ligand after just 1 hour, and with little increase to a maximum within the second hour. Such a fast cell internalization kinetics is typical for several G protein-coupled receptor peptide ligands. For example, the data of another GPCR-ligand system, the neuropeptide Y Y_1_ receptor (NPY1R), also shows the GPCR rapidly internalizing the peptide ligand NPY or agonistic analogs within minutes. (Gicquiaux et al., 2002; Babilon et al., 2013). However, for the GRPR receptor, at least in our hands, fast internalization is only achieved with the lipidated peptide analogs, and not with the non-lipidated ligand **13**.

### Synthesis of bombesin-tubugi conjugates via a disulfide linkage

**Scheme 3:**
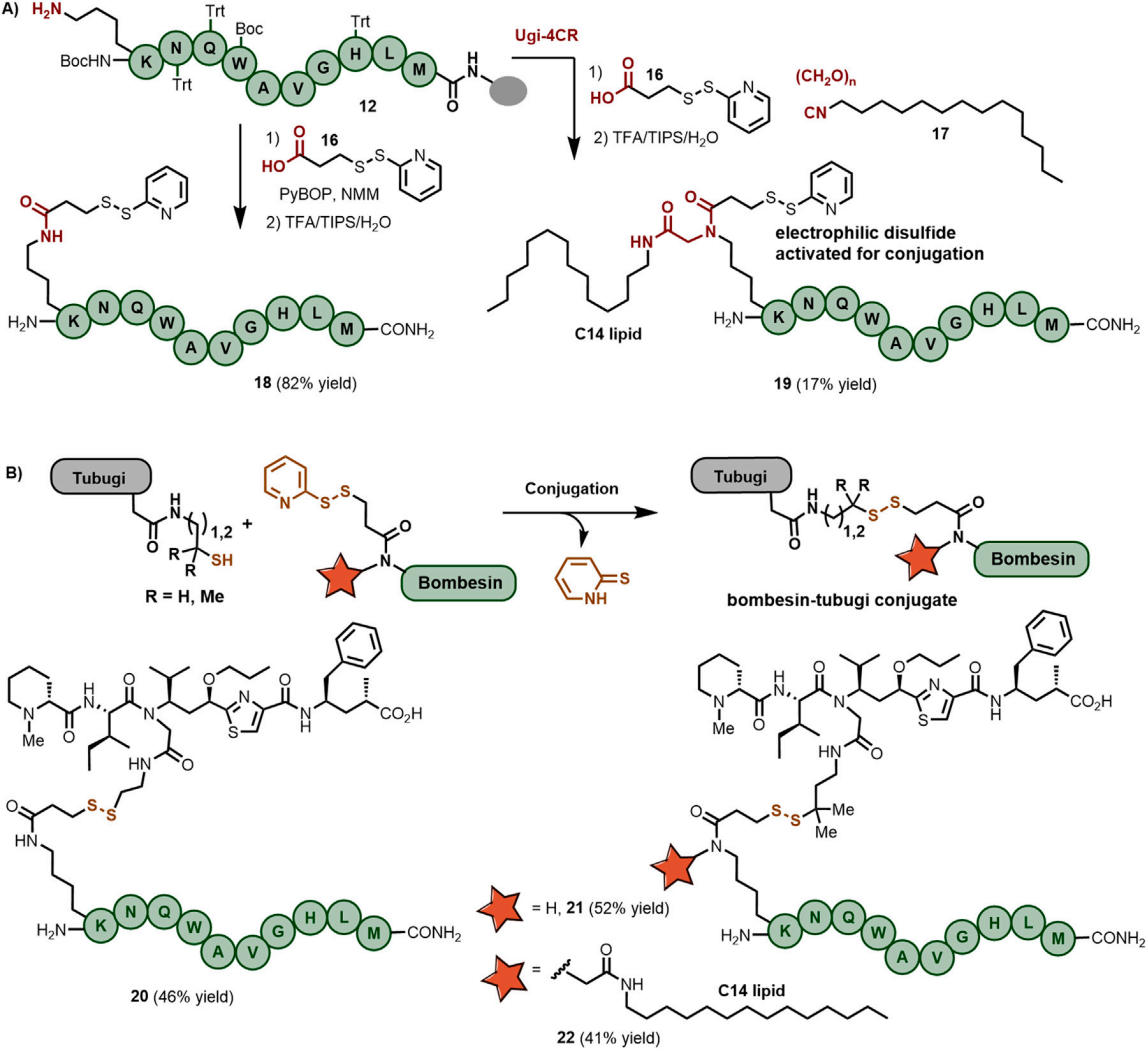
**(A)** On-resin synthesis of 2-pyridyl disulfide-functionalized bombesin peptides either containing a C14 lipid chain or not. **(B)** Bombesin-tubugi conjugates produced by disulfide bond formation between activated bombesin and thiol-functionalized tubugis.

Disulfide bond formation is widely used in the field of peptide derivatization, especially in dimerization processes and side chain crosslinking involving Cys residues (Danial and Postma, 2017; Deng et al., 2020). However, the formation of asymmetric disulfide bonds is of greater synthetic challenge and often requires thiol exchange in electrophilic disulfides, typically using either Elman’s reagent or 2-pyridyl disulfides (Bernardes et al., 2012; Pillow et al., 2016). As shown in Scheme 3A, the bombesin peptide was functionalized at the Lys side chain using 2-pyridyl disulfide containing a carboxylic acid (Beck et al., 2017) derived from 3-mercaptopropionic acid. The integrity of the 2-pyridyl disulfide component remained unaffected by the acidic conditions used during cleavage from the resin, leading to the activated bombesin analog **18** in 82% overall yield and 87% crude purity without the need for HPLC purification. Because of the superior internalization performance of the lipidated bombesin in cancer cells, i.e., derivative **15a** with a C14 chain, we aimed to functionalize the truncated Bn with both a 2-pyridyl disulfide linker and a C14 lipid chain. For this purpose, lipidic isocyanide **17**, paraformaldehyde and linker **16** were simultaneously incorporated at the Lys side chain using the Ugi-4CR (Scheme 3A), yielding bombesin derivative **19** in 17% isolated yield after RP-HPLC purification.

Integrating the activated disulfide into the bombesin peptide streamlined the solution-phase coupling with the thiol-functionalized tubugi. As depicted in Scheme 3B, bombesin analog **18** was ligated to the thiol-tubugi **10a** in MeOH/DIPEA, with full consumption of starting materials after 2 h, leading to bombesin-tubugi conjugate **20** in 46% yield after purification. Following a similar conjugation protocol, conjugate **21** was produced in 52% isolated yield, while the preparation of the lipidated bombesin-tubugi conjugate required 12 h to reach complete consumption of the starting materials, ultimately giving **22** in 41% isolated yield.

### Anti-proliferation study of bombesin-tubugi conjugates

The bombesin-tubugi conjugates **20**, **21**, and **22** were tested on human cell lines representing various receptor expression levels of GRPR, a human receptor with a high affinity for bombesin. We sought to investigate the correlation between the anti-proliferative efficacy of the conjugates and the GRPR expression levels of the cells, hypothesizing a better anti-proliferative effect in the cells with the higher expression level. For this purpose, a panel of human, primarily cancer, cell lines was initially selected to encompass a broad spectrum of GRPR expression levels. The selection process was based on differences in mRNA expression levels of GRPR as documented in the publicly available RNA-Seq database, GRCh38.p12 (O’Leary et al., 2024). Differential expression visualization was performed using the Genevestigator® gene expression analysis tool (Hruz et al., 2008), aiming to cover a wide range of GRPR expression levels. Accordingly, the following cell lines were selected for testing: T-47D (HR+ breast cancer), MDA-MB-231 (triple-negative breast cancer, TNBC), PC-3 (prostate cancer), MCF-10A (non-cancer, “healthy” breast epithelium), and HCT-116 (colorectal cancer). Of notice, T-47D has been reported with relatively high, MDA-MB-231 and PC-3 with medium, and HCT-116 and MCF-10A with relatively low GRPR expression levels (decreasing in the order of that list).

**FIGURE 2:**
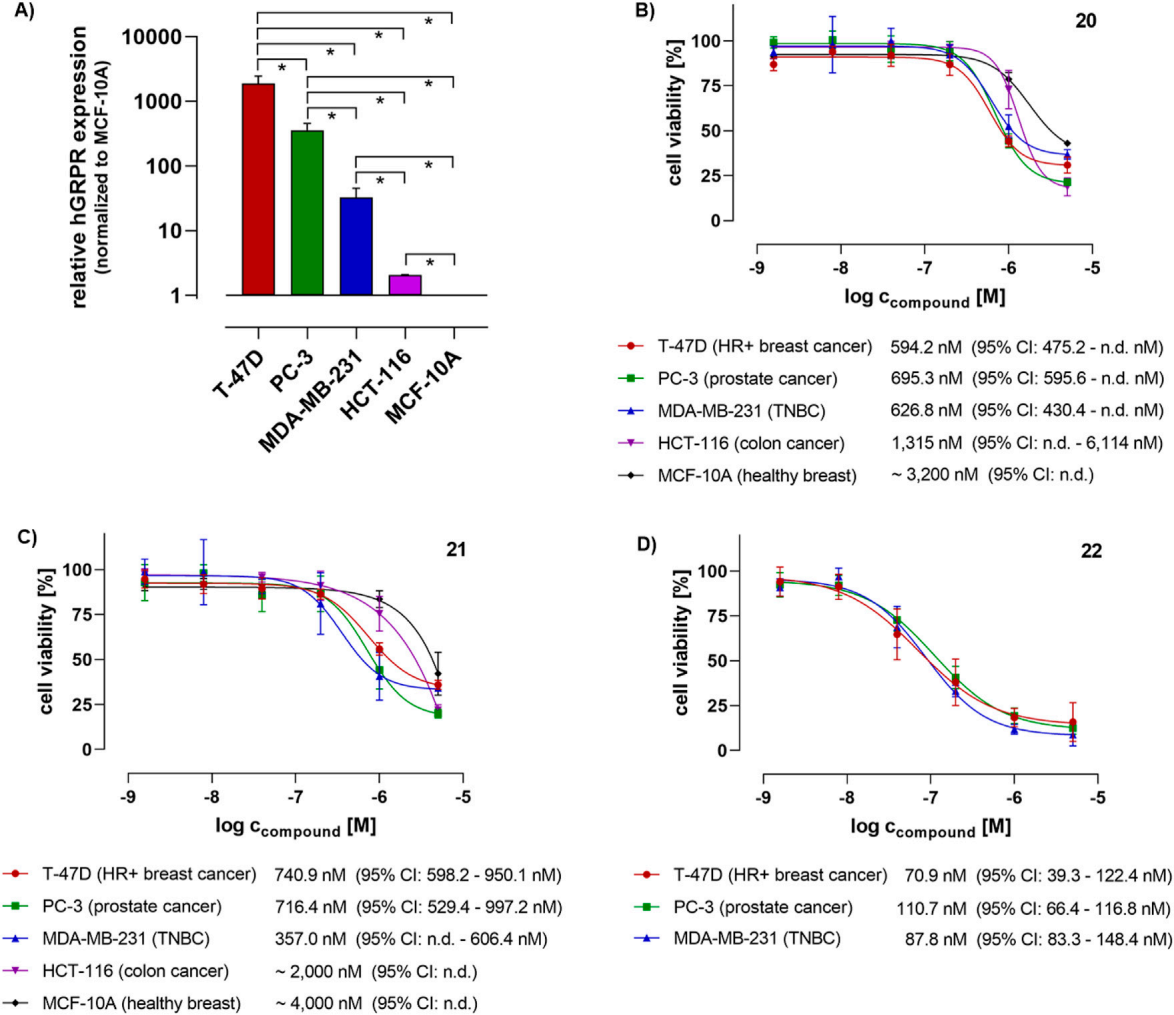
**(A)** Relative human GRPR (mRNA) expression levels in the cell lines used for the flow cytometry and cell viability studies, as detected by using RT-qPCR (n = 3). Data were analyzed by conducting the ΔΔCt methodology using GAPDH as a reference gene and normalized to the GRPR expression level of non-cancerous MCF-10A breast cells as a “healthy” cell reference (set to 1.0). Statistical significance was tested by using an unpaired t-test (*p < 0.05). **(B–D)** Cell viability of human cancer (T-47D, PC-3, MDA-MB-231, and HCT-116) and non-cancer (MCF-10A) cell lines after 6 h initial treatment with the bombesin-tubugi conjugates **20** (normal disulfide), **21** (hindered disulfide), and **22** (hindered disulfide and lipidated), medium exchange and subsequent cell cultivation until finalization of 72 h, and subsequent measurement by using fluorometric resazurin assay read-out. For control and data normalization, cells were routinely treated in parallel with 0.5% (*v/v*) DMSO (negative control), and as a positive cytotoxicity control with 100 µM digitonin (for data normalization set to 0% cell viability), both in standard growth medium. Each data point was determined with 3-4 biological replicates, each with technical triplicates. The indicated concentrations are IC_50_ values as calculated based on the non-linear regression dose-response curves as drawn by using GraphPad Prism 8 software. Error bars indicate the 95% confidence interval.

The actual GRPR expression levels of these cell lines were checked by conducting RT-qPCR, as described in the SI. Indeed, as shown in Figure 2A, the five cell lines ranked in the proposed order described above. The cell viability and proliferation assays were carried out by using a resazurin-based fluorometric assay. The cells were exposed to the bombesin conjugates **20**, **21**, and **22** for initial 6 h and 48 h, and regardless of the initial incubation time, the cells were then allowed to grow until 72 h in sum were finalized, after which the cell viability was determined (see the SI).

The results of the cell viability assays indicating the anti-proliferative and cytotoxic effects of the bombesin-tubugi conjugates are shown in Figures 2B–D (conjugate **20**–**22**, respectively) for the initial 6 h treatment, and in the SI (Supplementary Figures S7, S48, S49) for the 48 h treatment. Because of the rather short metabolic half-lives of peptides *in vivo*, usually minutes up to just a few hours, the 6 h initial treatment was thought to be of higher relevance for potential anticancer therapeutics. The 48 h treatment was intended to show a proposed maximum effect of the sample. As illustrated in Figure 2B, after only 6 h initial cell treatment, conjugate **20** showed an anticancer activity in the high nanomolar range, as proven by an IC_50_ ∼ 600 nM in T-47D breast cancer cells, and just slightly higher IC_50_ values in PC-3 prostate and MDA-MB-231 TNBC cancer cells. Furthermore, IC_50_ values approximately 2-fold and 5.5-fold higher were detected in the GRPR-lower expressing HCT-116 (IC_50_ ∼ 1.3 µM) and GRPR-lowest expressing, healthy MCF-10A cells (IC_50_ ∼ 3.2 µM), respectively. Although the anticancer activity of conjugate **20** is not remarkable, the data illustrate a direct correlation between the cells’ GRPR expression levels and the anti-proliferative efficacy. The results of conjugate **21** after 6 h initial treatment (as shown in Figure 1C) are quite comparable to those described above for conjugate **20**. The IC_50_ values of **21** are slightly higher in the different cell lines than for **20**, except for the MDA-MB-231 cell line. In this latter cell line, conjugate **21** showed an IC_50_ ∼ 360 nM, whereas the IC_50_s in the higher expressing T-47D and PC-3 cancer cells were determined to be 741 nM and 716 nM, respectively. However, compared to these values, IC_50_ values of **21** in the lower and lowest GRPR-expressing HCT-116 cancer and MCF-10A healthy breast cells were approximately 2.5- and 5.5-fold higher, respectively. Compared to the lowest IC_50_ of **21** in MDA-MB-231 (∼360 nm), the IC_50_s of the conjugate in HCT-116 and MCF-10A were even ∼10-fold higher.

Interestingly, in the case of both **20** and **21** treatments for the initial 6 h, a significant difference in IC_50_s between the three highest and medium GRPR-expressing cell lines - i.e., T-47D, PC-3, and MDA-MB-231 – was not detected, which is in contrast to what was expected. Seemingly, there might be a certain GRPR expression level that allows a maximum targeting effect of the PDC, here IC_50_ ∼ 600–700 nM after 6 h initial treatment, that is already reached at least with the medium GRPR expression level of MDA-MB-231 cells. This could imply that even far higher GRPR levels would not have a substantial benefit for the targeting and thus selective cytotoxic effect.

After the cell treatment for 48 h with both conjugates **20** and **21**, all IC_50_ values were measured in the nanomolar range, as well as those against the lower GRPR-expressing cell lines HCT-116 and MCF-10A. The reason for this could be the time that cells have to import the conjugates via GRPR-mediated endocytosis in multiple receptor internalization and recycling cycles. This could also be the reason for the lack of a clear correlation between GRPR expression levels and the conjugates’ anti-proliferative impact after 48 h initial incubation, as indicated by a difference of the lowest (in T-47D; IC_50_ ∼ 200 nM) and highest IC_50_ (in HCT-116; IC_50_ ∼ 570 nM) of at most ∼2.5-fold. This has, of course, consequences for the cancer cell selectivity of the bombesin conjugates, and should be kept in mind in future developments of bombesin-toxin conjugates. Considering the high similarity of the conjugates **20** and **21** in terms of chemical structure, *in vitro* cytotoxicity (IC_50_ values) and selectivity, the intended stabilization of the disulfide bridge by the dimethylation in **21** seems to have only a very modest effect on the PDC efficacy, at least *in vitro*.

As illustrated in Figure 1D (see also Supplementary Figure S49) the C14-lipidated conjugate **22** was 4- to 10-fold more potent in three cancer cells overexpressing the GRPR than the non-lipidated conjugates **20** and **21**, respectively, with IC_50_ values in the range of ∼70–110 nM. We also conducted a direct comparison of conjugate **22** and tubugi **10b** with an initial treatment of the cells for 6 h, as it makes sense to compare a PDC with its free payload. Besides the cancer cells, both compounds were tested (Supplementary Figure S50) in another healthy breast cell line, 184B5, which has a GRPR (mRNA) expression level that is comparable to that of MCF-10A, according to the Genevestigator^®^ gene expression analysis tool (Hruz et al., 2008). As summarized in Table 1, the bombesin conjugate **22** exhibited a higher cytotoxicity than free tubugi **10b** in the three cancer cells, but compound **10b** also proved to be three times more toxic than conjugate **22** in the 184B5 healthy cells. In this case, the lack of selectivity of tubugi **10b** among the cell lines tested is expected, given that this small compound can have a similar internalization efficiency in all cells after a short initial treatment of 6 h. It is also noteworthy the selectivity of conjugate **22**, which exhibited a 3.5- to 5.5-fold higher cytotoxicity for cells overexpressing the GRPR receptor (Figure 1D) than for healthy 184B5 cells (Supplementary Figure S50). While this level of selectivity is not yet satisfactory for therapeutic applications, it is a clear indication of what can be achieved using this strategy.

**TABLE 1:** Comparison of the anti-proliferative activity of bombesin-tubugi conjugate **22** and its free tubugi payload **10b** in cancer and healthy cells after 6 h of initial treatmment.

Cell line	IC_50_s [nM]	
	PDC 22	Tubugi 10b
T-47D	70.9	150.3
PC-3	110.7	231.9
MDA-MB-231	87.8	121.3
184B5 (healthy)	391.6	116.7

### Study limitations

While this study represents the first report on the development of bombesin-derived PDCs incorporating tubulysin analogs, we acknowledge that our approach has some limitations derived from both the type of receptor targeted and the methodology employed (Heh et al., 2023). For example, the initial cytotoxicity evaluation of the tubulysin analogs **10a** and **10b** was conducted using only the PC-3 and HCT-116 cells, with 48 h of initial treatment, as part of a general screening program in our laboratory. Accordingly, these results have limited comparability to the data obtained for the PDCs **20**, **21** and **22**, for which additional cancer cells and an initial treatment of 6 h were employed (as recommended for PDC evaluation). Fortunately, we were able to compare PDC **22** and its parent unconjugated payload **10b** using 6 h of initial treatment and the same cell lines, which provided valuable information regarding the selectivity achieved by the bombesin PDC compared to the free toxin (discussed above). A further limitation is the lack of sufficient data concerning the role of lipidation on the PDC behavior. In addition to addressing PDC disadvantages such as poor metabolic stability and rapid renal clearance, lipidation also increases the PDC’s interaction with the cell membrane. The impact of this can be beneficial or detrimental, depending on whether it increases the PDC concentration on the cell membrane where the receptors are localized or also boosts an undesired, non-receptor-mediated internalization process. We did a comparative internalization analysis with the labeled bombesin peptide bearing various lipid chain lengths or no lipid chain, but assessing the internalization behavior of the final PDCs on different cell lines is further required.

Another limitation can be associated with the use of GRPR as target, since its level of overexpression in specific cancer cells might be insufficient to achieve a suitable selectivity index. The GRPR is known to be overexpressed in various cancer cells (D’Onofrio et al., 2023; Belge et al., 2024; Zhang et al., 2024), which was corroborated by our RT-qPCR data, but - like other GPCRs - it is not absent in healthy cells and tissues. It is also known that agonistic GPCR activation results in a subsequent desensitization and downregulation of the receptor, albeit with a certain delay. This also applies to the GRPR targeted by our PDCs (Benya et al., 1994). Furthermore, we don’t have data demonstrating the agonistic behavior of our PDCs or the lipidated bombesins used in the internalization study. The flow cytometry analysis in PC-3 cells of the bombesin derivatives (Scheme 2) indicated a fast and efficient cellular internalization of the lipidated bombesins, but other internalization mechanims cannot be ruled out. Overall, as expected, lipidated derivatives showed better internalization and thus higher bioactivity (lower IC_50_s). Indeed, new experiments focus on assessing both the capacity of our PDCs to functionally activate GRPR and the effectiveness of a receptor-mediated payload delivery into the cancer cells. This will help either to confirm or exclude alternative mechanisms of internalization for the lipidated PDC.

## Conclusion

A novel solid-phase methodology was implemented for the synthesis of tubulysin analogs, known as tubugis, functionalized with ready-to-conjugate thiol groups. The on-resin protocol incorporates a key Ugi multicomponent reaction that allows the simultaneous assembly of the tubulysin peptide skeleton and the incorporation of the thiol-functionalized amide *N*-substituent. In parallel, cell internalization studies conducted with a fluorescently labeled bombesin peptide (active truncated sequence) proved that the peptide lipidation increases the homing peptide internalization, with a C14 chain showing the best cell internalization. As a result, a Ugi multicomponent approach was employed for the on-resin functionalization of the truncated bombesin with both a 2-pyridyl-activated disulfide and a C-14 lipid tail. Both lipidated and non-lipidated bombesin peptides were conjugated to the thiol-tubugi payloads and the resulting conjugates were evaluated for their anti-proliferative activity in a variety of cancer cells expressing GRPR (bombesin receptor) at different levels. The lipidated bombesin-tubugi **22** showed the best anti-proliferative activity and a good selectivity for cancer cells, although its selectivity is not sufficient to be considered for therapeutic applications. The present results do not yet allow for the establishment of a suitable correlation between the receptor expression levels and the cytotoxic activity of the PDCs. There appears to be an early saturation, with higher expressen not having additional benefits. Apparently, our data reflect a saturation of the receptor-mediated PDC internalization, causing higher GRPR expression not having additional benefit. However, the data suggest that the lipidation plays an important role in the PDC behavior, and the possibility of non-specific internalization of the lipidated bombesin and its conjugate cannot be ruled out, thereby circumventing receptor-mediated uptake and reducing selectivity. Further studies are required to get deeper insights into the mechanism of action of (lipidated) bombesin peptides as targeting molecules, and the potential of this conjugate class in targeted anticancer therapy.

## Data Availability

The original contributions presented in the study are included in the article/Supplementary Material. Further inquiries can be directed to the corresponding authors.

## References

[B1] Abd-Elgaliel W. R. Gallazzi F. Garrison J. C. Rold T. L. Sieckman G. L. Figueroa S. D. (2008). Design, synthesis, and biological evaluation of an antagonist-bombesin analogue as targeting vector. Bioconjug Chem. 19, 2040–2048. 10.1021/bc800290c 18808168 PMC2659627

[B2] Anastasi A. Erspamer V. Bucci M. (1972). Isolation and amino acid sequences of alytesin and bombesin, two analogous active tetradecapeptides from the skin of European discoglossid frogs. Arch. Biochem. Biophys. 148, 443–446. 10.1016/0003-9861(72)90162-2 4537042

[B3] Babilon S. Mörl K. Beck-Sickinger A. G. (2013). Towards improved receptor targeting: anterograde transport, internalization and postendocytic trafficking of neuropeptide y receptors. Biol. Chem. 394, 921–936. 10.1515/hsz-2013-0123 23449522

[B4] Balasubramanian R. Raghavan B. Begaye A. Sackett D. L. Fecik R. A. (2009). Total synthesis and biological evaluation of tubulysin U, tubulysin V, and their analogues. J. Med. Chem. 52, 238–240. 10.1021/jm8013579 19102699 PMC4183140

[B5] Beck A. Goetsch L. Dumontet C. Corvaïa N. (2017). Strategies and challenges for the next generation of antibody–drug conjugates. Nat. Rev. Drug Discov. 16, 315–337. 10.1038/nrd.2016.268 28303026

[B6] Begum A. A. Moyle P. M. Toth I. (2016). Investigation of bombesin peptide as a targeting ligand for the gastrin releasing peptide (GRP) receptor. Bioorg Med. Chem. 24, 5834–5841. 10.1016/j.bmc.2016.09.039 27670095

[B7] Belge B. G. Bilgin C. Orscelik A. Burkett B. J. Thorpe M. P. Johnson D. R. (2024). Detection rate of gastrin-releasing peptide receptor (GRPr) targeted tracers for positron emission tomography (PET) imaging in primary prostate cancer: a systematic review and meta-analysis. Ann. Nucl. Med. 38, 865–876. 10.1007/s12149-024-01978-6 39287742

[B8] Benya R. V. Fathi Z. Kusui T. Pradhan T. Battey J. F. Jensen R. T. (1994). Gastrin-releasing peptide receptor-induced internalization, down-regulation, desensitization, and growth: possible role for cyclic AMP. Mol. Pharmacol. 46 (2), 235–245. 8078487

[B9] Bernardes G. J. L. Casi G. Trüssel S. Hartmann I. Schwager K. Scheuermann J. (2012). A traceless vascular-targeting antibody-drug conjugate for cancer therapy. Angew. Chem. - Int. Ed. 51 (51), 941–944. 10.1002/anie.201106527 22173886

[B10] Bhalay G. Dunstan A. R. (1998). Facile solid phase synthesis of an activated diazo linker. Tetrahedron Lett. 39, 7803–7806. 10.1016/S0040-4039(98)01706-7

[B11] Bouchard H. Viskov C. Garcia-Echeverria C. (2014). Antibody – drug conjugates — a new wave of cancer drugs. Bioorg Med. Chem. Lett. 24, 5357–5363. 10.1016/j.bmcl.2014.10.021 25455482

[B12] Chen H. Lin Z. Arnst K. E. Miller D. D. Li W. (2017). Tubulin inhibitor-based antibody-drug conjugates for cancer therapy. Molecules 22, 1281. 10.3390/molecules22081281 28763044 PMC6152078

[B13] Chen H. Wan S. Zhu F. Wang C. Cui S. Dua C. (2014). A fast tumor-targeting near-infrared fluorescent probe based on bombesin analog for *in vivo* tumor imaging. Contrast Media Mol. Imaging 9, 122–134. 10.1002/cmmi.1545 24523057

[B14] Cohen R. Vugts D. J. Visser G. W. M. Walsum M. S. Bolijn M. Spiga M. (2014). Development of novel ADCs: conjugation of tubulysin analogues to trastuzumab monitored by dual radiolabeling. Cancer Res. 74, 5700–5710. 10.1158/0008-5472.CAN-14-1141 25145670

[B15] Cooper B. M. Iegre J. O’Donovan D. H. Ölwegård Halvarsson M. Spring D. R. (2021). Peptides as a platform for targeted therapeutics for cancer: peptide-drug conjugates (PDCs). Chem. Soc. Rev. 50, 1480–1494. 10.1039/d0cs00556h 33346298

[B16] Danial M. Postma A. (2017). Disulfide conjugation chemistry: a mixed blessing for therapeutic drug delivery? Ther. Deliv. 8, 359–362. 10.4155/tde-2017-0003 28530144

[B17] Deng Z. Hu J. Liu S. (2020). Disulfide-based self-immolative linkers and functional bioconjugates for biological applications. Macromol. Rapid Commun. 41, 1900531. 10.1002/marc.201900531 31755619

[B18] D’Onofrio A. Engelbrecht S. Läppchen T. Rominger A. Gourni E. (2023). GRPR-targeting radiotheranostics for breast cancer management. Front. Med. (Lausanne) 10, 1250799. 10.3389/fmed.2023.1250799 38020178 PMC10657217

[B19] Dorsam R. T. Gutkind J. S. (2007). G-protein-coupled receptors and cancer. Nat. Rev. Cancer 7, 79–94. 10.1038/nrc2069 17251915

[B20] Faintuch B. L. Teodoro R. Duatti A. Muramoto E. Faintuch S. Smith C. J. (2008). Radiolabeled bombesin analogs for prostate cancer diagnosis: preclinical studies. Nucl. Med. Biol. 35, 401–411. 10.1016/j.nucmedbio.2008.02.005 18482677

[B21] Gicquiaux H. Lecat S. Gaire M. Dieterlen A. Mély Y. Takeda K. (2002). Rapid internalization and recycling of the human neuropeptide Y Y1 receptor. J. Biol. Chem. 277, 6645–6655. 10.1074/jbc.M107224200 11741903

[B22] He R. Finan B. Mayer J. P. Dimarchi R. D. (2019). Peptide conjugates with small molecules designed to enhance efficacy and safety. Molecules 24, 1855. 10.3390/molecules24101855 31091786 PMC6572008

[B23] Heh E. Allen J. Ramirez F. Lovasz D. Fernandez L. Hogg T. (2023). Peptide drug conjugates and their role in cancer therapy. Int. J. Mol. Sci. 24, 829–844. 10.3390/ijms24010829 36614268 PMC9820985

[B24] Hoppenz P. Els-Heindl S. Beck-Sickinger A. G. (2020). Peptide-drug conjugates and their targets in advanced cancer therapies. Front. Chem. 8, 571. 10.3389/fchem.2020.00571 32733853 PMC7359416

[B25] Hruz T. Laule O. Szabo G. Wessendorp F. Bleuler S. Oertle L. (2008). Genevestigator v3: a reference expression database for the meta-analysis of transcriptomes. Adv. Bioinforma. 2008, 420747. 10.1155/2008/420747 PMC2777001 19956698

[B26] Kaake M. Srinivasarao M. Low P. S. (2018). Targeted tubulysin B hydrazide conjugate for the treatment of luteinizing hormone-releasing hormone receptor-positive cancers. Bioconjugate Chem. 29, 2208–2214. 10.1021/acs.bioconjchem.8b00164 29851465

[B27] Kufka R. Rennert R. Kalu G. N. Weber L. Richter W. Wessjohann L. A. (2019). Synthesis of a tubugi-1-toxin conjugate by a modulizable disulfide linker system with a neuropeptide Y analogue showing selectivity for hY1R-overexpressing tumor cells. Beilstein J. Org. Chem. 15, 96–105. 10.3762/bjoc.15.11 30680044 PMC6334802

[B28] Leverett C. A. Sukuru S. C. K. Vetelino B. C. Musto S. Parris K. Loganzo F. (2016). Design, synthesis, and cytotoxic evaluation of novel tubulysin analogues as ADC payloads. ACS Med. Chem. Lett. 7, 999–1004. 10.1021/acsmedchemlett.6b00274 27882198 PMC5108041

[B29] Lévesque É. Laporte S. T. Charette A. B. (2017). Continuous flow synthesis and purification of aryldiazomethanes through hydrazone fragmentation. Angew. Chem. Int. Ed. 56, 837–841. 10.1002/anie.201608444 27936304

[B30] Mäde V. Bellmann-Sickert K. Kaiser A. Meiler J. Beck-Sickinger A. G. (2014). Position and length of fatty acids strongly affect receptor selectivity pattern of human pancreatic polypeptide analogues. ChemMedChem 9 (9), 2463–2474. 10.1002/cmdc.201402235 25156249 PMC5518309

[B31] Mansi R. Nock B. A. Dalm S. U. Busstra M. B. van Weerden W. M. Maina T. (2021). Radiolabeled bombesin analogs. Cancers (Basel) 13, 5766. 10.3390/cancers13225766 34830920 PMC8616220

[B32] Murray B. C. Peterson M. T. Fecik R. A. (2015). Chemistry and biology of tubulysins: antimitotic tetrapeptides with activity against drug resistant cancers. Nat. Prod. Rep. 32, 654–662. 10.1039/c4np00036f 25677951

[B33] Ohki-Hamazaki H. Iwabuchi M. Maekawa F. (2005). Development and function of bombesin-like peptides and their receptors. Int. J. Dev. Biol. 49, 293–300. 10.1387/ijdb.041954ho 15906244

[B53] O’Leary N. A. Cox X. Holmes J. B. Anderson W. R. Falk R. Hem V. (2024). Exploring and retrieving sequence and metadata for species across the tree of life with NCBI Datasets. Sci Data. 11 (1), 732. 10.1038/s41597-024-03571-y 38969627 PMC11226681

[B34] Pando O. Stark S. Denkert A. Porzel A. Preusentanz R. Wessjohann L. A. (2011). The multiple multicomponent approach to natural product mimics: tubugis, N-substituted anticancer peptides with picomolar activity. J. Am. Chem. Soc. 133, 7692–7695. 10.1021/ja2022027 21528905

[B35] Parker J. S. McCormick M. Anderson D. W. Maltman B. A. Gingipalli L. Toader D. (2017). The development and scale-up of an antibody drug conjugate tubulysin payload. Org. Process Res. Dev. 21, 1602–1609. 10.1021/acs.oprd.7b00232

[B36] Peltier H. M. Mcmahon J. P. Patterson A. W. Ellman J. A. (2006). The total synthesis of tubulysin D. JACS 128, 16018–16019. 10.1021/ja067177z 17165738

[B37] Pillow T. H. Sadowsky J. D. Zhang D. Yu S. F. Del Rosario G. Xu K. (2016). Decoupling stability and release in disulfide bonds with antibody-small molecule conjugates. Chem. Sci. 8, 366–370. 10.1039/C6SC01831A 28451181 PMC5365059

[B38] Rennert R. Neundorf I. Beck-Sickinger A. G. (2009). Synthesis and application of peptides as drug carriers. Methods Mol. Biol. 535, 389–403. 10.1007/978-1-59745-557-2_22 19377983

[B39] Ricardo M. G. Llanes D. Rennert R. Jänicke P. Rivera D. G. Wessjohann L. A. (2024). Improved access to potent anticancer tubulysins and linker-functionalized payloads via an all-on-resin strategy. Chem. Eur. J. 30, e202401943. 10.1002/chem.202401943 38771268

[B40] Ricardo M. G. Marrero J. F. Valdes O. Rivera D. G. Wessjohann L. A. (2018). A peptide backbone stapling strategy enabled by the multicomponent incorporation of amide N-substituents. Chem. - A Eur. J. 25, 769–774. 10.1002/chem.201805318 30412333

[B41] Rivera D. G. Ricardo M. G. Vasco A. V. Wessjohann L. A. Van der Eycken E. V. (2021). On-resin multicomponent protocols for biopolymer assembly and derivatization. Nat. Protoc. 16, 561–578. 10.1038/s41596-020-00445-6 33473197

[B42] Rodrigues T. Bernardes G. J. L. (2016). Antibody-Drug Conjugates: the missing link. Nat. Chem. 8, 1088–1090. 10.1038/nchem.2685 27874863

[B43] Sani M. Lazzari P. Folini M. Spiga M. Zuco V. Cesare M. D. (2017). Synthesis and superpotent anticancer activity of tubulysins carrying non-hydrolysable N-substituents on tubuvaline. Chem-Eur J. 23, 5842–5850. 10.1002/chem.201700874 28300330

[B44] Shankar S. P. Jagodzinska M. Malpezzi L. Paolo L. Manca I. Greig I. R. (2013). Synthesis and structure–activity relationship studies of novel tubulysin U analogues – effect on cytotoxicity of structural variations in the tubuvaline fragment. Org. Biomol. Chem. 11, 2273–2287. 10.1039/C3OB27111K 23411563

[B45] Staben L. R. Koenig S. G. Lehar S. M. Vandlen R. Zhang D. Chuh J. (2016). Targeted drug delivery through the traceless release of tertiary and heteroaryl amines from antibody – drug conjugates. Nat. Chem. 8, 1112–1119. 10.1038/nchem.2635 27874860

[B46] Steinmetz H. Glaser N. Herdtweck E. Sasse F. Reichenbach H. Höfle G. (2013). Isolation, crystal and solution structure determination, and biosynthesis of tubulysins - powerful inhibitors of tubulin polymerization from myxobacteria. Angew. Chem. Int. Ed, 1213–1222. 10.1002/asia.201300051 15372566

[B47] Toniolo C. Crisma M. Formaggio F. Peggion C. Monaco V. Goulard C. (1996). Effect of Nα-acyl chain length on the membrane-modifying properties of synthetic analogs of the lipopeptaibol trichogin GA IV. J. Am. Chem. Soc. 118, 4952–4958. 10.1021/ja954081o

[B48] Wang X. M. Liu Y. W. Wang Q. E. Zhou Z. Si C. M. Wei B. G. (2019). A divergent method to key unit of tubulysin V through one-pot diastereoselective Mannich process of N,O-acetal with ketone. Tetrahedron 75, 260–268. 10.1016/j.tet.2018.11.053

[B49] Wipf P. Takada T. Rishel M. J. (2004). Synthesis of the tubuvaline-tubuphenylalanine (Tuv-Tup) fragment of tubulysin. Org. Lett. 6, 4057–4060. 10.1021/ol048252i 15496098

[B50] Worm D. J. Els-Heindl S. Beck-Sickinger A. G. (2020). Targeting of peptide-binding receptors on cancer cells with peptide-drug conjugates. Pept. Sci. 112, e24171. 10.1002/pep2.24171

[B51] Xu X. Fan M. Qi J. Yao L. (2021). Design, synthesis, and antitumor activity evaluation of pretubulysin analogs. Chem. Biol. Drug Des. 98, 341–351. 10.1111/cbdd.13852 33930251

[B52] Zhang H. Qi L. Cai Y. Gao X. (2024). Gastrin-releasing peptide receptor (GRPR) as a novel biomarker and therapeutic target in prostate cancer. Ann. Med. 56 (1), 2320301. 10.1080/07853890.2024.2320301 38442298 PMC10916925

