# Supplementary material for: Development of bombesin-tubulysin conjugates using multicomponent chemistry to functionalize both the payload and the homing peptide
Source: Front Pharmacol. 2024 Nov 12;15:1408091. doi: 10.3389/fphar.2024.1408091 (PMC11589458; doi:10.3389/fphar.2024.1408091)
Supplement: Supplementary file 1 [file DataSheet2.PDF]

Supporting Information

Development of bombesin-tubulysin conjugates using multicomponent chemistry to functionalize both the payload and the homing peptide

Dayma Llanes,[1#] Robert Rennert,[1#] Paul Jänicke,[1] Ibrahim Morgan,[1] Leslie Reguera,[1,2] Daniel G. Rivera,[1,2,*] Manuel G. Ricardo,[2,3,*] Ludger A. Wessjohann[1,*]

[1]Department of Bioorganic Chemistry, Leibniz Institute of Plant Biochemistry, Weinberg 3, 06120, Halle (Saale), Germany.

[2]Laboratory of Synthetic and Biomolecular Chemistry, Faculty of Chemistry, University of Havana, Zapata y G, 10400, Havana, Cuba.

[3]Present address: Department of Biomolecular Systems, Max Planck Institute of Colloids and Interfaces, Am Mühlenberg 1, 14476, Potsdam, Germany.

# These authors contributed equally to that work.

Corresponding authors *emails: dgr@fq.uh.cu; manuel.garciaricardo@mpikg.mpg.de; wessjohann@ipb-halle.de

Table of contents

Abbreviations 3

Synthesis of building blocks (BBs) 3

Bombesin peptide 15d 24

Synthesis of bombesin-tubugi conjugates 29

In vitro RT-qPCR for receptor expression analysis 32

Since the novel bombesin analogues and peptide-tubugi conjugates described herein have been intended to address and target gastrin-releasing peptide receptor (GRPR)-overexpressing tumor cells, the investigated human cell lines have been selected, based on RNA-Seq data analyzed by using the Genevestigator® gene expression analysis tool (Zurich, Switzerland),(5) to cover a broad range of GRPR-overexpressing to GPCR-lowexpressing cells. The actual GRPR mRNA expression of the used cell lines' batches were proven by conducting a RT-qPCR analysis. For that purpose, the mRNA of standardly cultured, untreated cells was isolated using a quick-RNA miniprep kit purchased from Zymo Research (Freiburg i. Br., Germany) and was checked for its concentration and integrity by using a SpectraDrop™ with SpectraMax iD5 (Molecular Devices, San Jose, CA, USA) and 0.5% (w/v) agarose gel electrophoresis, respectively. RevertAid Reverse Transcription (RT) kit and oligo-dT primer from Thermo Fisher Scientific (Waltham, MA, USA) were used with 0.5 µg of the isolated mRNA to synthesize first strand cDNA that was used for subsequent qPCR analyses. QPCR primers, specific for the target receptor hGRPR and the housekeeping gene hGAPDH (glyceraldehyde 3-phosphate dehydrogenase) were designed by using NCBI's Primer-Blast and purchased from Eurofins Genomics (Ebersberg, Germany). The primer sequences are shown in Table S1. The qPCR analyses were conducted by using the GreenMaster mix from Jena Bioscience (Jena, Germany) and BioRad's CFX96TM Real-Time PCR Detection System (Hercules, CA, USA). The qPCR data were processed according to the ΔΔCt methodology using GAPDH as reference gene. RNA isolation, cDNA synthesis and qPCR were performed in accordance with the manufacturers' guidelines. 32

Table S1. QPCR primers for the relative quantification of the cell lines' hGRPR (human gastrin-releasing peptide receptor) mRNA expression levels. 32

Abbreviations

Alloc, allyloxycarbonyl; Bn, bombesin; Boc, *tert*-butoxycarbonyl; DIPEA, diisopropylethylamine; DCM, dichloromethane; DMF, dimethylformamide; FA, formic acid; FCS, fetal calf serum; Fmoc, fluorenylmethyloxycarbonyl; GRPR, gastrin-releasing peptide receptor; HR-MS, high-resolution mass spectrometry; HT-29, human colon cancer cell line; IR, infrared; MeOH, methanol; Mep, methyl pipecolic acid; NMM, *N*-methyl morpholine NMR, nuclear magnetic resonance; PyBOP, benzotriazol-1-yl-oxytripyrrolidinophosphonium hexafluorophosphate; PBS, phosphate-buffered saline; PC-3, human prostate cancer cell line, ^{n}Pr, *n*-propyl; RP-HPLC, reserved-phase high-performance liquid chromatography; R*f*, retention factor; RT, room temperature; SPPS, solid phase peptide synthesis; *t*Bu, *tert*-butyl; TFA, trifluoroacetic acid; THF, tetrahydrofuran; TIPS, triisopropylsilane; TLC, thin layer chromatography; Trt, triphenylmethyl, Tup, tubuphenylalanine; Tuv, Tubuvaline, Xan, Xanthene.

Synthesis of building blocks (BBs)

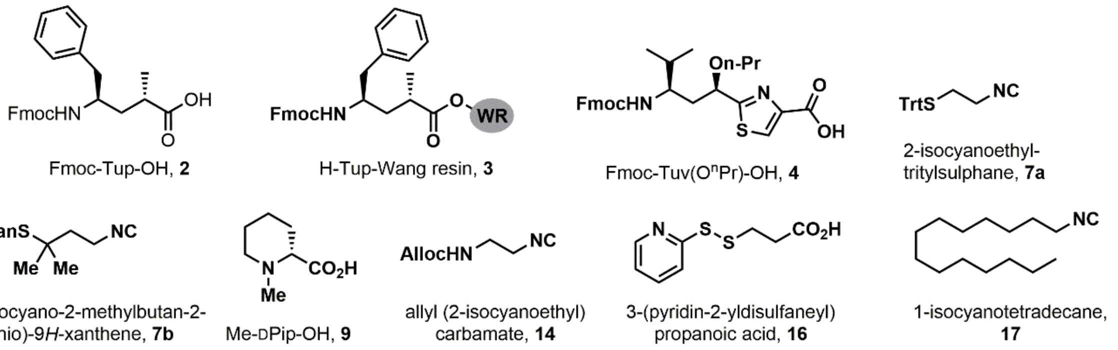

*BBs **2**, **3**, **4**, **7a**, **9**, **14** and **17** were used from our laboratory stock or synthesized according to reported synthetic procedures.*(refs. 1,2) *BBs **7b** and **16** where synthesized as follows**:***

9-(4-Isocyano-2-methylbutan-2-yl-thio)-9H-xanthene (7b)

Xanthene thioether formation

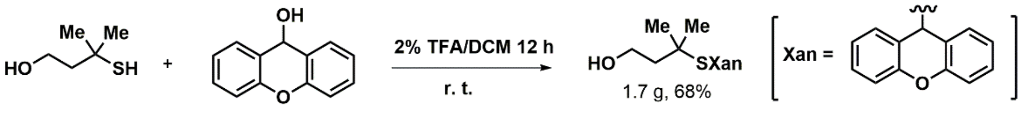

3-mercapto-3-methylbutan-1-ol (1 g, 8.3 mmol) and Xanthidrol (1.8 g, 9.1 mmol, 1.1 eq) were dissolved in 20 mL of DCM at room temperature. Under nitrogen atmosphere, TFA (0.4 mL, resulting in 2% solution) was slowly added and the reaction mixture was stirred overnight. The volatiles were removed under reduced pressure and the crude was purified by column chromatography (n-hex/AcOEt 4:1) to afford the pure 3-((9H-xanthen-9-yl)thio)-3-

methylbutan-1-ol (1.7 g, 68%) as a colorless viscous oil. R*f* (n-hex/AcOEt 4:1) = 0.28. ^{1}H NMR (500 MHz, Chloroform-*d*) δ 7.52 – 7.46 (m, 2H), 7.27 – 7.20 (m, 2H), 7.11 (t, *J* = 7.2 Hz, 4H), 5.37 (s, 1H), 3.60 (t, *J* = 6.9 Hz, 2H), 1.67 (t, *J* = 6.9 Hz, 2H), 1.20 (s, 6H). ^{13}C NMR (126 MHz, $CDCl_3$) δ 152.48, 129.38, 128.45, 123.44, 123.42, 116.76, 59.88, 47.98, 44.72, 41.85, 30.18.

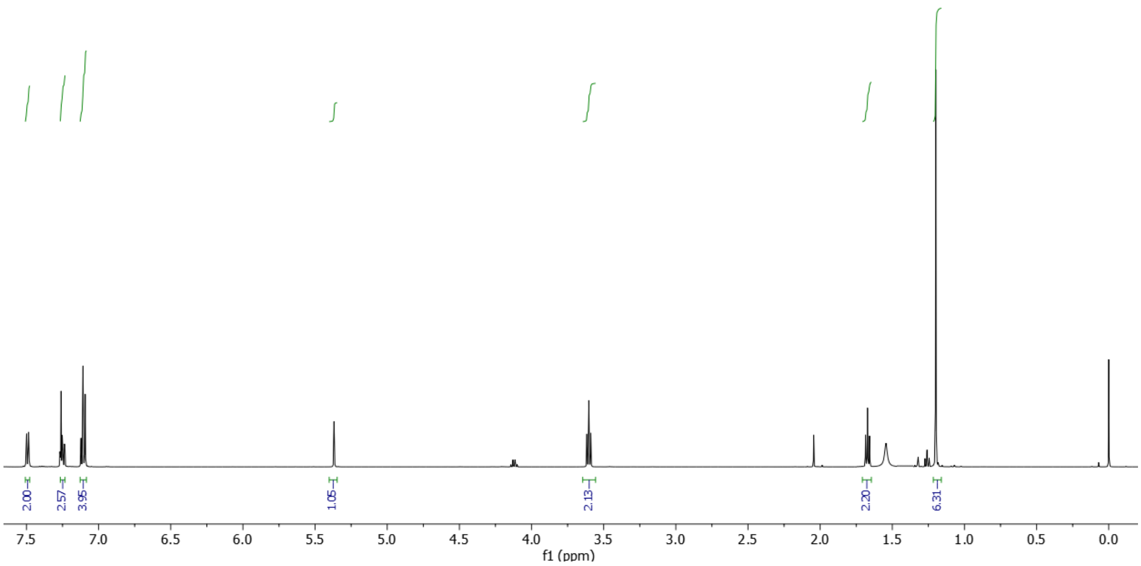

Figure S1. ^{1}H NMR (400 MHz, $CDCl_3$) spectrum of 3-((9H-xanthen-9-yl)thio)-3-methylbutan-1-ol.

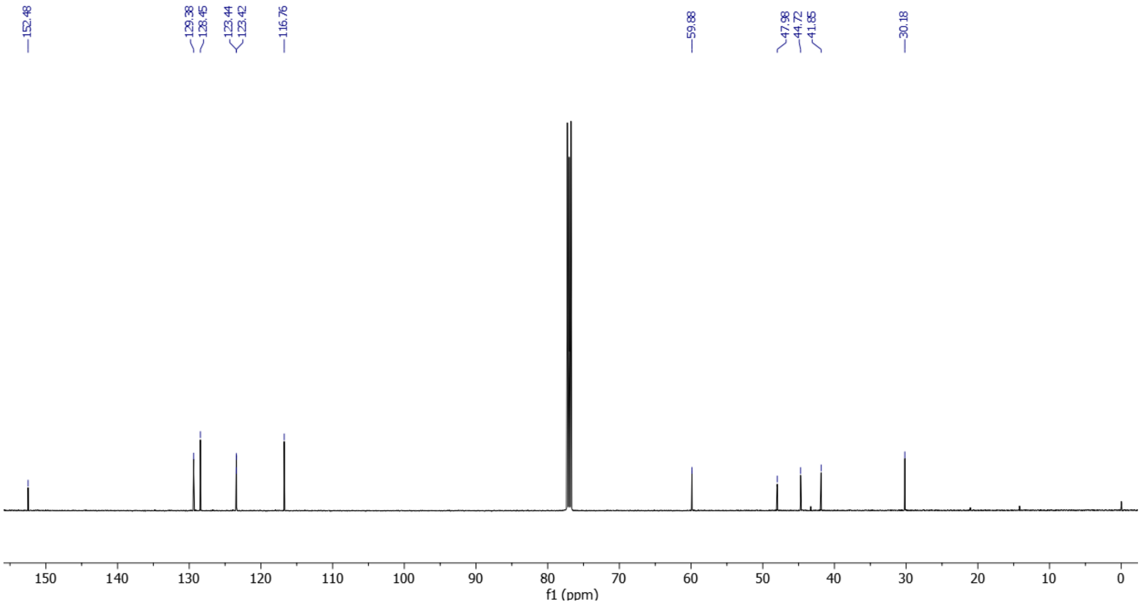

Figure S2. ^{13}C NMR (101 MHz, $CDCl_3$) spectrum of 3-((9H-xanthen-9-yl)thio)-3-methylbutan-1-ol.

Alcohol to azide transformation

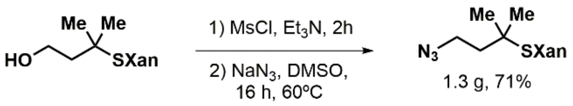

To a mixture of 3-((9H-xanthen-9-yl)thio)-3-methylbutan-1-ol (1.7 g, 5.7 mmol) and Et_3N (1.2 mL, 8.55 mmol) at 0 ºC in DCM (20 mL), MsCl (0.53 mL, 6.8 mmol) was added dropwise for 20 min. The system was allowed to reach room temperature, stirred for an additional 2 h, then diluted with 50 ml of $CHCl_3$ and washed with sat. solution of $NaHCO_3$ (2×20 mL) and brine (2×20 mL). The organic layer was dried over anh. Na_2SO_4 and concentrated under reduced pressure. Without further purification, the crude mesylate was dissolved in dry DMSO (20 mL) and NaN_3 (0.7 g, 11.4 mmol, 2 eq) was added. The mixture was stirred at 60ºC for 16 h, then it was diluted with AcOEt (50 mL), washed with brine (3×20 mL), and dried over anh. Na_2SO_4. After removing all volatiles, the crude was purified by column chromatography (n-hex/DCM 3:1) to afford the pure 9-((4-azido-2-methylbutan-2-yl)thio)-9H-xanthene (1.3 g, 71%) as a colorless viscous oil. R*f* (n-hex/DCM 1:1) = 0.47. ^1H NMR (400 MHz, $CDCl_3$) δ = 7.46 (d, *J* = 7.4 Hz, 2H), 7.30 – 7.21 (m, 2H), 7.11 (t, *J* = 7.5 Hz, 4H), 5.33 (s, 1H), 3.18 – 3.09 (m, 2H), 1.65 – 1.59 (m, 2H), 1.18 (s, 6H). ^{13}C NMR (101 MHz, $CDCl_3$) δ = 152.4, 129.3, 128.6, 123.5, 123.2, 116.8, 47.76, 47.6, 41.9, 40.9, 29.9.

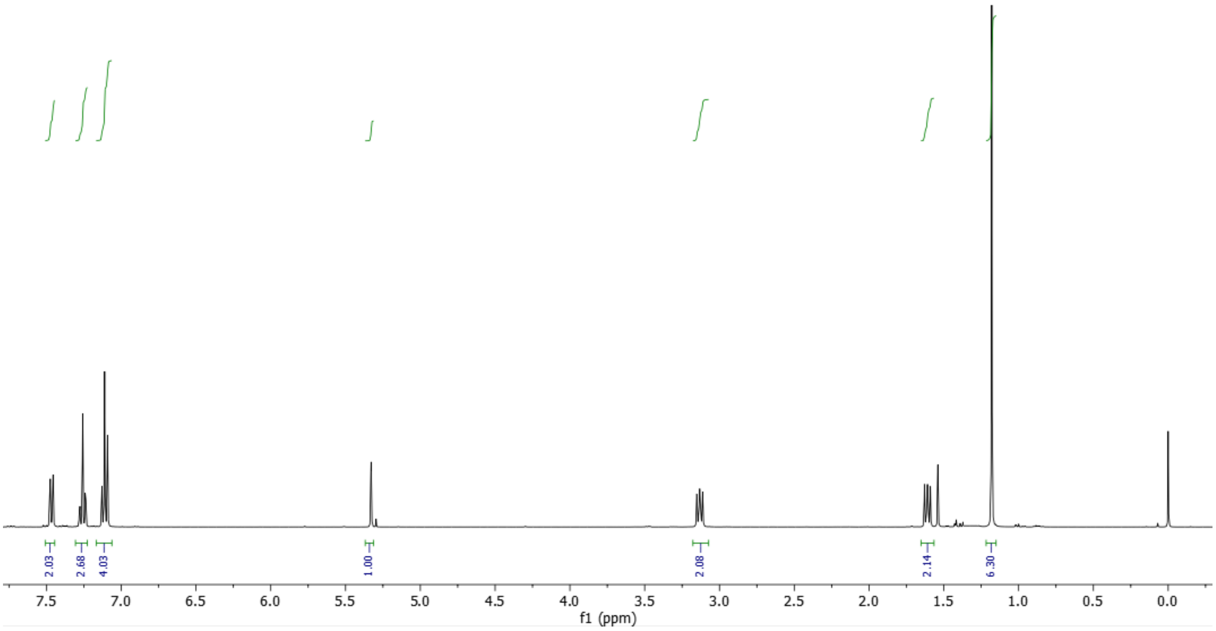

Figure S3. ^1H NMR (400 MHz, $CDCl_3$) spectrum of 9-((4-azido-2-methylbutan-2-yl)thio)-9H-xanthene.

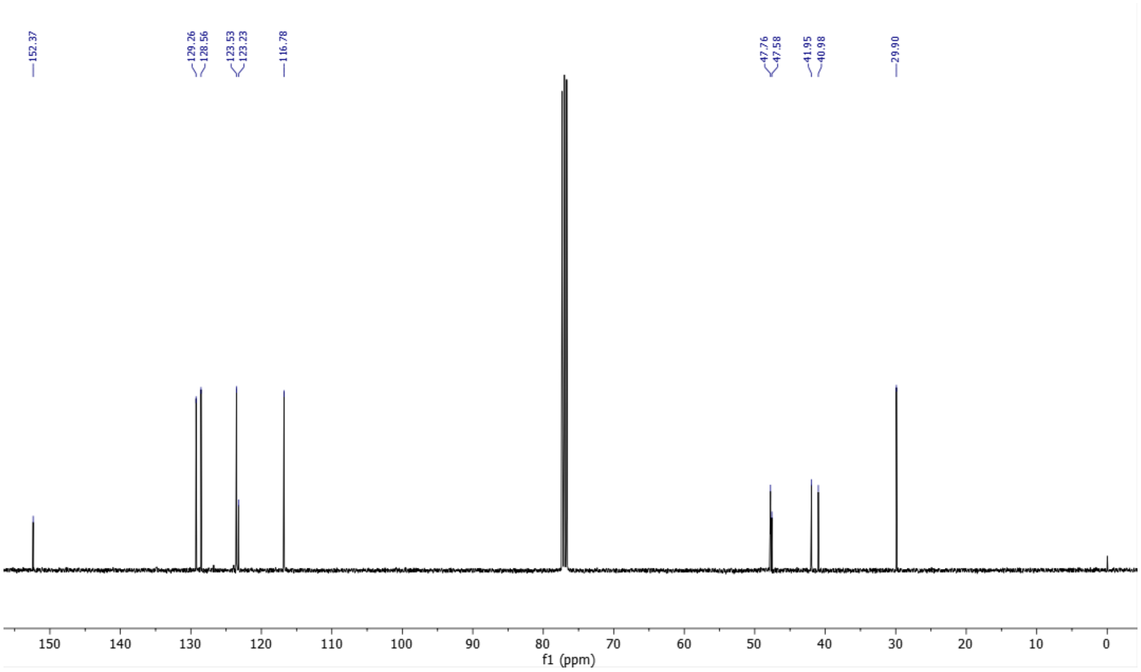

Figure S4. ^{13}C NMR (101 MHz, $CDCl_3$) spectrum of 9-((4-azido-2-methylbutan-2-yl)thio)-9H-xanthene.

Azide reduction and subsequent amine formylation

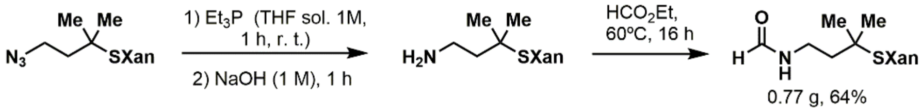

9-((4-azido-2-methylbutan-2-yl)thio)-9H-xanthene (1.2 g, 3.7 mmol) was dissolved in THF (15 mL) and a solution of Et_3P in THF (18 mL, 18 mmol, 1M) was added dropwise for 10 min at 0 °C. The system was allowed to reach room temperature and stirred until completion as indicated by TLC (around 1h). Then, NaOH solution (17 mL, 1M) was added and the system was stirred for an additional hour at room temperature. The reaction mixture was further diluted with water (30 mL) and extracted with chloroform (2×20 mL). The organic phases were combined, washed with brine (2×15 mL), and dried over anh. Na_2SO_4. The solvent was removed under reduced pressure and without further purification, the amine obtained was dissolved in ethyl formate (40 mL) and the solution was refluxed for 12 h. After, the volatiles were removed under reduced pressure, and the crude formamide was purified by column chromatography (DCM/AcOEt 6:1) to afford the pure *N*-(3-((9H-xanthen-9-yl)thio)-3-methylbutyl)formamide (0.77 g 64%) as a colorless viscous oil. R*f* (DCM/AcOEt 5:1) = 0.51. ^1H NMR (400 MHz, $CDCl_3$): δ = 7.97 (d, *J* = 1.7 Hz, 1H), 7.53 – 7.47 (m, 2H), 7.30 – 7.24 (m, 2H), 7.11 (td, *J* = 7.2, 4.1 Hz, 4H), 5.37 (s, 1H), 3.22 – 3.15 (m, 2H), 1.56 – 1.48 (m, 2H), 1.14 (s, 6H). ^{13}C NMR (101 MHz, $CDCl_3$): δ = 164.2, 160.8, 152.4, 129.6, 129.5, 128.7, 128.5,

123.5, 123.5, 123.2, 122.9, 116.8, 116.7, 48.2, 47.65, 43.39, 42.22, 41.9, 41.3, 38.2, 34.63, 30.1, 29.9.

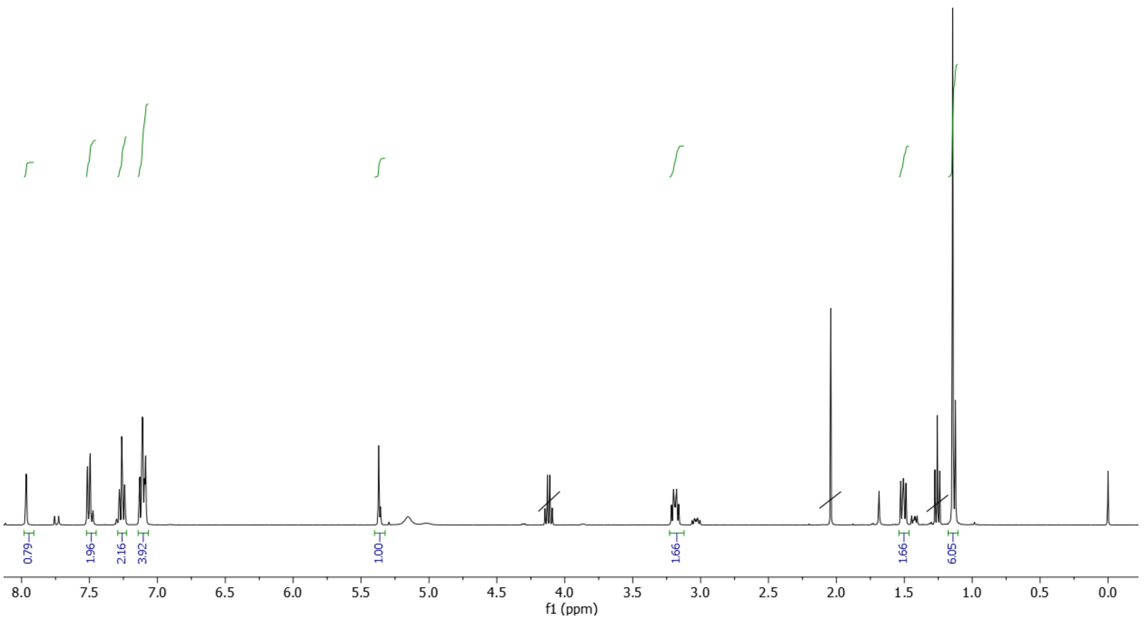

Figure S5. ^{1}H NMR (400 MHz, $CDCl_3$) spectrum of *N*-(3-((9H-xanthen-9-yl)thio)-3-methylbutyl)formamide.

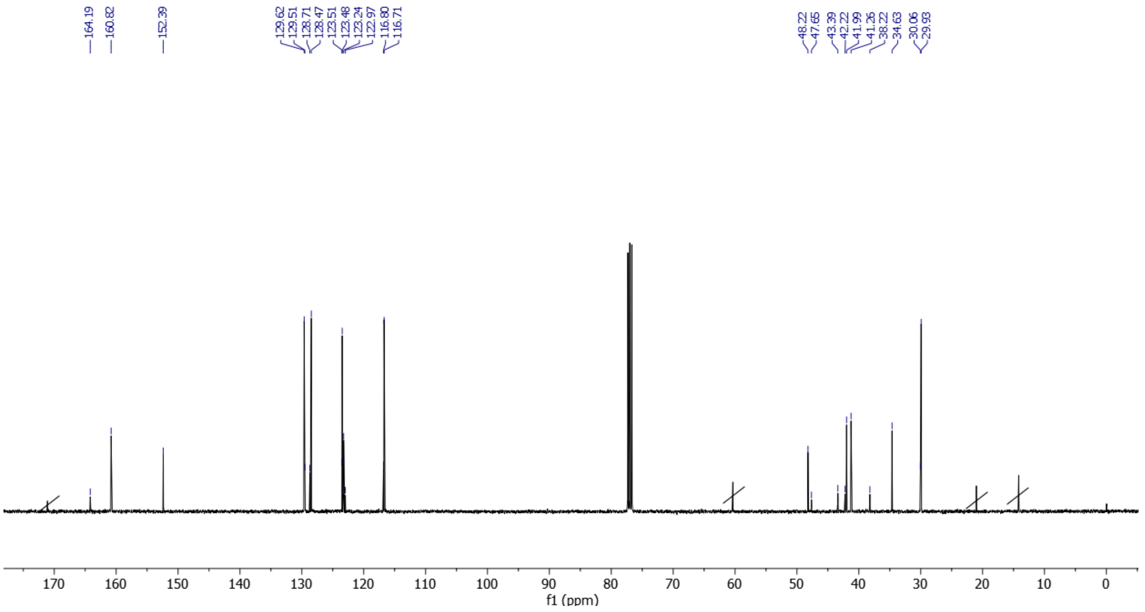

Figure S6. ^{13}C NMR (101 MHz, $CDCl_3$) spectrum of *N*-(3-((9H-xanthen-9-yl)thio)-3-methylbutyl)formamide.

Formamide dehydration

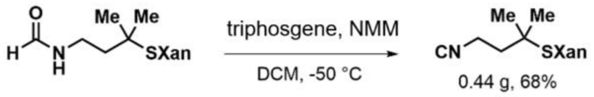

N-(3-((9H-Xanthen-9-yl)thio)-3-methylbutyl)formamide (0.69 g, 2.1 mmol) was dissolved in 20 mL of dry DCM and mixed with NMM (0.61 mL, 5.5 mmol). The solution was cooled at -50 °C and under a nitrogen atmosphere, a solution of triphosgene (0.31 g, 1.05 mmol) in 5 mL of dry DCM was added dropwise for 10 min. The resulting reaction mixture was stirred at -50 °C for 2 h, then allowed to reach room temperature, quenched with 20 mL of Na_2CO_3 (10 %), and extracted with $CHCl_3$ (2×20 mL). The combined organic phases were washed with brine (2×10 mL), dried over anh. Na_2SO_4, and concentrated under reduced pressure to afford after column chromatography (n-hex/DCM 2:1) the pure 9-((4-isocyano-2-methylbutan-2-yl)thio)-9H-xanthene **7b** (0.44 g, 68%) as pale yellow oil. R*f* (n-hex/DCM 2:1) = 0.52. IR (KBr, cm^{-1}): υ_{max} = 3041, 2962, 2917, 2862, 2147, 1658, 1601, 1577. ^{1}H NMR (500 MHz, Chloroform-*d*) δ 7.52 – 7.46 (m, 2H), 7.27 – 7.20 (m, 2H), 7.11 (t, *J* = 7.2 Hz, 4H), 5.37 (s, 1H), 3.60 (t, *J* = 6.9 Hz, 2H), 1.67 (t, *J* = 6.9 Hz, 2H), 1.20 (s, 6H). ^{13}C NMR (101 MHz, $CDCl_3$): δ = 155.9, 129.2, 128.8, 123.7, 122.7, 116.9, 47.6, 42.2, 41.6, 37.9, 29.7.

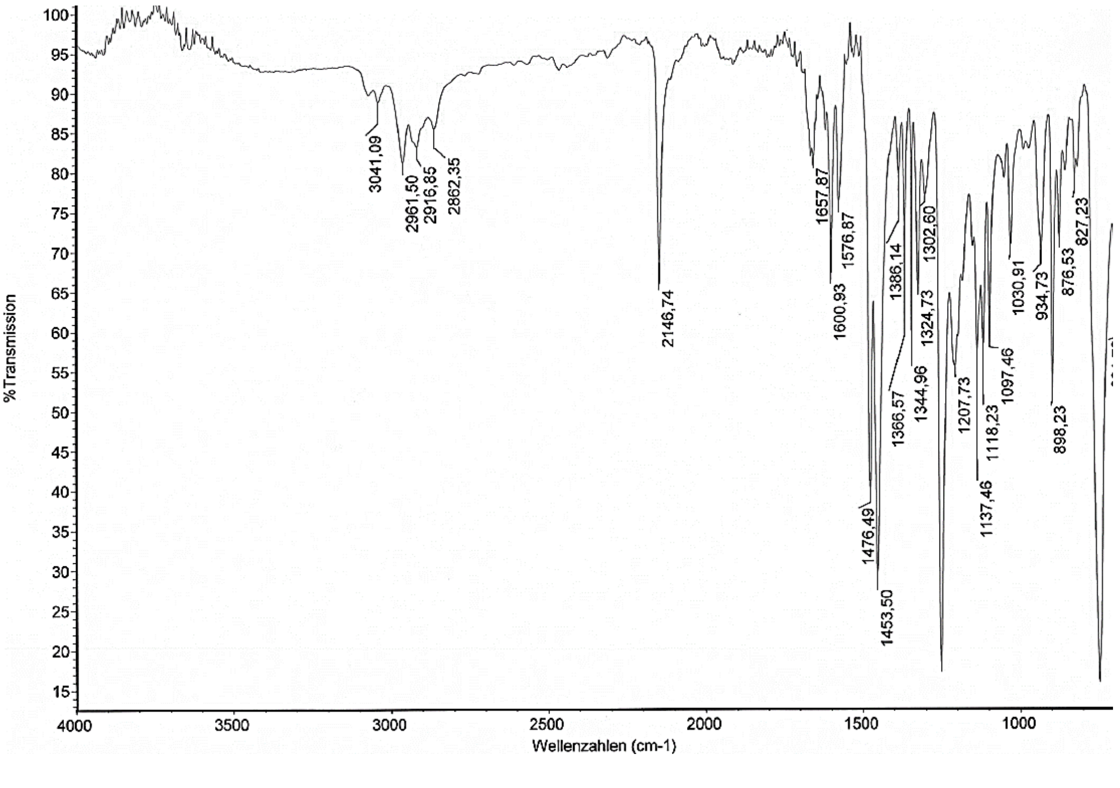

Figure S7. Infrared spectrum of 9-((4-isocyano-2-methylbutan-2-yl)thio)-9H-xanthene **7b**.

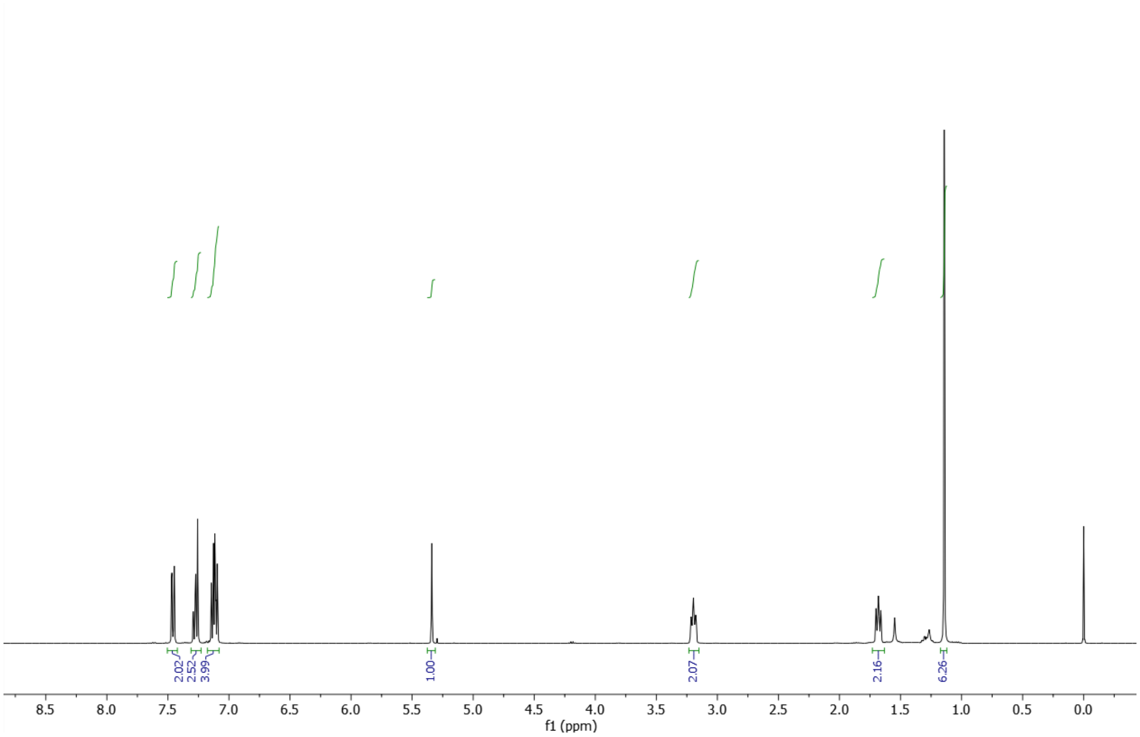

Figure S8. ^{1}H NMR (400 MHz, $CDCl_3$) spectrum of 9-((4-isocyano-2-methylbutan-2-yl)thio)-9H-xanthene.

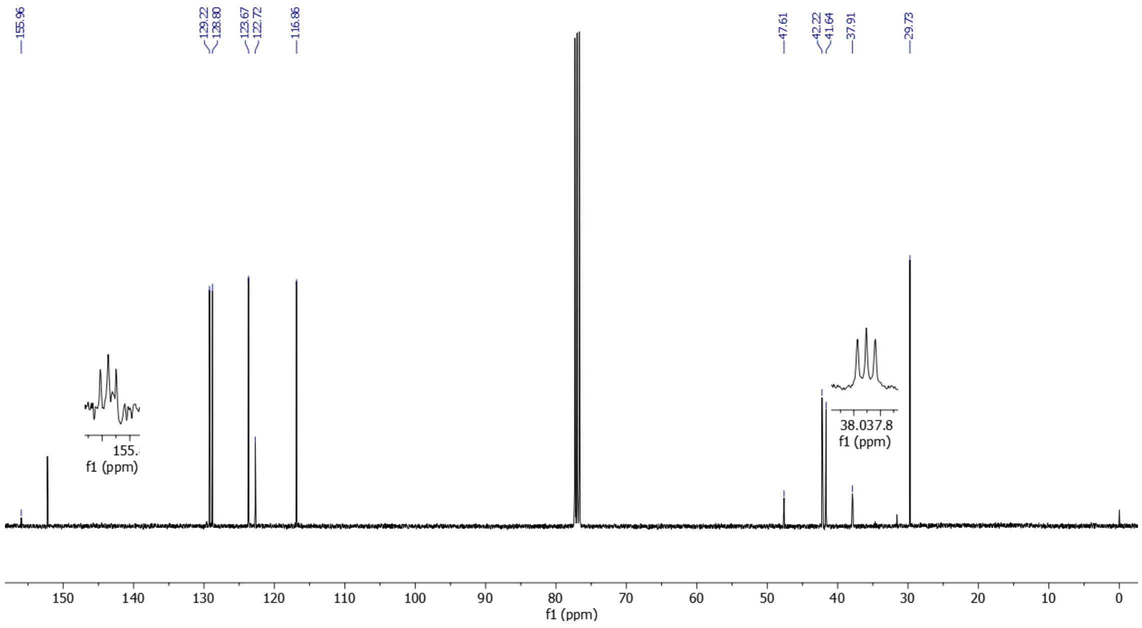

Figure S9. ^{13}C NMR (101 MHz, $CDCl_3$) spectrum of 9-((4-isocyano-2-methylbutan-2-yl)thio)-9H-xanthene.

3-(Pyridin-2-yldisulfaneyl)propionic acid (16)

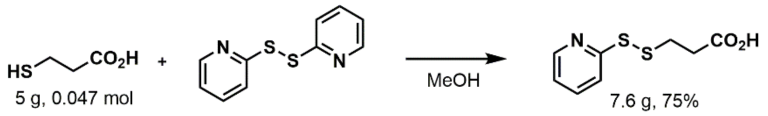

3-Mercaptopropanoic acid (5 g, 0.047 mol) was allowed to react with 1,2-di(pyridin-2-yl)disulfane (20.6 g, 0.094 mol) in MeOH (40 mL), according to the method reported in the literature (ref. 3) to afford after column chromatography (DCM/MeCN 8:1), 3-(pyridin-2-yldisulfaneyl)propanoic acid (7.6 g, 75%) as a white solid. R_f = 0.6 (DCM/MeCN 5:1). ^{1}H NMR (400 MHz, Chloroform-*d*) δ 8.49 (dt, *J* = 4.9, 1.4 Hz, 1H), 7.72 – 7.63 (m, 2H), 7.15 (ddd, *J* = 6.0, 5.0, 2.5 Hz, 1H), 3.07 (t, *J* = 6.8 Hz, 2H), 2.81 (t, *J* = 6.8 Hz, 2H). ^{13}C NMR (101 MHz, cdcl$_3$) δ 175.7, 149.4, 137.4, 121.2, 120.5, 34.1, 33.8.

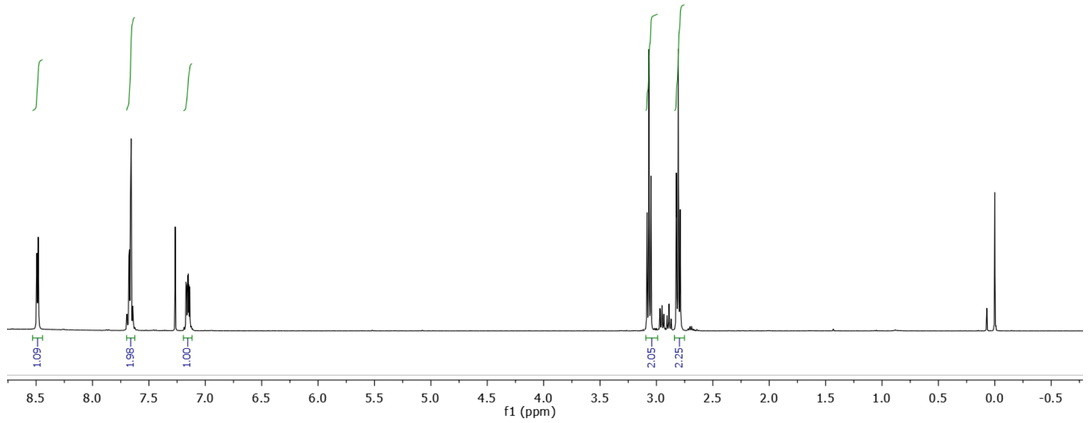

Figure S10. ^{1}H NMR (400 MHz, CDCl$_3$) spectrum of 3-(pyridin-2-yldisulfaneyl)propionic acid.

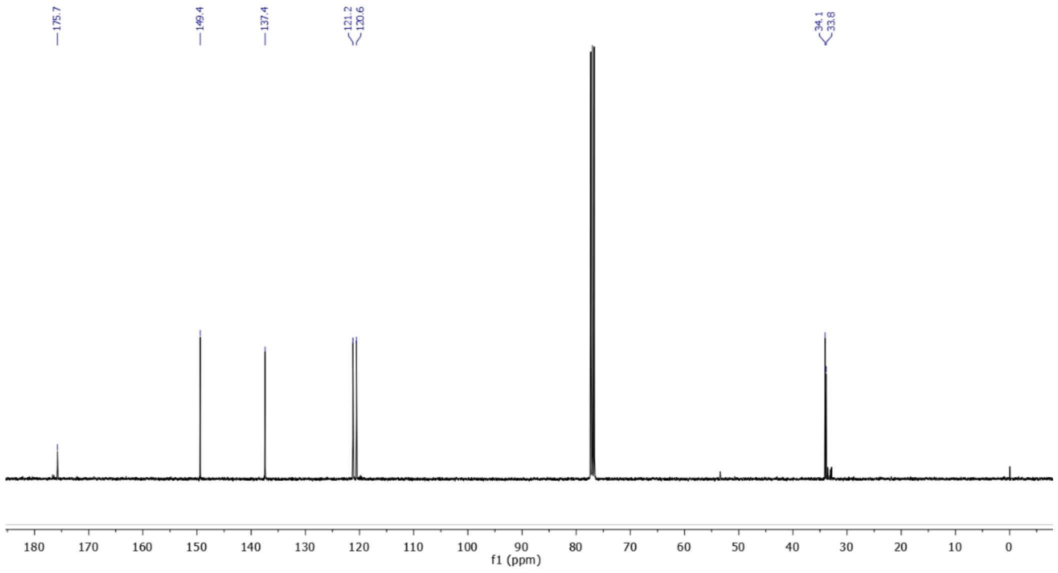

Figure S11. ^{13}C NMR (101 MHz, CDCl$_3$) spectrum of 3-(pyridin-2-yldisulfaneyl)propionic acid.

Tubugi 10a

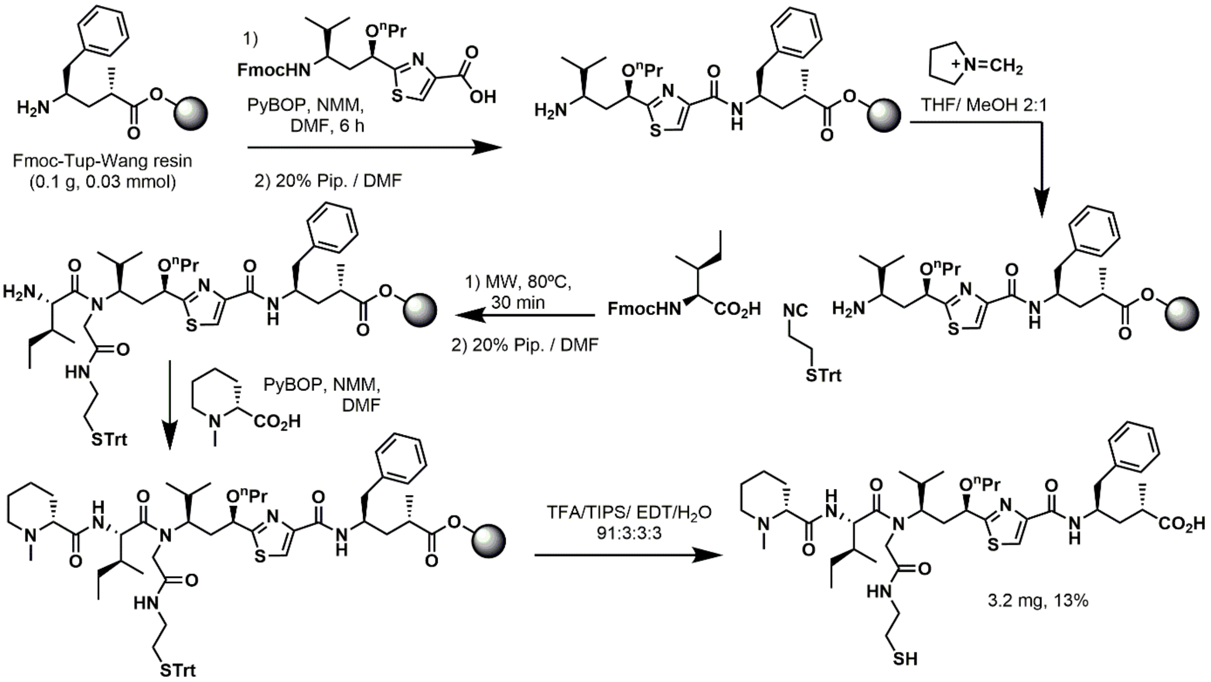

Starting from Fmoc-Tup-Wang resin **3** (100 mg, 0.03 mmol), Fmoc-Tuv(O^nPr)-OH (22.9 mg, 0.045 mmol) was coupled following the general method given above. After removing the Fmoc protecting group, an aminocatalysis-mediated Ugi-reaction was performed using Fmoc-Ile-OH (41 mg, 0.12 mmol), paraformaldehyde (3.6 mg, 0.12 mmol) and 2-(tritylthio)ethylisocyanide (**7a**, 59 mg, 0.18 mmol) as described above. Two reaction cycles were required for the total transformation of the starting material in the Ugi product and also the undesired product of direct coupling at about 10%. Then, the Fmoc protecting group was removed and *N*-Me-D-Pip-OH (17 mg, 0.12 mmol) was coupled using PyBOP (62 mg, 0.12 mmol) and NMM (26 µL, 0.24 mmol) in DMF for 2 h. The crude peptide was finally cleaved from the resin with TFA/TIPS/EDT/H_2O 91:3:3:3 and purified by preparative RP-HPLC to afford the pure Tubugi **10a** (3.2 mg, 13%) as a white solid. R_t = 12.6, 13.3 min. HR-MS *m/z*: 831.4546 $[M+H]^+$, calcd. for $C_{42}H_{67}N_6O_7S_2$: 831.4512.

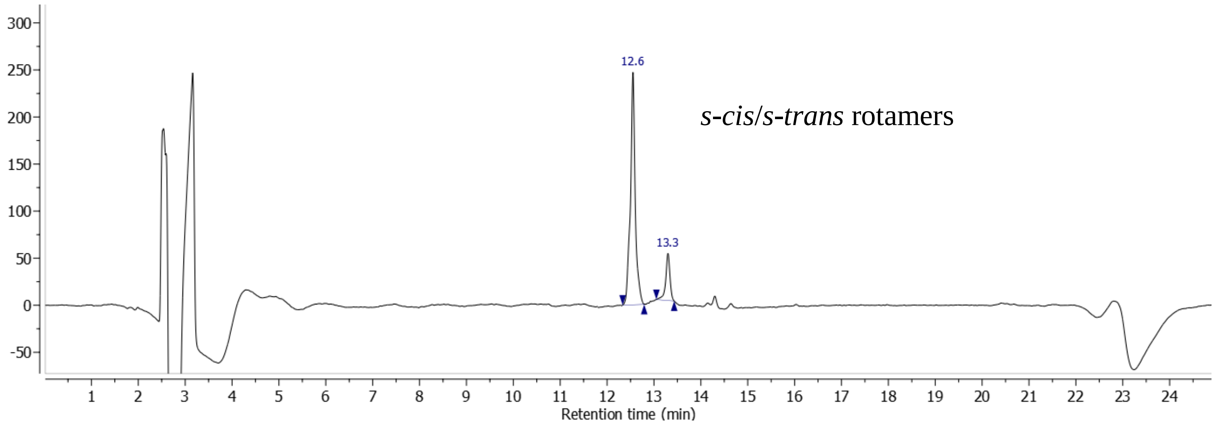

Figure S12. RP-HPLC trace of crude Tubugi **10a**.

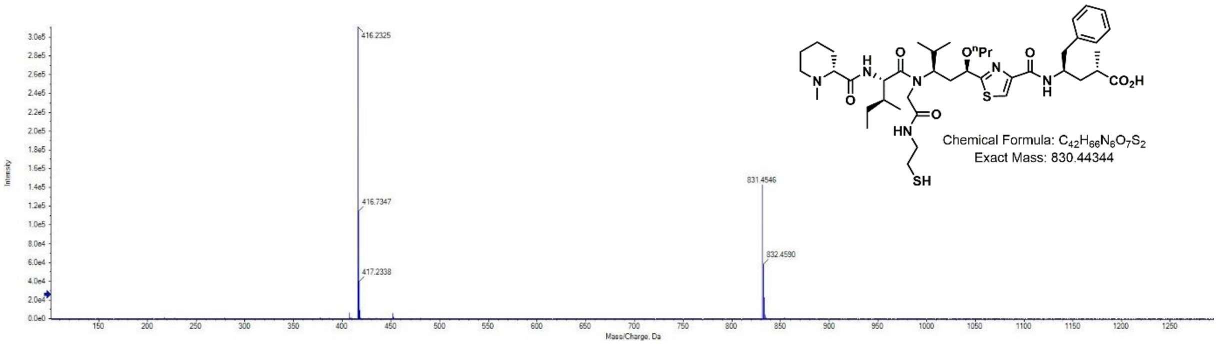

Figure S13. ESI-HRMS of Tubugi **10a**.

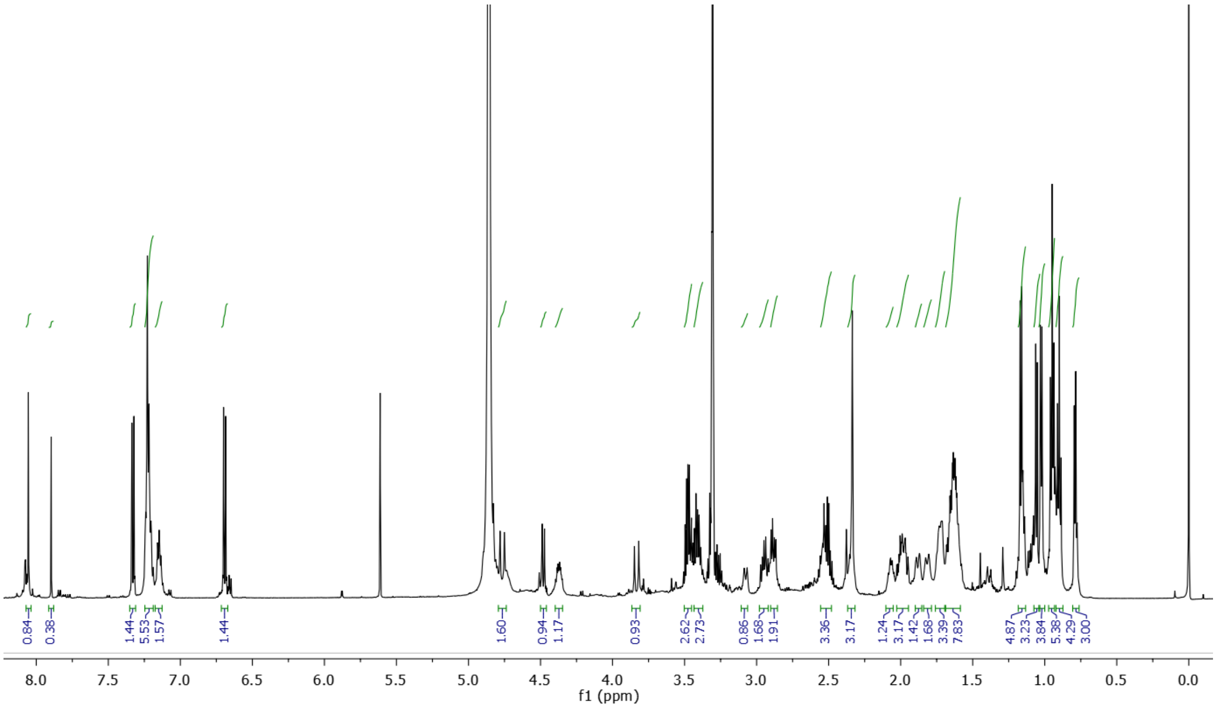

Figure S14. ^{1}H NMR (400 MHz, DMSO-*d*6) spectrum of Tubugi **10a**.

Tubugi 10b

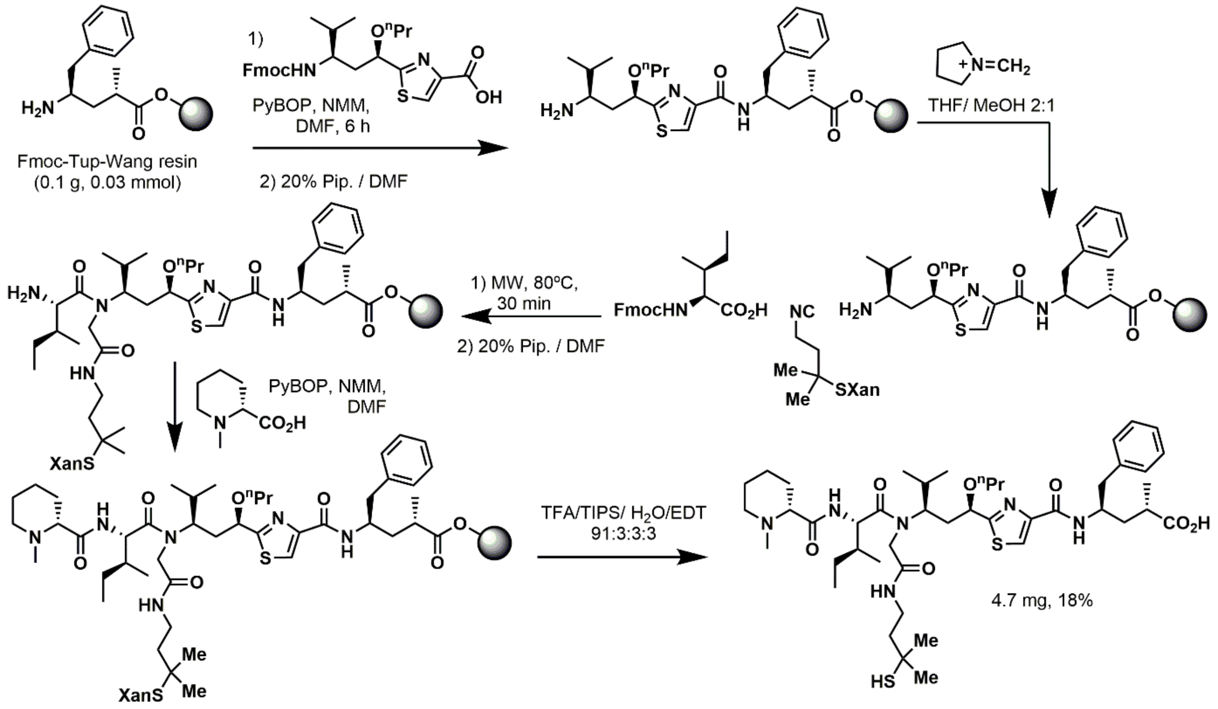

Starting from Fmoc-Tup-Wang resin **3** (100 mg, 0.03 mmol), Fmoc-Tuv(O^{n}Pr)-OH (22.9 mg, 0.045 mmol) was coupled, following the general method given above. After removing the Fmoc protecting group, an aminocatalysis-mediated Ugi-reaction was performed using Fmoc-Ile-OH (41 mg, 0.12 mmol), paraformaldehyde (3.6 mg, 0.12 mmol) and 9-((4-isocyano-2-methylbutan-2-yl)thio)-9H-xanthene (**7b**, 56 mg, 0.18 mmol) as described above. Two reaction cycles were required for the total transformation of the starting material into the desired Ugi product, accompanied by some 10% undesired byproduct of direct. Then, the Fmoc protecting group was removed and *N*-Me-D-Pip-OH (17 mg, 0.12 mmol) was coupled using PyBOP (62 mg, 0.12 mmol) and NMM (26 µL, 0.24 mmol) in DMF for 2 h. The crude peptide was finally cleaved from the resin with TFA/TIPS/EDT/H_2O 91:3:3:3 and purified by preparative RP-HPLC to afford the pure Tubugi **10b** (4.7 mg, 18%) as a white solid. HR-MS *m/z*: 873.5013 $[M+H]^+$, calcd. for $C_{45}H_{72}N_6O_7S_2$: 873.4982.

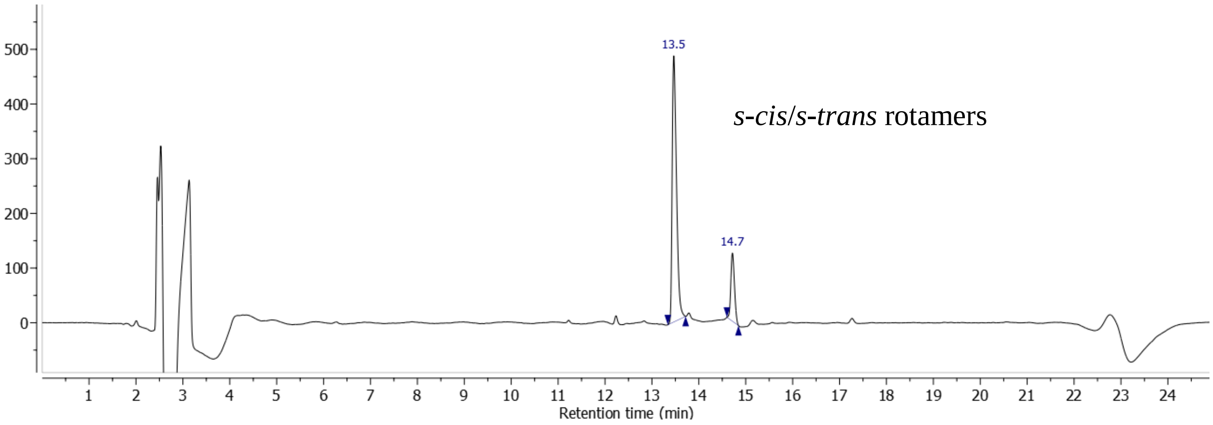

Figure S15. RP-HPLC trace of the pure Tubugi **10b**.

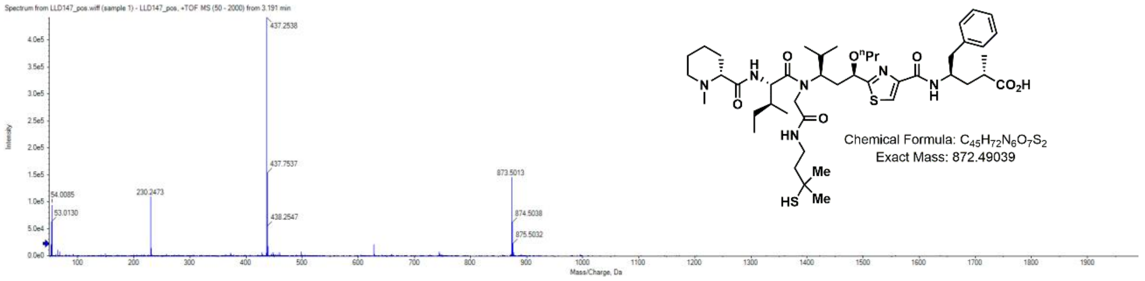

Figure S16. ESI-HRMS of Tubugi **10b**.

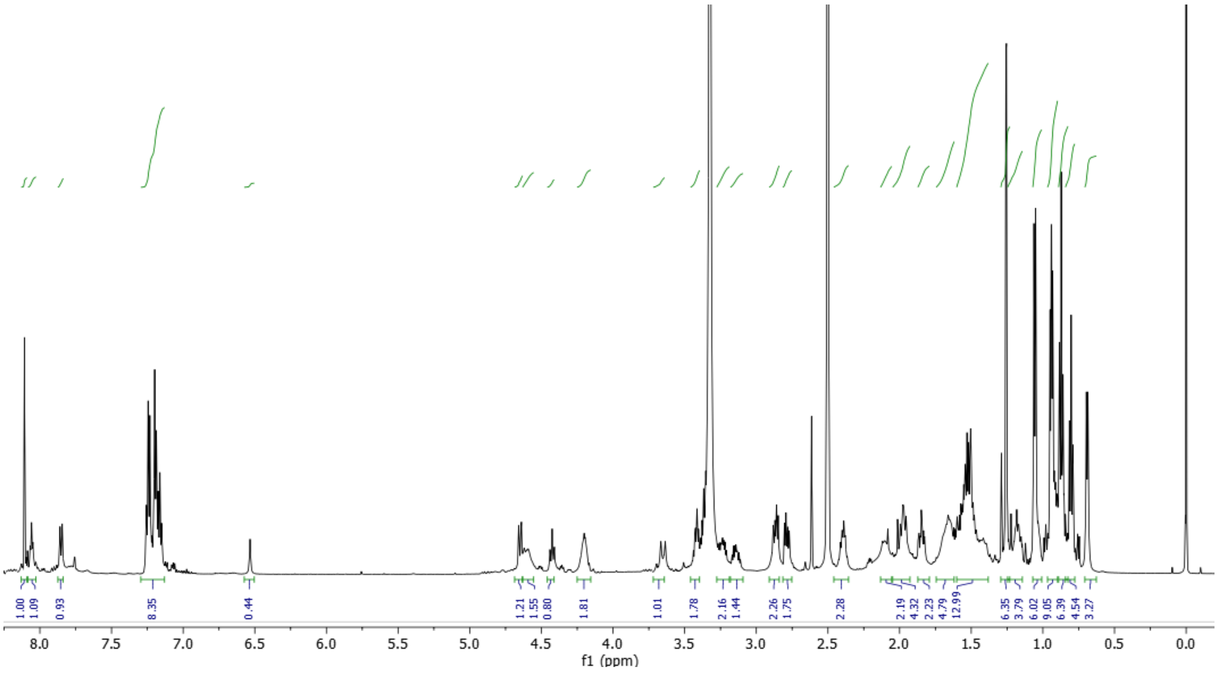

Figure S17. ^{1}H NMR (400 MHz, DMSO-*d*6) spectrum of Tubugi **10b**.

Bombesin peptide 11

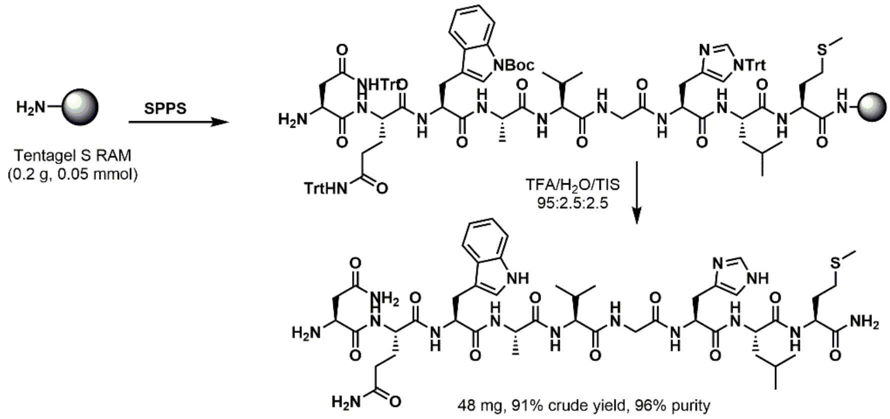

Bombesin peptide (6-14) **11** was synthesized automatically on an INTAVIS ResPepSL peptide synthesizer using TG-S-RAM resin (Iris-Biotech, 0.21 g, 0.24 mmol/g). The peptide was cleaved from the resin with the cocktail TFA/TIPS/H_2O (95:2.5:2.5) to afford the crude peptide **11** (48 mg, 91% crude yield, 96% purity) as a white solid. R_t = 10.5 min. HR-MS m/z: 527.7672 $[M+2H]^{2+}$, calcd. for $C_{47}H_{73}N_{15}O_{11}S$: 527.7667.

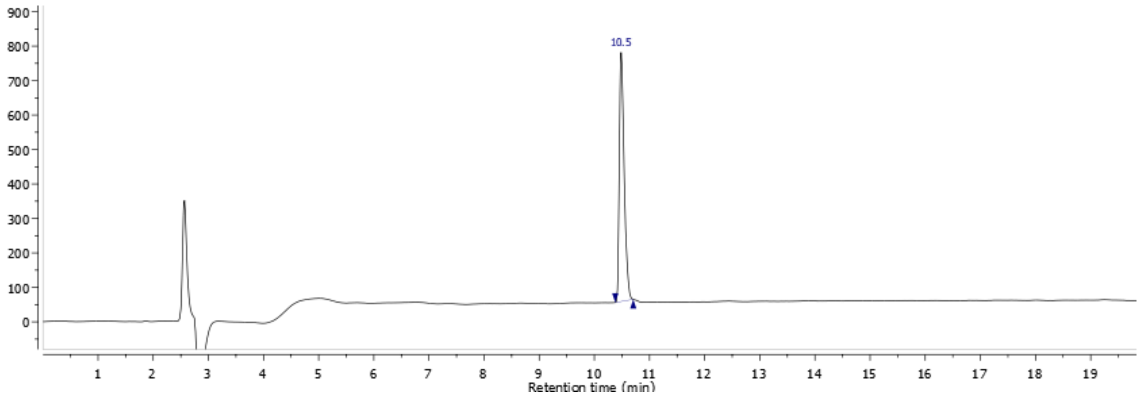

Figure S18. RP-HPLC trace of the pure bombesin peptide **11**.

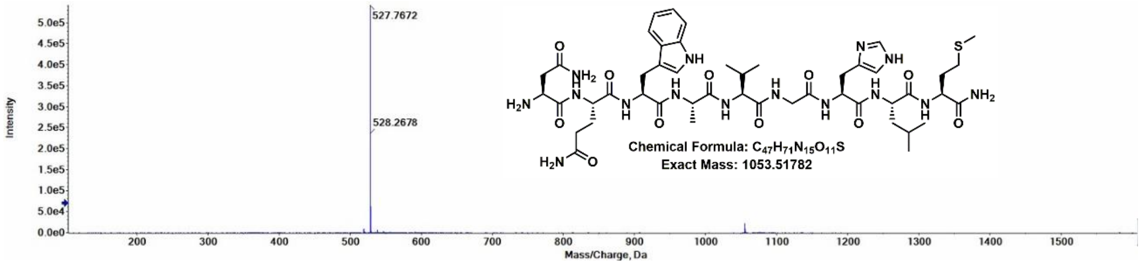

Figure S19. ESI-HRMS of bombesin peptide **11**.

Bombesin peptide 13

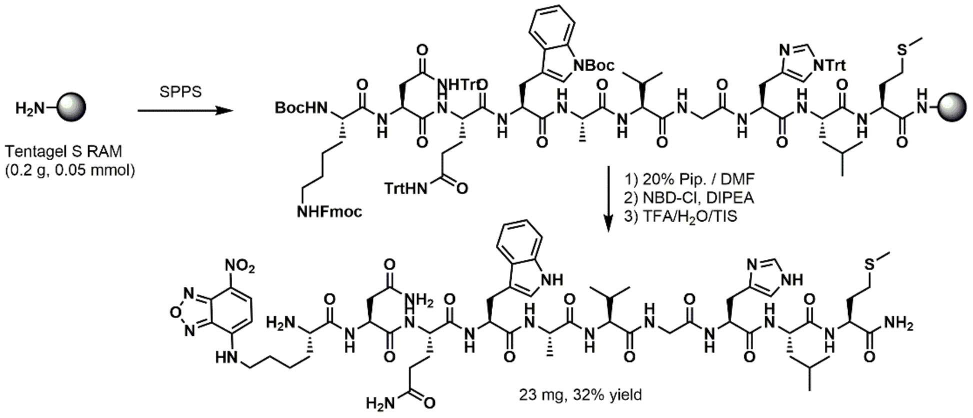

Resin-bound bombesin peptide **12**, was obtained by incorporating manually an additional Boc-Lys(Fmoc)-OH on the bombesin peptide (6-14) **11**, followed by removal of the Fmoc protecting group of the ε-amino group. Then, NBD-Cl was coupled following the protocol given above. The peptide was cleaved from the resin with the cocktail TFA/TIPS/H_2O (95:2.5:2.5) and purified by RP-HPLC to afford the crude peptide **13** (23 mg, 32%) as a white solid. R_t = 11.9 min. HR-MS m/z: 673.3169 $[M+2H]^{2+}$, calcd. for $C_{64}H_{94}N_{20}O_{17}S$: 673.3151.

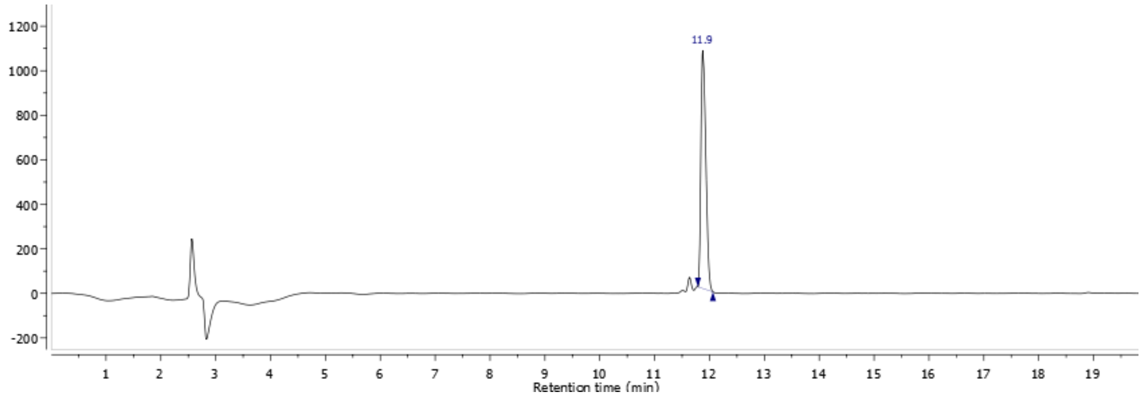

Figure S20. RP-HPLC trace of the pure bombesin peptide **13**.

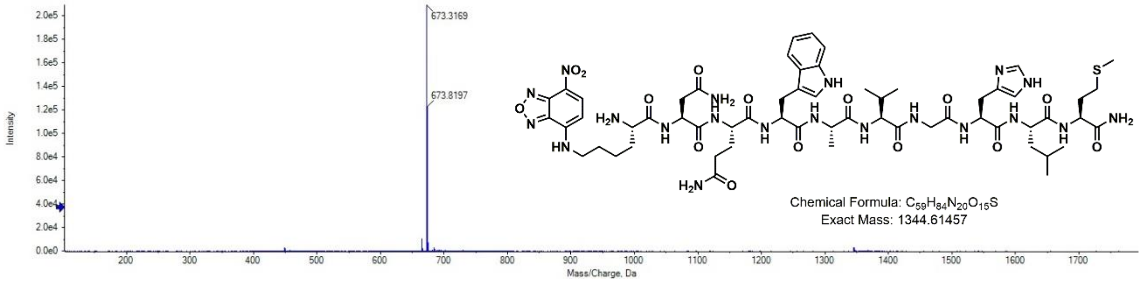

Figure S21. ESI-HRMS of pure bombesin peptide **13**.

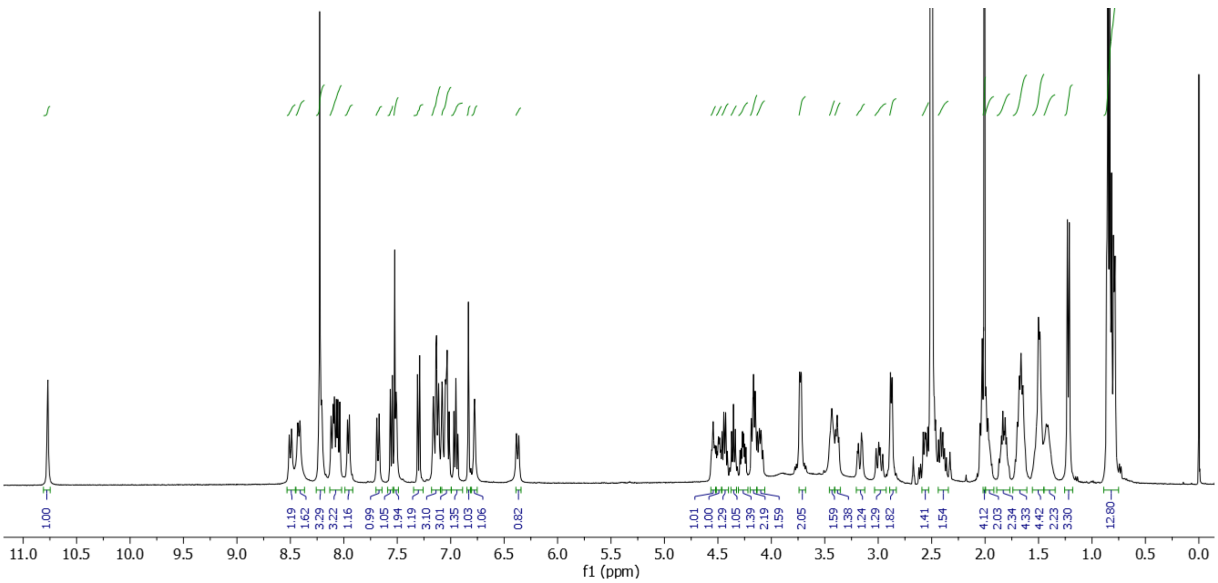

Figure S22. ^{1}H NMR (400 MHz, DMSO-*d*6) spectrum of Bombesin peptide **13**.

Bombesin peptide 15a

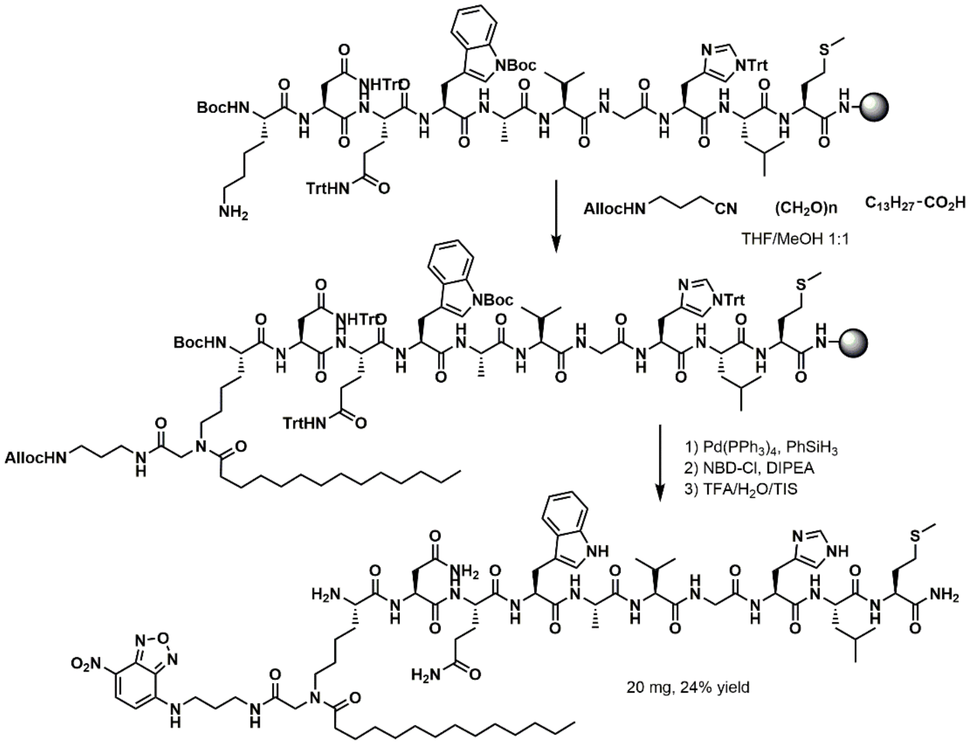

To the truncated resin-bound bombesin peptide was coupled an additional Boc-Lys(Fmoc)-OH, followed by removal of the Fmoc protecting group of the ε-amino group. Then, an aminocatalysis-mediated Ugi-4CR was performed by combining paraformaldehyde (0.6 mg, 0.2 mmol), myristic acid (48 mg, 0.2 mmol), and allyl 3-isocyanopropylcarbamate (51 mg, 0.3 mmol) following the general procedure detailed before. After selective alloc removal, NBD-Cl (24 mg, 0.12 mmol) was incorporated by the method previously described. Finally, the peptide was cleaved from the resin with the cocktail TFA/TIPS/H_2O (95:2.5:2.5) and purified by RP-HPLC to afford the pure peptide **15a** (20 mg, 24% yield) as a white solid. R_t = 12.3 min. HR-MS m/z: 835.4556 $[M+2H]^{2+}$, calcd. for $C_{78}H_{122}N_{22}O_{17}S$: 835.4539.

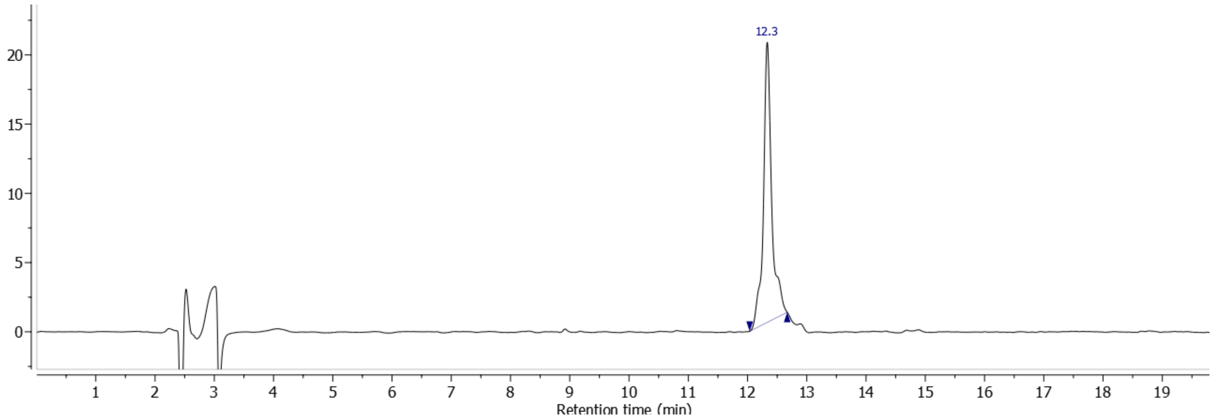

Figure S23. RP-HPLC trace of the pure bombesin peptide **15a**.

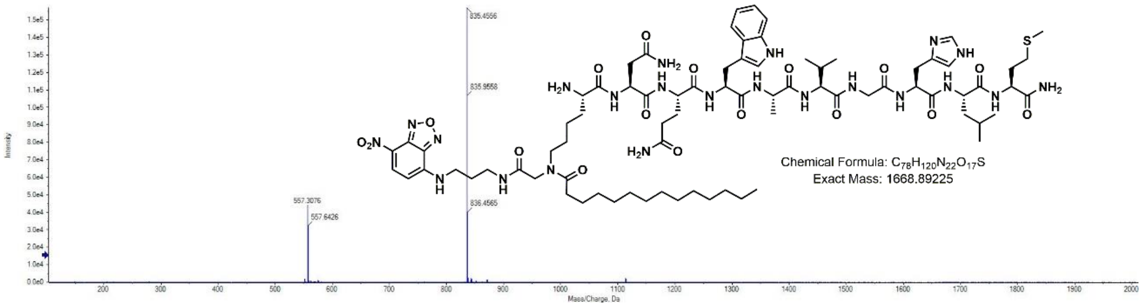

Figure S24. ESI-HRMS of pure bombesin peptide **15a**.

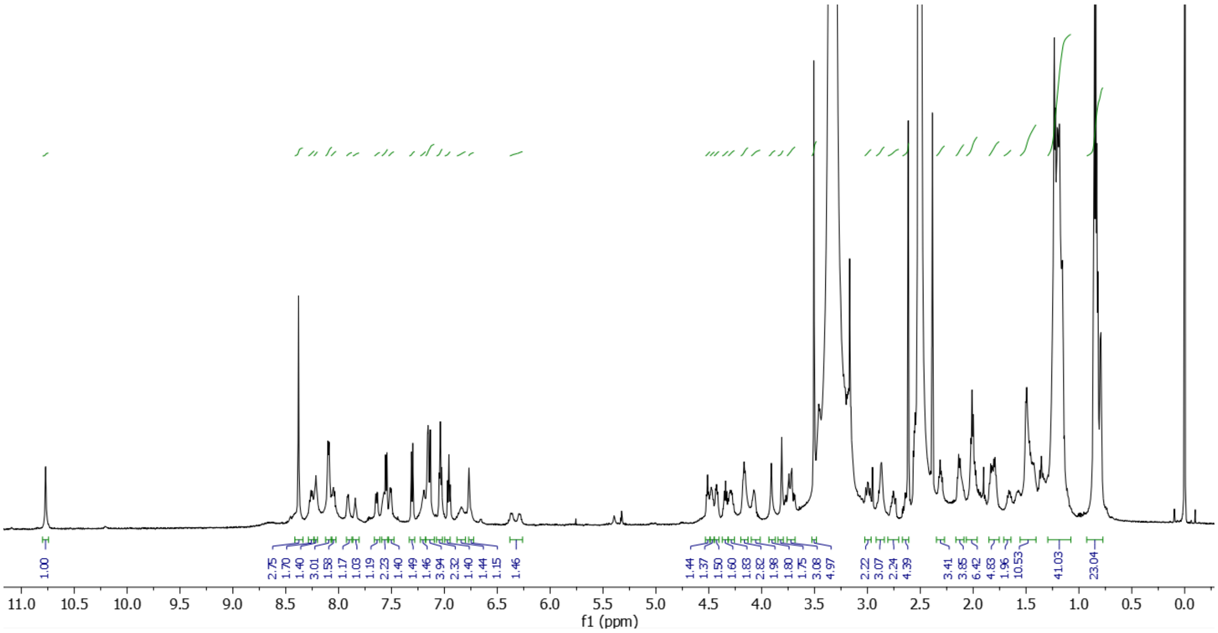

Figure S25. ^{1}H NMR (400 MHz, DMSO-*d*6) spectrum of Bombesin peptide **15a**.

Bombesin peptide 15b

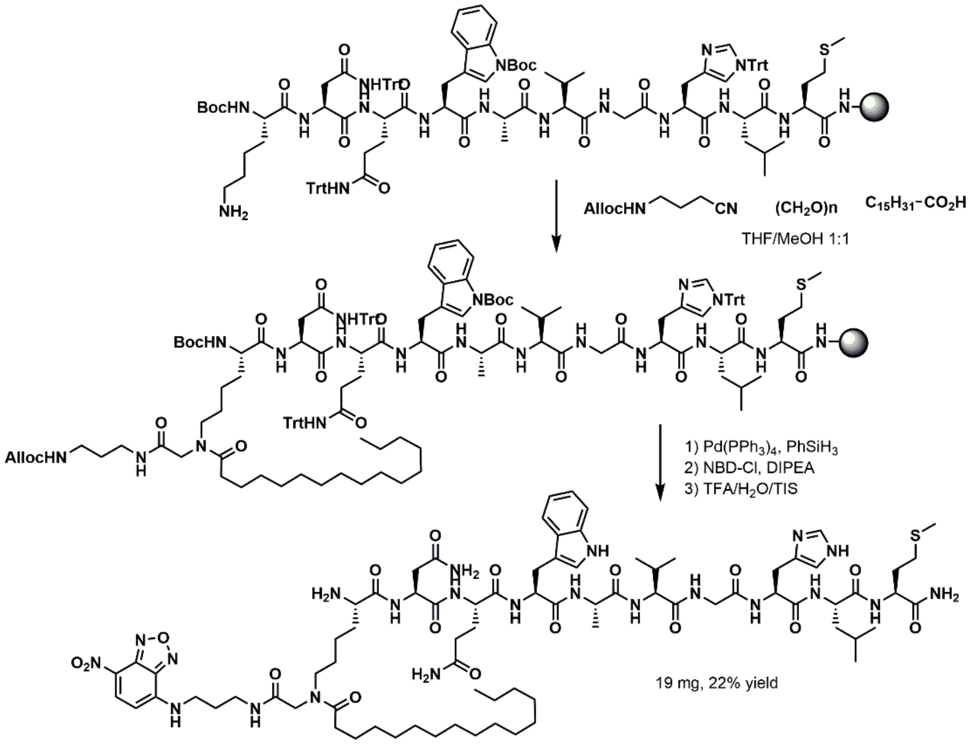

To the truncated resin-bound bombesin peptide was coupled an additional Boc-Lys(Fmoc)-OH, followed by removal of the Fmoc protecting group of the ε-amino group. Then, an aminocatalysis-mediated Ugi-4CR was performed by combining paraformaldehyde (0.6 mg, 0.2 mmol), palmitic acid (51 mg, 0.2 mmol), and allyl 3-isocyanopropylcarbamate (51 mg, 0.3 mmol) following the general procedure detailed before. After selective alloc removal, NBD-Cl (24 mg, 0.12 mmol) was incorporated by the method previously described. Finally, the peptide was cleaved from the resin with the cocktail TFA/TIPS/H_2O (95:2.5:2.5) and purified by RP-HPLC to afford the pure peptide **15b** (19 mg, 22% yield) as a white solid. R_t = 12.6 min. HR-MS m/z: 849.4712 $[M+2H]^{2+}$, calcd. for $C_{80}H_{126}N_{22}O_{17}S$: 849.4696.

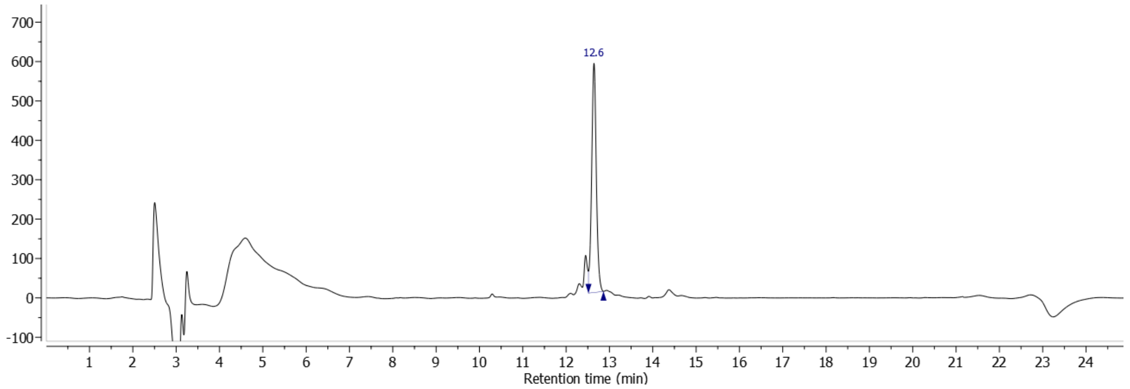

Figure S26. RP-HPLC trace of the pure bombesin peptide **15b**.

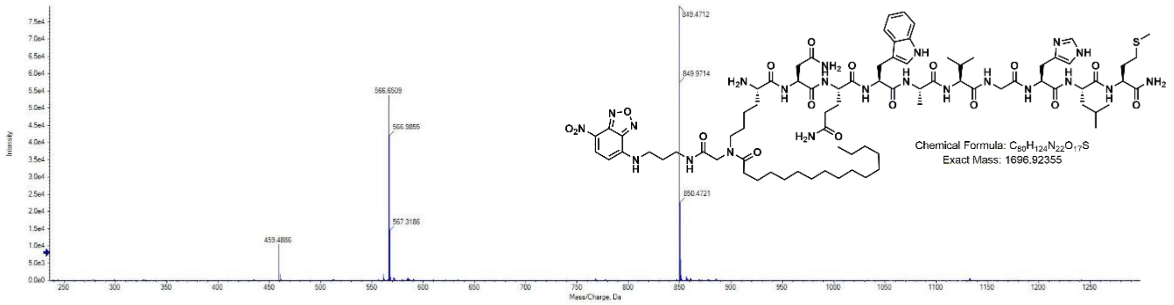

Figure S27. ESI-HRMS of pure bombesin peptide **15b**.

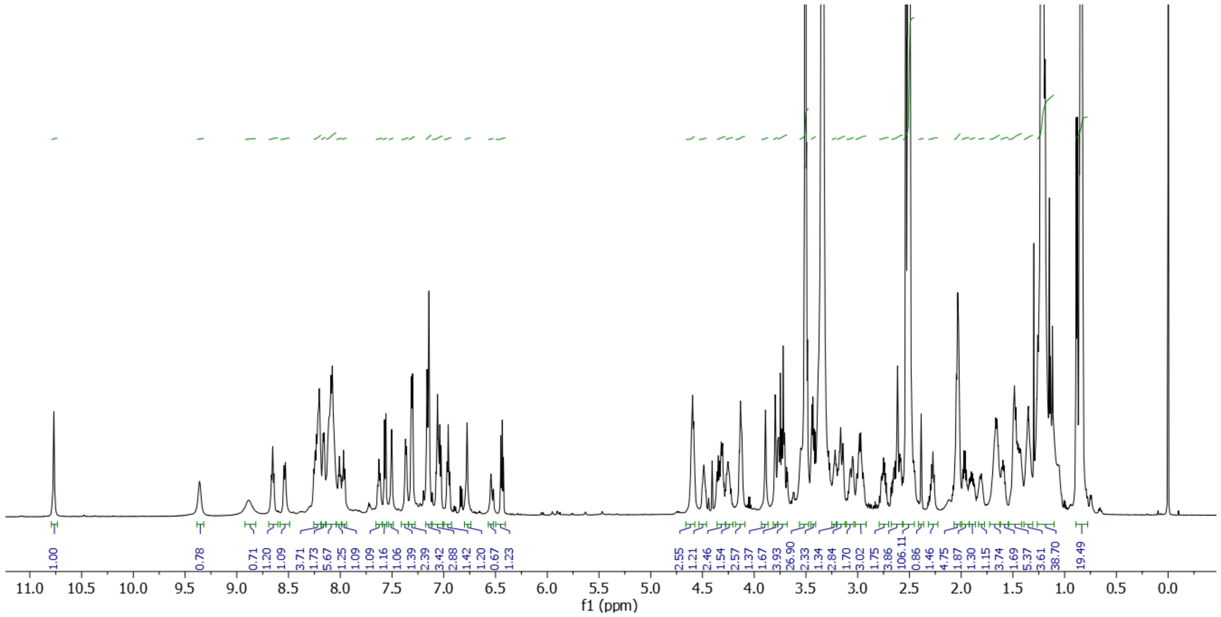

Figure S28. ^{1}H NMR (400 MHz, DMSO-*d*6) spectrum of bombesin peptide **15b**.

Bombesin peptide 15c

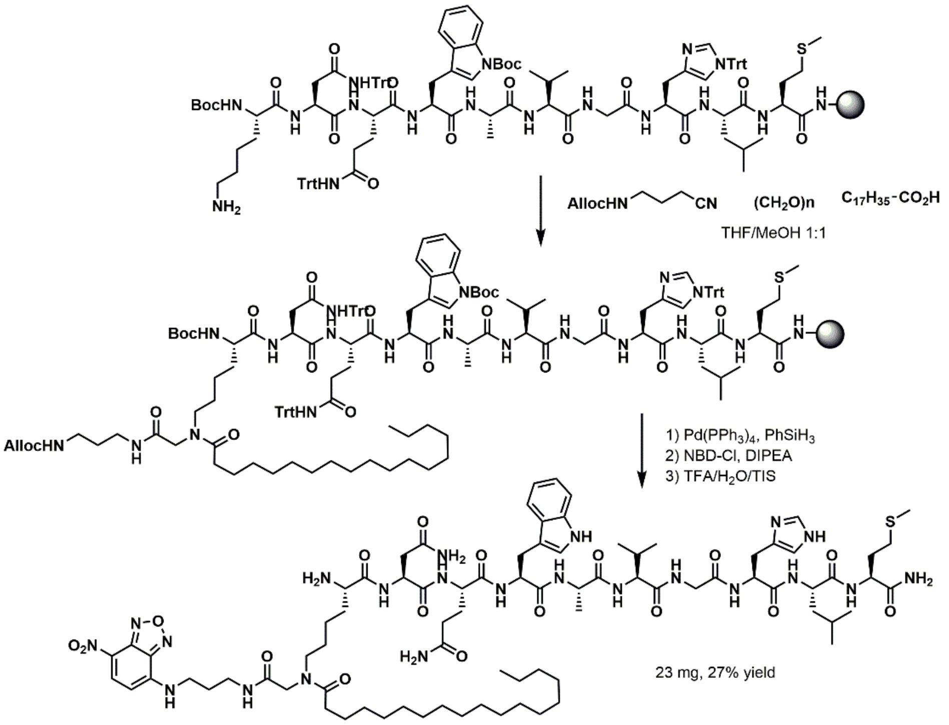

To the truncated resin-bound bombesin peptide was coupled an additional Boc-Lys(Fmoc)-OH, followed by the removal of the Fmoc protecting group of the ε-amino group. Then, an aminocatalysis-mediated Ugi-4CR was performed by combining paraformaldehyde (0.6 mg, 0.2 mmol), oleic acid (56 mg, 0.2 mmol), and allyl 3-isocyanopropylcarbamate (51 mg, 0.3 mmol) following the general procedure detailed before. After selective alloc removal, NBD-Cl (24 mg, 0.12 mmol) was incorporated by the method previously described. Finally, the peptide was cleaved from the resin with the cocktail TFA/TIPS/H_2O (95:2.5:2.5) and purified by RP-HPLC to afford the pure peptide **15c** (23 mg, 27% yield) as a white solid. R_t = 14.6 min. HR-MS m/z: 863.4877 $[M+2H]^{2+}$, calcd. for $C_{82}H_{130}N_{22}O_{17}S$: 863.4853.

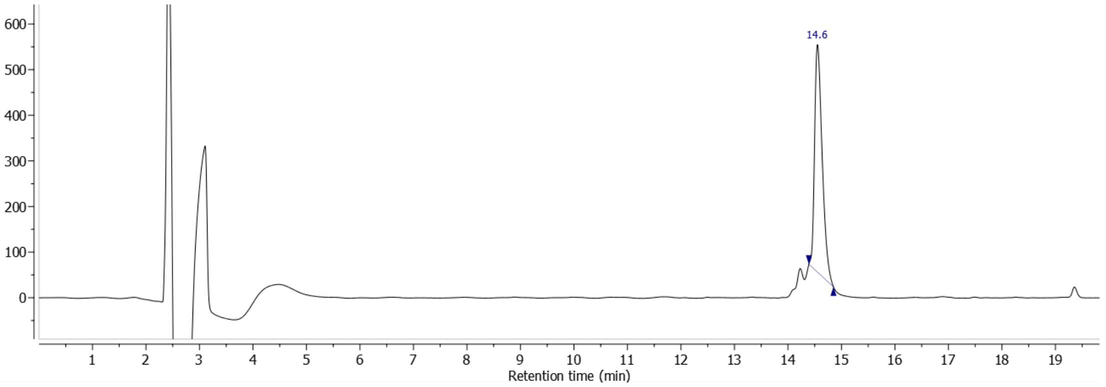

Figure S29. RP-HPLC trace of the pure bombesin peptide **15c**.

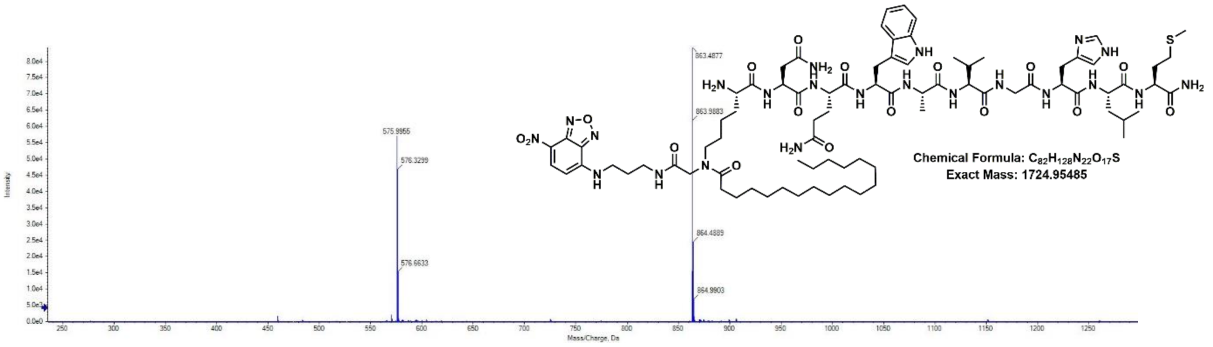

Figure S30. ESI-HRMS of pure bombesin peptide **15c**.

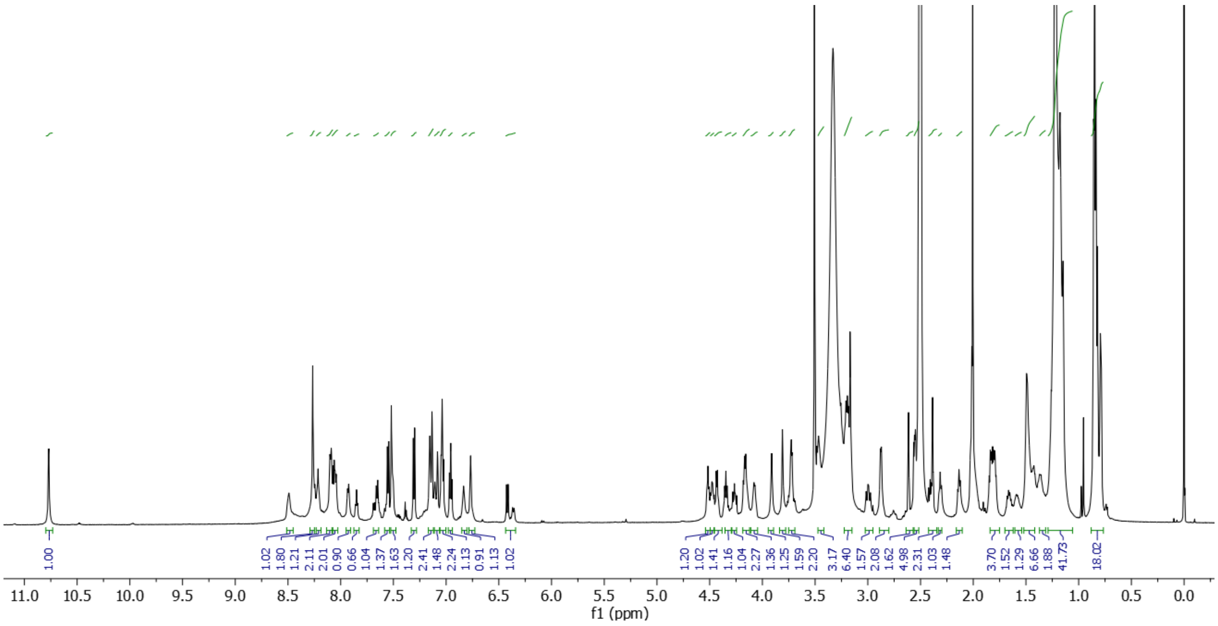

Figure S31. ^{1}H NMR (400 MHz, DMSO-*d*6) spectrum of Bombesin peptide **15c**.

Bombesin peptide 15d

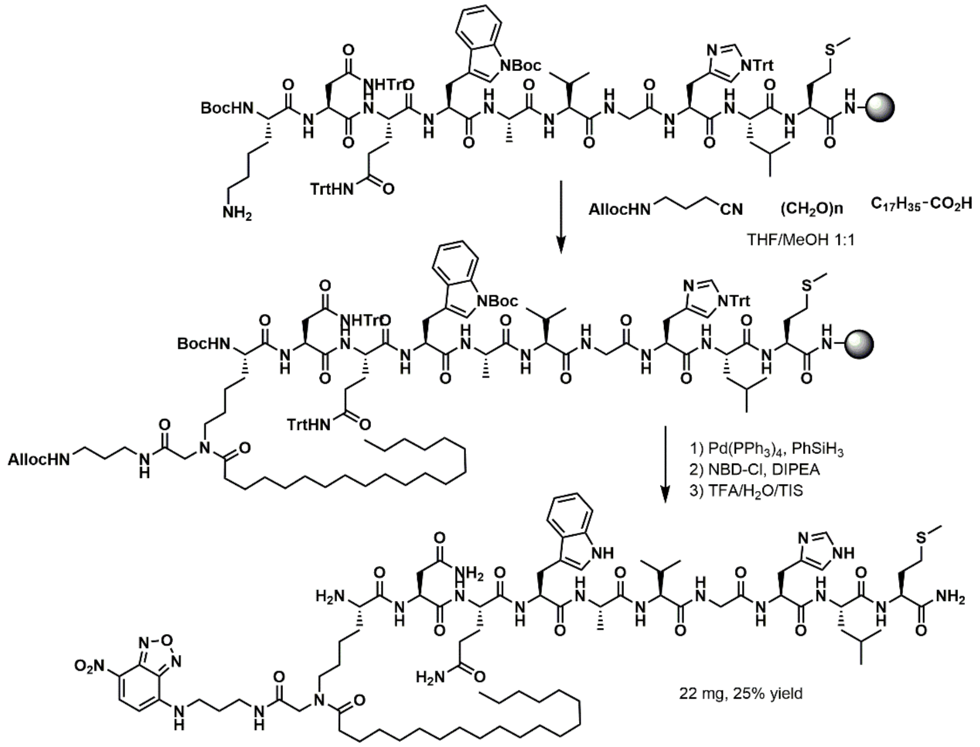

To the truncated resin-bound bombesin peptide was coupled an additional Boc-Lys(Fmoc)-OH, followed by the removal of the Fmoc protecting group of the ε-amino group. Then, an aminocatalysis-mediated Ugi-4CR was performed by combining paraformaldehyde (0.6 mg, 0.2 mmol), arachidic acid (62 mg, 0.2 mmol), and allyl 3-isocyanopropylcarbamate (51 mg, 0.3 mmol) following the general procedure detailed before. After selective alloc removal, NBD-Cl (24 mg, 0.12 mmol) was incorporated by the method previously described. Finally, the peptide was cleaved from the resin with the cocktail TFA/TIPS/H_2O (95:2.5:2.5) and purified by RP-HPLC to afford the pure peptide **15d** (22 mg, 25% yield) as a white solid. R_t = 17.7 min. HR-MS m/z: 877.5044 $[M+2H]^{2+}$, calcd. for $C_{84}H_{134}N_{22}O_{17}S$: 877.5009.

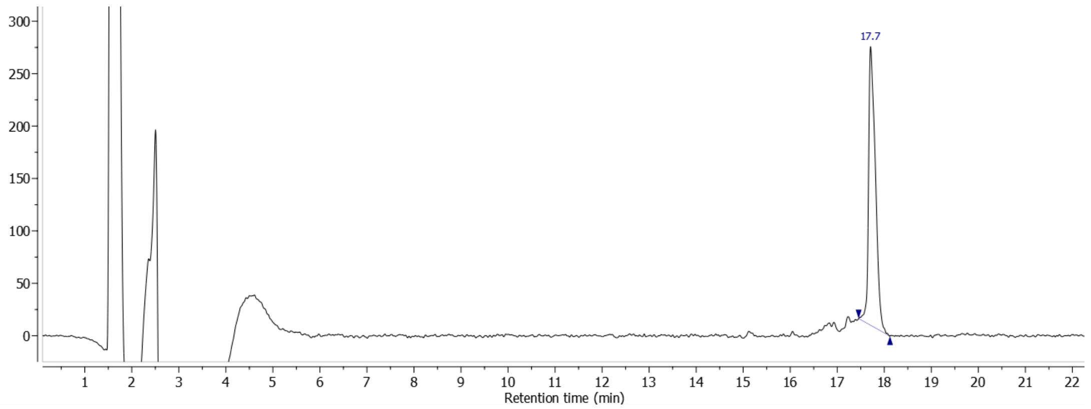

Figure S32. RP-HPLC trace of the pure bombesin peptide **15d**.

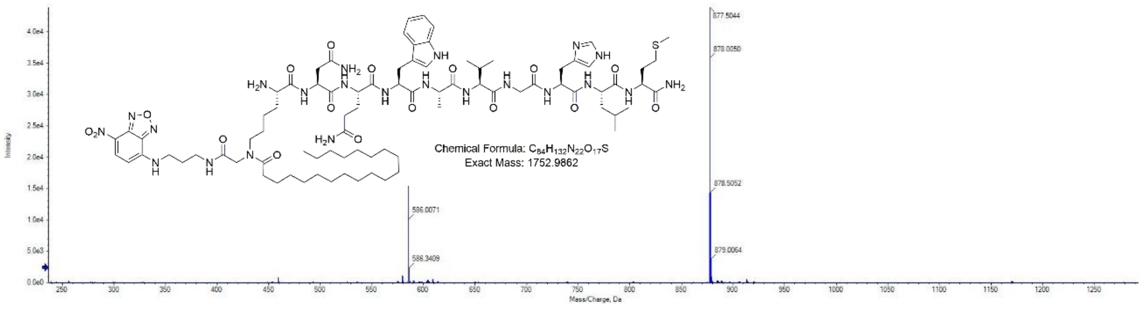

Figure S33. ESI-HRMS of pure bombesin peptide **15d**.

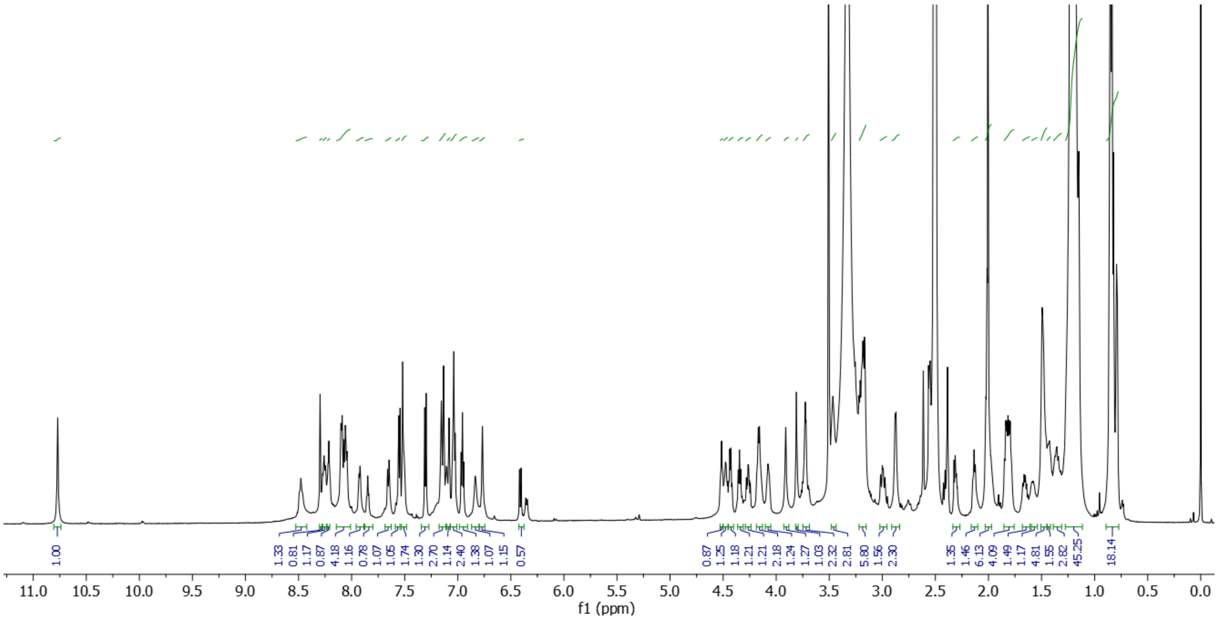

Figure S34. ^{1}H NMR (400 MHz, DMSO-*d*6) spectrum of Bombesin peptide **15d**.

Bombesin peptide 18

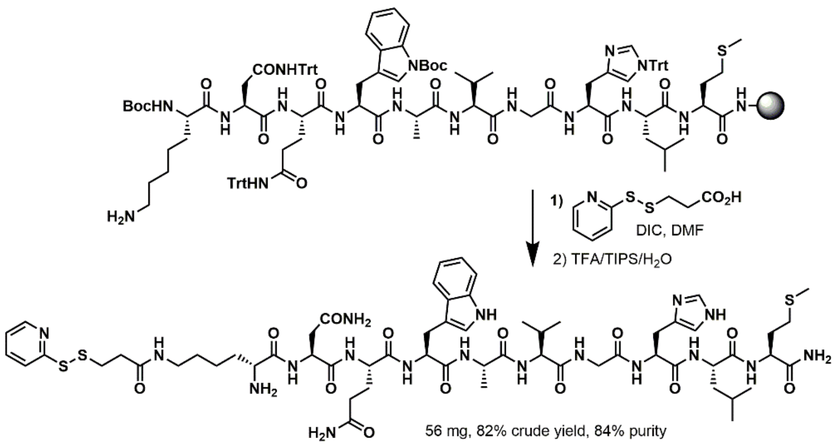

To the truncated resin-bound bombesin peptide was coupled an additional Boc-Lys(Fmoc)-OH, followed by the removal of the Fmoc protecting group from the ε-amino group. Then, 3-(pyridin-2-yldisulfaneyl)propanoic **16** (43 mg, 0.2 mmol) was manually coupled using DIC (31 μL, 0.2 mmol) in DMF for 2 h. The peptide was cleaved from the resin with the cocktail TFA/TIPS/H_2O (95:2.5:2.5) to afford the crude peptide **18** (56 mg, 82% crude yield, 84% purity) as a white solid. R_t = 11.9 min. HR-MS m/z: 690.3215 $[M+2H]^{2+}$, calcd. for $C_{61}H_{92}N_{18}O_{13}S_3$: 690.3127.

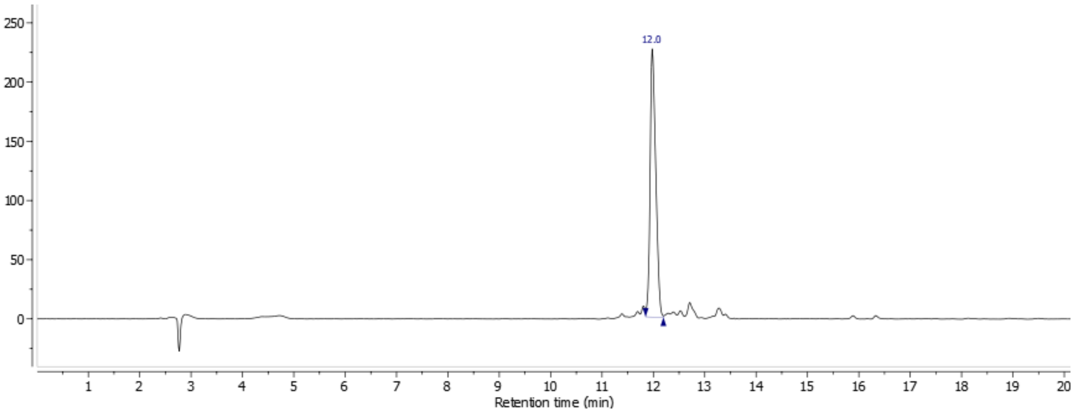

Figure S35. RP-HPLC trace of crude bombesin peptide **18**.

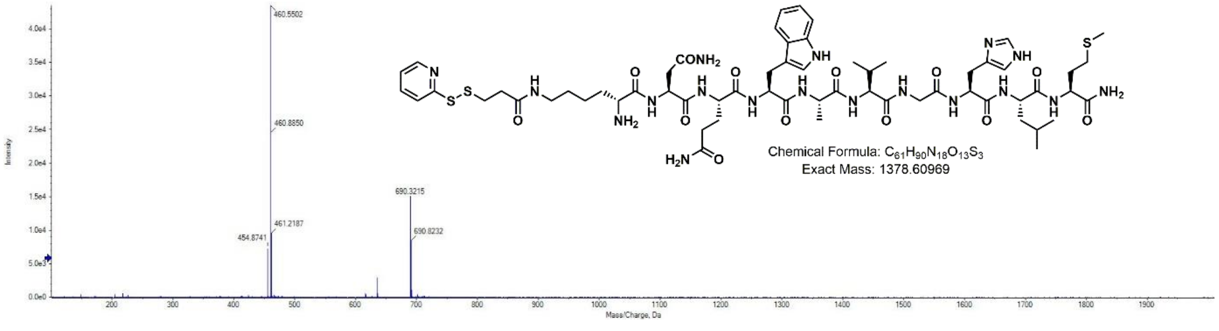

Figure S36. ESI-HRMS of bombesin peptide **18**.

Bombesin peptide 19

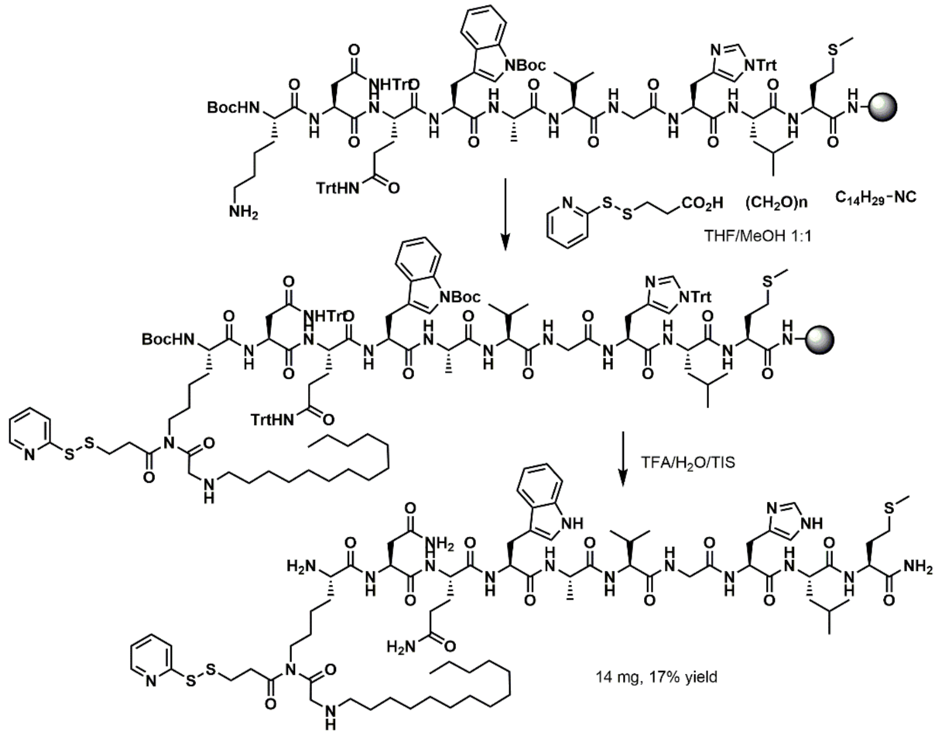

To the truncated resin-bound bombesin peptide was coupled an additional Boc-Lys(Fmoc)-OH, followed by the removal of the Fmoc protecting group from the ε-amino group. Then, an aminocatalysis-mediated Ugi-4CR was performed by combining paraformaldehyde (0.6 mg, 0.2 mmol), 3-(pyridin-2-yldisulfaneyl)propanoic (43 mg, 0.2 mmol) **16** and 1-isocyanotetradecane **17** (67 mg, 0.3 mmol) following the general procedure detailed before. Finally, the peptide was cleaved from the resin with the cocktail TFA/TIPS/H_2O (95:2.5:2.5) and purified by RP-HPLC to afford the pure peptide **19** (20 mg, 24% yield) as a white solid. R_t = 13.0 min. HR-MS m/z: 816.9276 $[M+2H]^{2+}$, calcd. for $C_{77}H_{123}N_{19}O_{14}S_3$: 816.9329.

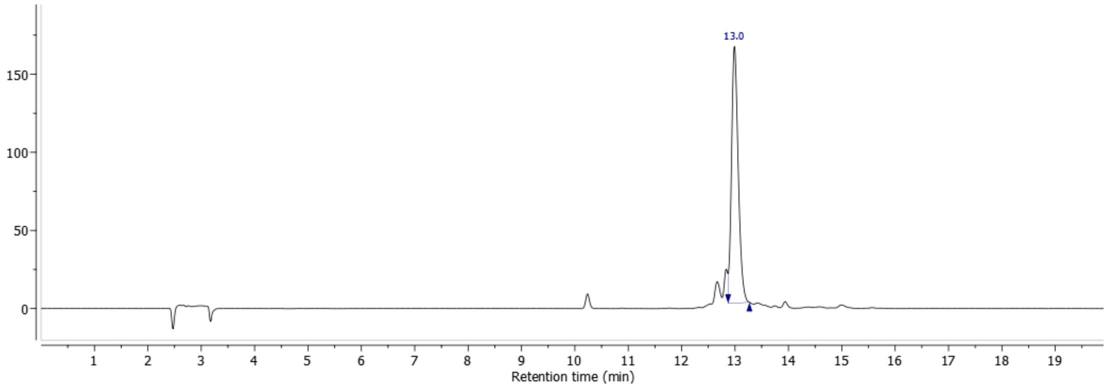

Figure S37. RP-HPLC trace of the pure bombesin peptide **19**.

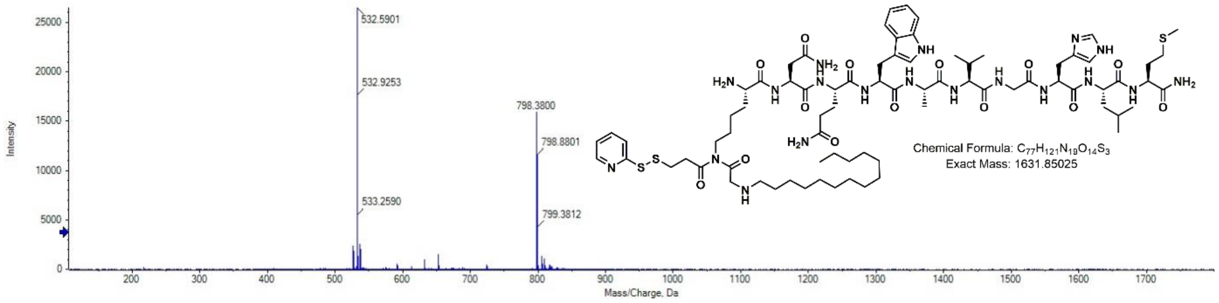

Figure S38. ESI-HRMS of pure bombesin peptide **19**.

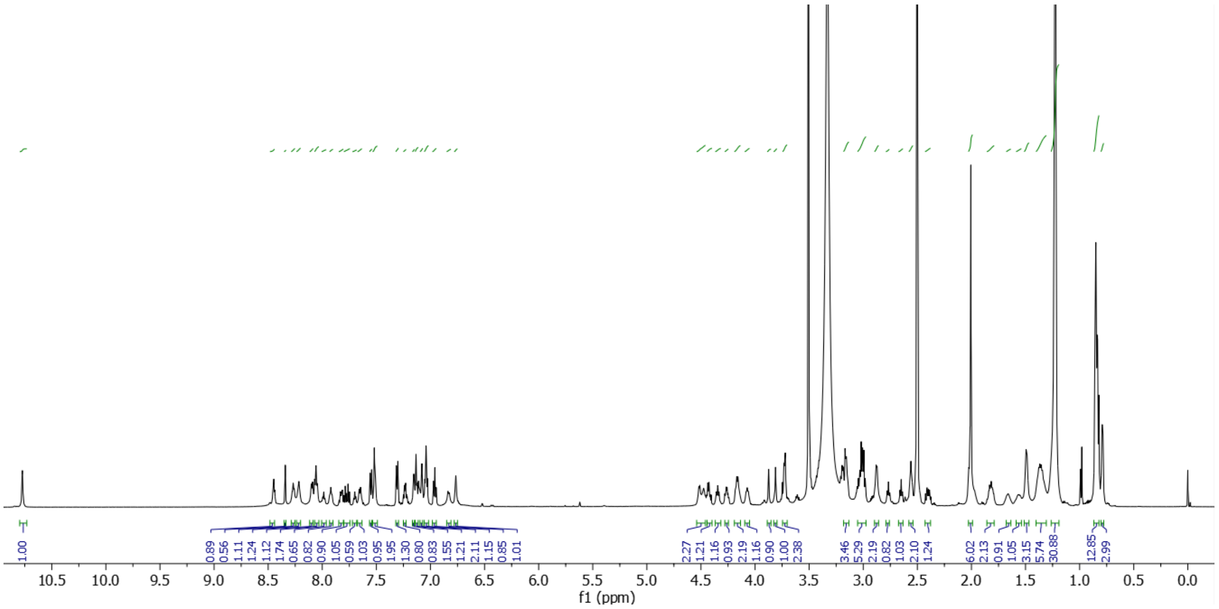

Figure S39. ^{1}H NMR (400 MHz, DMSO-*d*6) spectrum of Bombesin peptide **19**.

Synthesis of bombesin-tubugi conjugates

Bombesin-tubugi conjugate 20

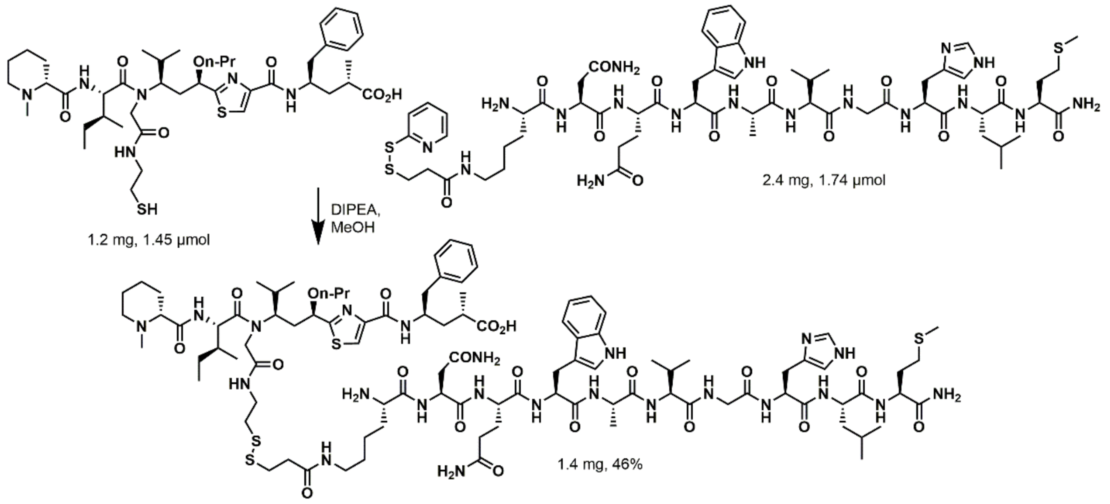

Bombesin peptide **18** (2.4 mg, 1.74 µmol) was dissolved in MeOH (1 mL) together with DIPEA (1 µL). Immediately afterwards, tubugi **10a** (1.2 mg, 1.45 µmol) was added and the mixture was stirred under a nitrogen atmosphere for 2 h. The crude mixture was directly purified by RP-HPLC to afford the bombesin-tubugi conjugate **20** (1.4 mg, 46%) as a white amorphous solid. R_t = 12.8 min. HR-MS *m/z*: 700.3626 $[M+3H]^{3+}$, calcd. for $C_{98}H_{154}N_{23}O_{20}S_4$: 700.3541.

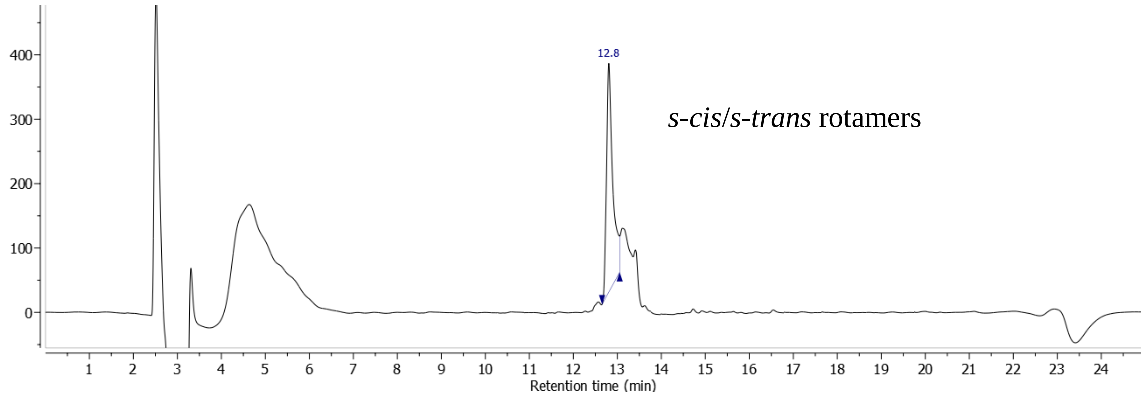

Figure S40. RP-HPLC trace of the pure bombesin peptide **20**.

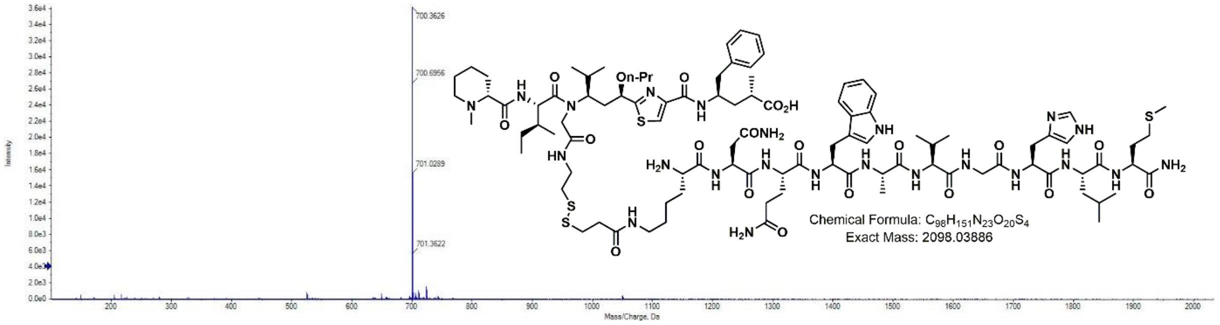

Figure S41. ESI-HRMS of tubugi-bombesin conjugate **20**.

Bombesin-tubugi conjugate 21

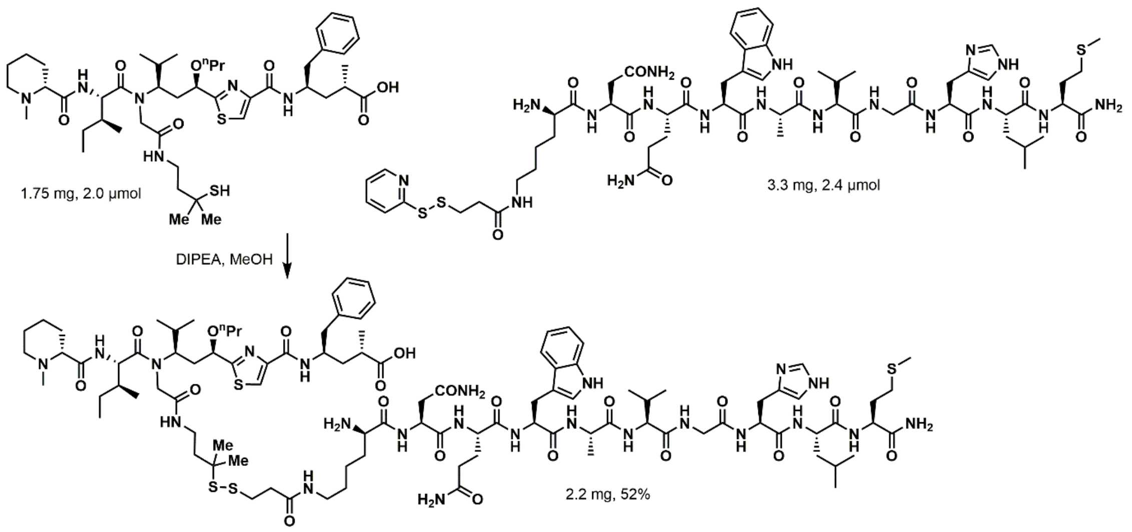

Bombesin peptide **18** (3.3 mg, 2.4 µmol) was dissolved in MeOH (1 mL) together with DIPEA (1 µL). Immediately afterwards, tubugi **10b** (1.75 mg, 2.0 µmol) was added and the mixture was stirred under a nitrogen atmosphere for 2 h. The crude mixture was directly purified by RP-HPLC to afford the bombesin-tubugi conjugate **21** (2.2 mg, 52%) as a white amorphous solid. R_t = 13.2, 13.6. HR-MS *m/z*: 714.3661 $[M+3H]^{3+}$, calcd. for $C_{101}H_{160}N_{23}O_{20}S_4$: 714.3693.

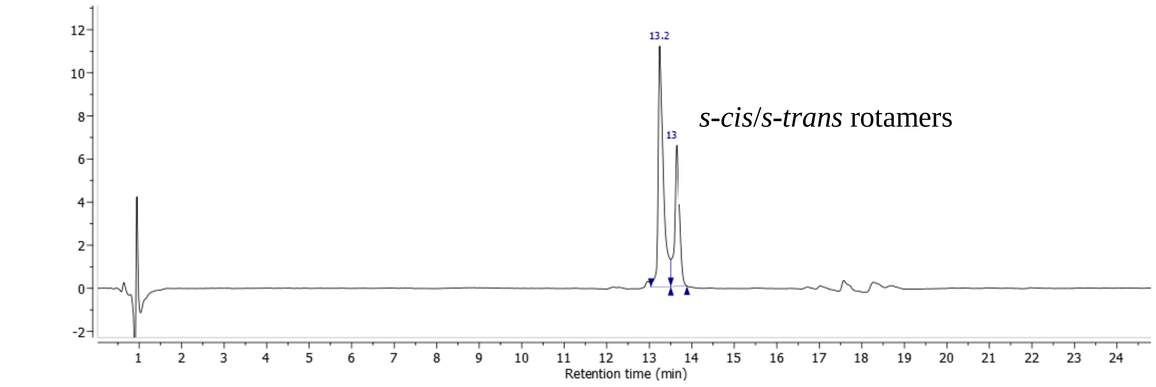

Figure S42. RP-HPLC trace of the pure bombesin peptide **21**.

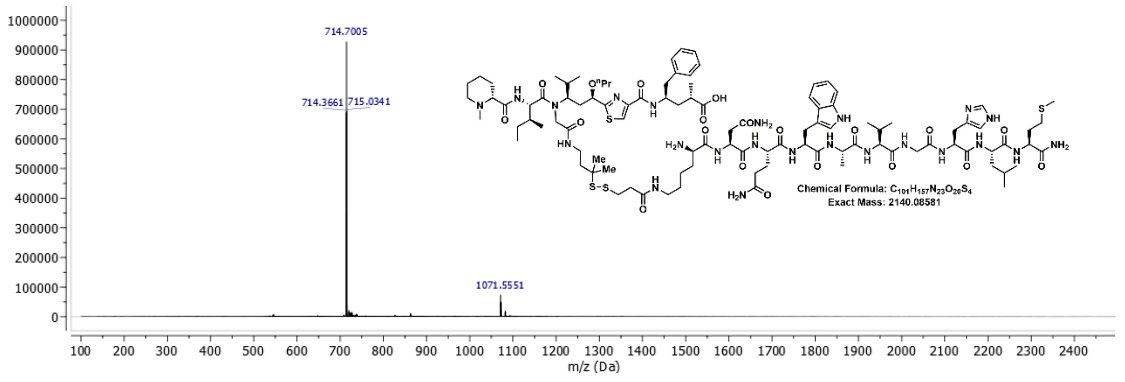

Figure S43. ESI-HRMS of tubugi-bombesin conjugate **21**.

Bombesin-tubugi conjugate 22

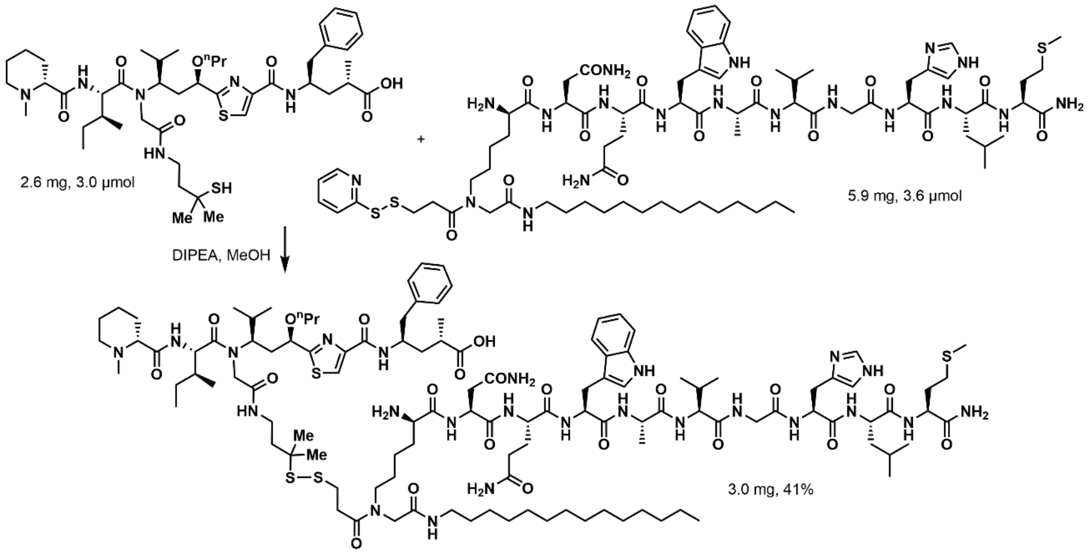

Bombesin peptide **19** (5.9 mg, 3.6 µmol) was dissolved in MeOH (1 mL) together with DIPEA (1 µL). Immediately afterwards, tubugi **10b** (2.6 mg, 3.0 µmol) was added and the mixture was stirred under a nitrogen atmosphere for 2 h. The crude mixture was directly purified by RP-HPLC to afford the bombesin-tubugi conjugate **21** (3.0 mg, 41%) as a white amorphous solid. R_t = 12.9 min. HR-MS *m/z*: 798.7791 $[M+3H]^{3+}$, calcd. for $C_{115}H_{187}N_{24}O_{21}S_4$: 798.7833.

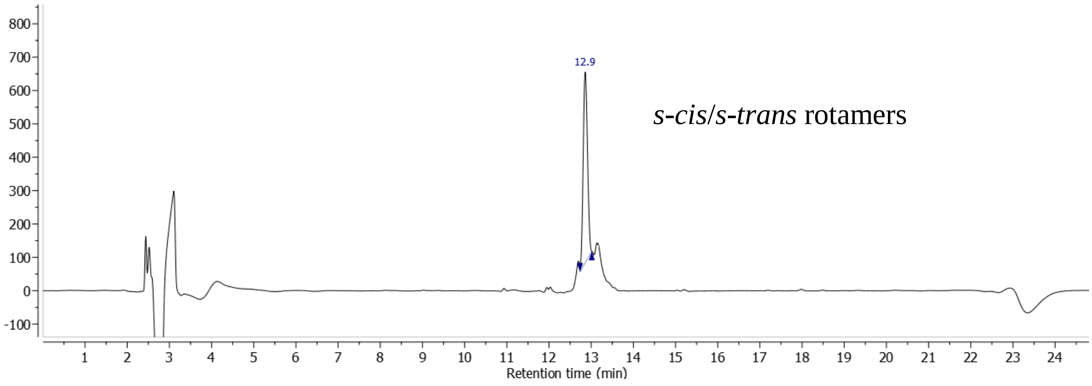

Figure S44. RP-HPLC trace of the pure bombesin peptide **22**.

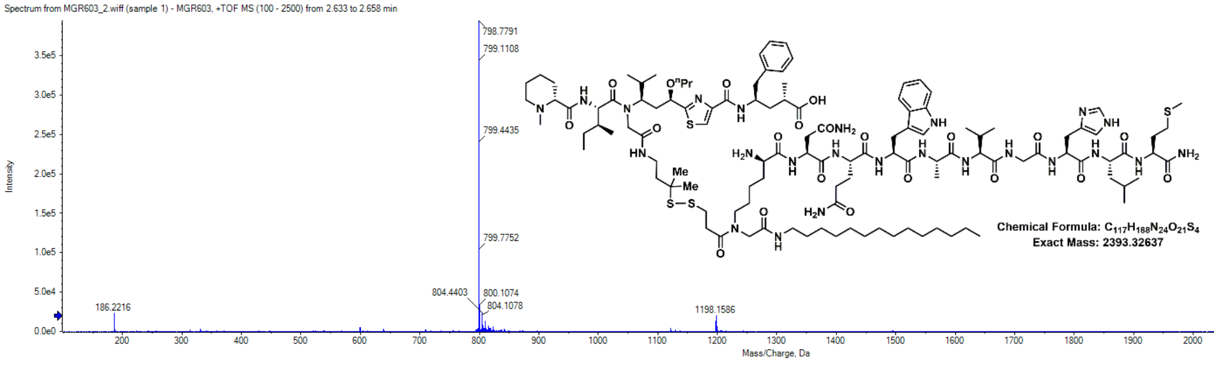

Figure S45. ESI-HRMS of tubugi-bombesin conjugate **22**.

In vitro RT-qPCR for receptor expression analysis

Since the novel bombesin analogues and peptide-tubugi conjugates described herein have been intended to address and target gastrin-releasing peptide receptor (GRPR)-overexpressing tumor cells, the investigated human cell lines have been selected, based on RNA-Seq data analyzed by using the Genevestigator® gene expression analysis tool (Zurich, Switzerland),(5) to cover a broad range of GRPR-overexpressing to GPCR-lowexpressing cells. The actual GRPR mRNA expression of the used cell lines' batches were proven by conducting a RT-qPCR analysis. For that purpose, the mRNA of standardly cultured, untreated cells was isolated using a quick-RNA miniprep kit purchased from Zymo Research (Freiburg i. Br., Germany) and was checked for its concentration and integrity by using a SpectraDrop™ with SpectraMax iD5 (Molecular Devices, San Jose, CA, USA) and 0.5% (w/v) agarose gel electrophoresis, respectively. RevertAid Reverse Transcription (RT) kit and oligo-dT primer from Thermo Fisher Scientific (Waltham, MA, USA) were used with 0.5 µg of the isolated mRNA to synthesize first strand cDNA that was used for subsequent qPCR analyses. QPCR primers, specific for the target receptor hGRPR and the housekeeping gene hGAPDH (glyceraldehyde 3-phosphate dehydrogenase) were designed by using NCBI's Primer-Blast and purchased from Eurofins Genomics (Ebersberg, Germany). The primer sequences are shown in Table S1. The qPCR analyses were conducted by using the GreenMaster mix from Jena Bioscience (Jena, Germany) and BioRad's CFX96TM Real-Time PCR Detection System (Hercules, CA, USA). The qPCR data were processed according to the ΔΔCt methodology using GAPDH as reference gene. RNA isolation, cDNA synthesis and qPCR were performed in accordance with the manufacturers' guidelines.

Table S1. QPCR primers for the relative quantification of the cell lines' hGRPR (human gastrin-releasing peptide receptor) mRNA expression levels.

**Primer**	**Target**	**Sequence (5' – 3')**	**Tm (°C)**	**Amplicon (bases)**
hGRPR_854_s	human GRPR; sense	TGT CTG TCT TCA CAC TCA CG	57.3	156
hGRPR_1024_as	human GRPR; antisense	TGG AGA TGA TCC AGA TAA AGG	55.9	
hGAPDH_194_s	human GAPDH; sense	TTG CCA TCA ATG ACC CCT TCA	57.4	190
hGAPDH_350_as	human GAPDH; antisense	CGC CCC ACT TGA TTT TGG A	55.6	

13

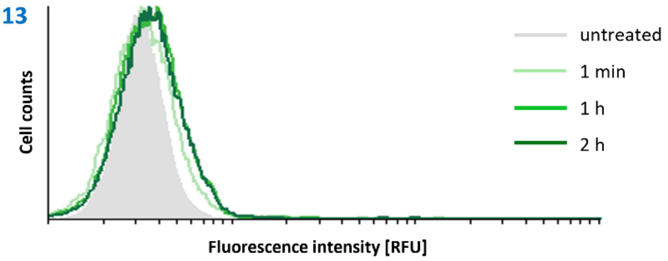

Comp. 13	RFU mean	X-fold over untreated
untreated	3.5	-
1 min	3.9	1.1
1 h	4.1	1.2
2 h	4.2	1.2

15a

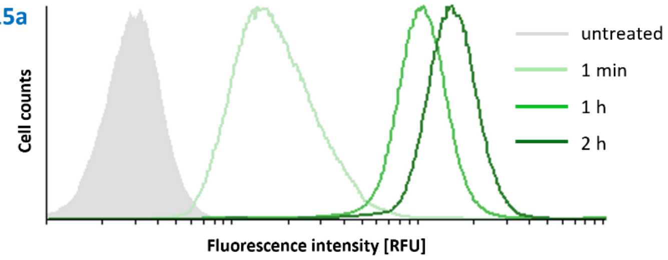

Comp. 15a	RFU mean	X-fold over untreated
untreated	3.5	-
1 min	22.4	6.4
1 h	127.0	36.3
2 h	183.8	52.5

15b

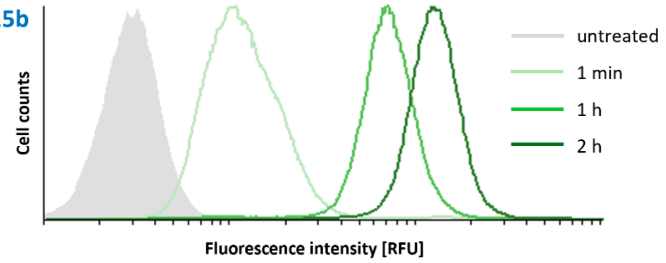

Comp. 15b	RFU mean	X-fold over untreated
untreated	3.5	-
1 min	16.4	4.7
1 h	86.5	24.7
2 h	153.3	43.8

15c

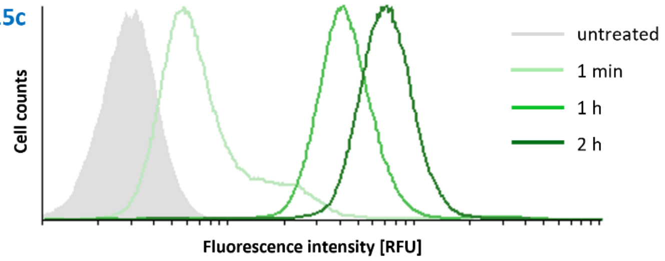

Comp. 15c	RFU mean	X-fold over untreated
untreated	3.5	-
1 min	12.2	3.5
1 h	54.5	15.6
2 h	86.9	24.8

15d

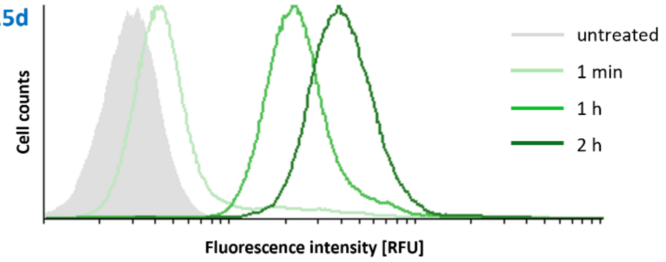

Comp. 15d	RFU mean	X-fold over untreated
untreated	3.5	-
1 min	9.0	2.6
1 h	30.5	8.7
2 h	51.3	14.7

Figure S46. Representative flow cytometric histograms illustrating the incubation time dependency of the cell internalization of the bombesin analogues **13**, **15a**, **15b**, **15c** and **15d** into human prostate cancer PC-3 cells at 37°C. The right columns of the tables – –measure of the compounds' cell internalization – represents the normalized ratio calculated based on the measured relative fluorescence units (RFU) of the compound-treated cells compared to the untreated control cells.

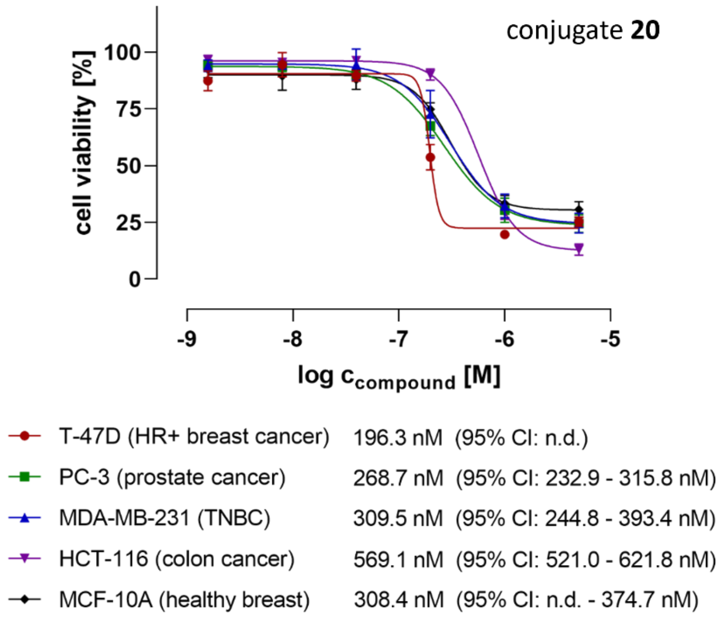

Figure S47. Cell viability of human cancer (T-47D, PC-3, MDA-MB-231, and HCT-116) and non-cancer (MCF-10A) cell lines after 48 h initial treatment with the bombesin-tubugi conjugate **20**, medium exchange and subsequent cell cultivation until finalization of 72 h, and subsequent measurement by using fluorometric resazurin assay read-out. Each data point was determined in at least 3-4 biological replicates each with technical triplicates. The indicated concentrations are IC_{50} values as calculated based on the non-linear regression dose-response curves as drawn by using GraphPad Prism 8 software. Error bars indicate the 95% confidence interval.

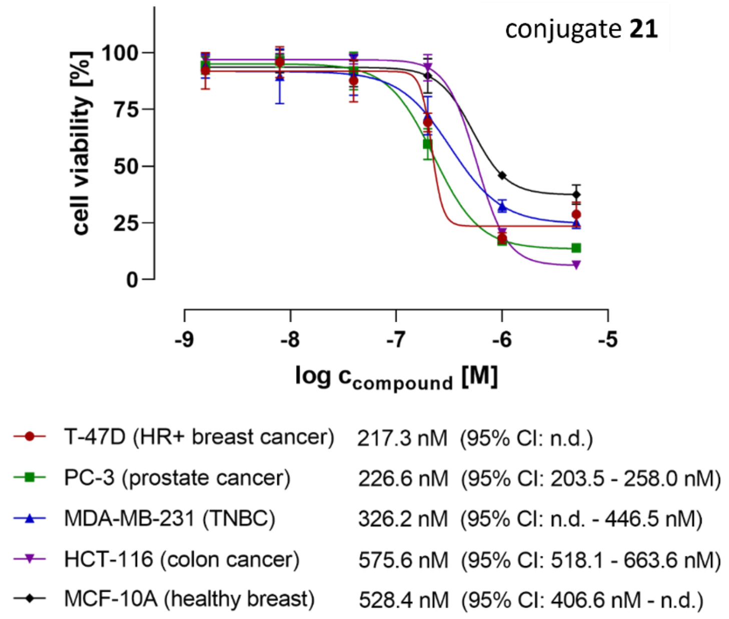

Figure S48. Cell viability of human cancer (T-47D, PC-3, MDA-MB-231, and HCT-116) and non-cancer (MCF-10A) cell lines after 48 h initial treatment with the bombesin-tubugi conjugate **21**, medium exchange and subsequent cell cultivation until finalization of 72 h, and subsequent measurement by using fluorometric resazurin assay read-out. Each data point was determined in at least 3-4 biological replicates each with technical triplicates. The indicated concentrations are IC_{50} values as calculated based on the non-linear regression dose-response curves as drawn by using GraphPad Prism 8 software. Error bars indicate the 95% confidence interval.

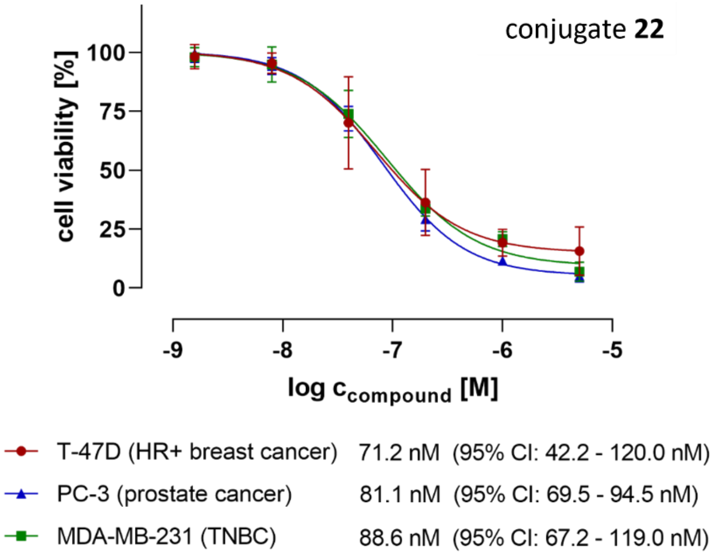

Figure S49. Cell viability of human cancer cell lines (T-47D, PC-3, and MDA-MB-231) after 48 h initial treatment with the bombesin-tubugi conjugate **22**, medium exchange and subsequent cell cultivation until finalization of 72 h, and subsequent measurement by using fluorometric resazurin assay read-out. Each data point was determined in at least 3-4 biological replicates each with technical triplicates. The indicated concentrations are IC_{50} values as calculated based on the non-linear regression dose-response curves as drawn by using GraphPad Prism 8 software. Error bars indicate the 95% confidence interval.

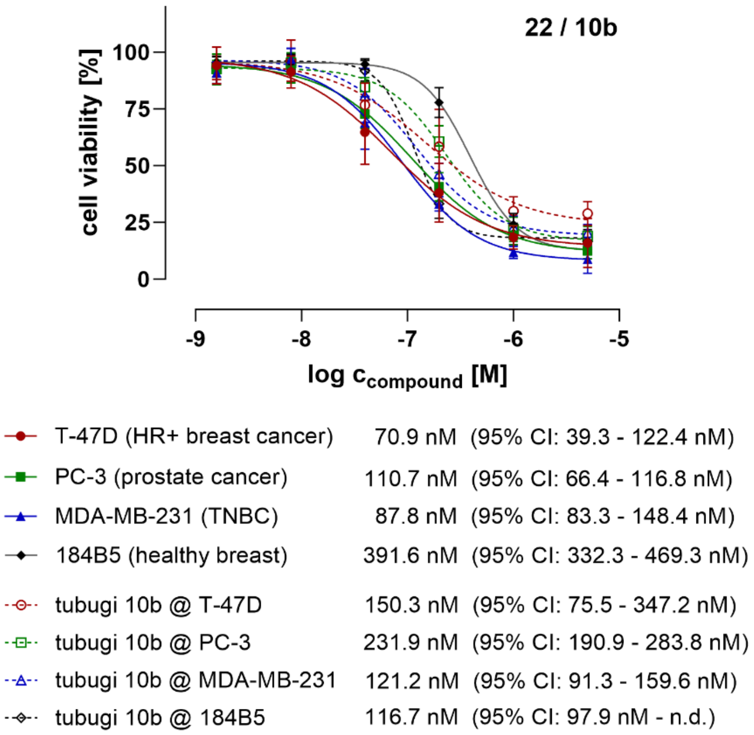

Figure S50. Cell viability of human cancer (T-47D, PC-3, MDA-MB-231) and non-cancer (184B5) cell lines after 6 h initial treatment with the bombesin-tubugi conjugate **22** and its non-conjugated tubugi payload **10b**, medium exchange and subsequent cell cultivation until finalization of 72 h, and subsequent measurement by using fluorometric resazurin assay read-out. Each data point was determined in at least 3-4 biological replicates each with technical triplicates. The indicated concentrations are IC_{50} values as calculated based on the non-linear regression dose-response curves as drawn by using GraphPad Prism 8 software. Error bars indicate the 95% confidence interval.

References

1. Ricardo MG, Marrero JF, Valdes O, Rivera DG, Wessjohann LA. A Peptide Backbone Stapling Strategy Enabled by the Multicomponent Incorporation of Amide *N*-Substituents. *Chem - A Eur J* (2018) 25:769–774.

2. Ricardo MG, Vasco A V., Rivera DG, Wessjohann LA. Stabilization of Cyclic β-Hairpins by Ugi-Reaction-Derived *N*-Alkylated Peptides: The Quest for Functionalized β-Turns. *Org Lett* (2019) 21:7307–7310.

3. Digilio G, Menchise V, Gianolio E, Catanzaro V, Carrera C, Napolitano R, Fedeli F, Aime S. Exofacial protein thiols as a route for the internalization of Gd(III)-based complexes for magnetic resonance imaging cell labeling. *J Med Chem* (2010) 53:4877–4890.

4. E. Kaiser, R.L. Colescott, C.D. Bossinger PIC. Color test for detection of free terminal amino groups in the solid-phase synthesis of peptides. *Anal Biochem* (1970) 34:595–598.

5. Hruz T, Laule O, Szabo G, Wessendorp F, Bleuler S, Oertle L, Widmayer P, Gruissem W, Zimmermann P. Genevestigator V3: A Reference Expression Database for the Meta-Analysis of Transcriptomes. *Adv Bioinforma* (2008)420747.
